# Improving outcomes for children with malaria, diarrhoea and pneumonia in Mozambique: A cluster randomised controlled trial of the inSCALE technology innovation

**DOI:** 10.1371/journal.pdig.0000235

**Published:** 2023-06-12

**Authors:** Seyi Soremekun, Karin Källander, Raghu Lingam, Ana-Cristina Castel Branco, Neha Batura, Daniel Ll Strachan, Abel Muiambo, Nelson Salomao, Juliao Condoane, Fenias Benhane, Frida Kasteng, Anna Vassall, Zelee Hill, Guus ten Asbroek, Sylvia Meek, James Tibenderana, Betty Kirkwood

**Affiliations:** 1 Department of Infection Biology, London School of Hygiene & Tropical Medicine, Keppel Street, London, United Kingdom; 2 Malaria Consortium, The Green House, 244–254 Cambridge Heath Road, London, United Kingdom; 3 Department of Global Public Health, Karolinska Institutet, Stockholm, Sweden; 4 UNICEF Programme Division, Health Section, New York, New York State, United States of America; 5 Population Child Health Research Group, School of Women’s and Children’s Health, University of New South Wales, Australia; 6 Malaria Consortium, Rua Joseph Ki-Zerbo 191, PO Box 3655, Coop, Maputo, Mozambique; 7 Institute for Global Health, University College London, 30 Guilford Street, London, United Kingdom; 8 The Nossal Institute for Global Health, Melbourne School of Population and Global Health, The University of Melbourne Victoria, Australia; 9 Department of Global Health and Development, London School of Hygiene & Tropical Medicine, Keppel Street, London, United Kingdom; 10 Department of Global Health, Amsterdam University Medical Centres, Meibergdreef 9 1105 AZ Amsterdam, The Netherlands and Amsterdam Institute for Global Health and Development, Paasheuvelweg 25, 1105 BP Amsterdam, The Netherlands; 11 Department of Population Health, London School of Hygiene & Tropical Medicine, Keppel Street, London, WC1E 7HT, United Kingdom; Flinders University, AUSTRALIA

## Abstract

**Background:**

The majority of post-neonatal deaths in children under 5 are due to malaria, diarrhoea and pneumonia (MDP). The WHO recommends integrated community case management (iCCM) of these conditions using community-based health workers (CHW). However iCCM programmes have suffered from poor implementation and mixed outcomes. We designed and evaluated a technology-based (mHealth) intervention package ‘inSCALE’ (Innovations At Scale For Community Access and Lasting Effects) to support iCCM programmes and increase appropriate treatment coverage for children with MDP.

**Methods:**

This superiority cluster randomised controlled trial allocated all 12 districts in Inhambane Province in Mozambique to receive iCCM only (control) or iCCM plus the inSCALE technology intervention. Population cross-sectional surveys were conducted at baseline and after 18 months of intervention implementation in approximately 500 eligible households in randomly selected communities in all districts including at least one child less than 60 months of age where the main caregiver was available to assess the impact of the intervention on the primary outcome, the coverage of appropriate treatment for malaria, diarrhoea and pneumonia in children 2-59months of age. Secondary outcomes included the proportion of sick children who were taken to the CHW for treatment, validated tool-based CHW motivation and performance scores, prevalence of cases of illness, and a range of secondary household and health worker level outcomes. All statistical models accounted for the clustered study design and variables used to constrain the randomisation. A meta-analysis of the estimated pooled impact of the technology intervention was conducted including results from a sister trial (inSCALE-Uganda).

**Findings:**

The study included 2740 eligible children in control arm districts and 2863 children in intervention districts. After 18 months of intervention implementation 68% (69/101) CHWs still had a working inSCALE smartphone and app and 45% (44/101) had uploaded at least one report to their supervising health facility in the last 4 weeks. Coverage of the appropriate treatment of cases of MDP increased by 26% in the intervention arm (adjusted RR 1.26 95% CI 1.12–1.42, p<0.001). The rate of care seeking to the iCCM-trained community health worker increased in the intervention arm (14.4% vs 15.9% in control and intervention arms respectively) but fell short of the significance threshold (adjusted RR 1.63, 95% CI 0.93–2.85, p = 0.085). The prevalence of cases of MDP was 53.5% (1467) and 43.7% (1251) in the control and intervention arms respectively (risk ratio 0.82, 95% CI 0.78–0.87, p<0.001). CHW motivation and knowledge scores did not differ between intervention arms. Across two country trials, the estimated pooled effect of the inSCALE intervention on coverage of appropriate treatment for MDP was RR 1.15 (95% CI 1.08–1.24, p <0.001).

**Interpretation:**

The inSCALE intervention led to an improvement in appropriate treatment of common childhood illnesses when delivered at scale in Mozambique. The programme will be rolled out by the ministry of health to the entire national CHW and primary care network in 2022–2023. This study highlights the potential value of a technology intervention aimed at strengthening iCCM systems to address the largest causes of childhood morbidity and mortality in sub-Saharan Africa.

## Author summary

The inSCALE cluster-randomised trial in Mozambique was part of a $10million project funded by the Bill and Melinda Gates Foundation to design and test innovative primary care interventions to improve health outcomes for children with malaria, diarrhoea and pneumonia (MDP), which together are the largest killers of children aged <5yrs. The study aimed to strengthen the primary health care system with a focus on community health workers, representing the most accessible level of care for many underserved populations. We designed a technology-based intervention delivered using low-end smartphones. This intervention was based on mHealth principles and included electronic WHO algorithms with basic AI functions for community health workers (CHWs) to guide correct diagnosis and treatment of MDP, provided personalised feedback to health workers, and alerts to supervising health facilities on stock outs, data tracking and CHW competencies. The study was implemented within the entire province of Inhambane, and districts were randomly assigned to the intervention or to continue with standard care (control). Compared to control districts, we observed significant reductions in the prevalences of MDP in children under 5 years (reductions of 20% for malaria, 34% for pneumonia, and 45% for diarrhoea) and an increase in appropriate treatment of any cases of MDP by 26% (of all cases MDP) and 40% (of all children) in the intervention districts. As a result of this trial, the government of Mozambique incorporated the inSCALE intervention into its policy for child health services, and is in the process of scaling up the programme to all 8000+ CHWs across the country (2022–2023).

## Introduction

Globally more than 5 million mostly preventable deaths occur in children under 5 years of age every year [1]. The vast majority of post neonatal deaths in this group are due to pneumonia, diarrhoea, malaria or malnutrition [2]. It is acknowledged that urgent work is required to address the causes and more widely, the systems within which such deaths occur. However progress has been uneven; under 5 mortality rates in sub Saharan Africa today remain similar to or higher than 1990 mortality rates in most high income countries with an average rate of 78 deaths per 1000 live births across the continent in 2018 [2]. Lack of access to timely and appropriate clinical management of sick children is a key factor driving poor health outcomes [3,4] prompting a World Health Organisation/UNICEF joint statement in 2012 advocating for integrated community case management (iCCM) of malaria, diarrhoea and pneumonia delivered by community-based health workers (CHW), who would be trained to diagnose and treat key childhood conditions in areas with poor access to facility-based services [3]. In the decade following this statement, iCCM programmes have been introduced into more than 50 low- and middle-income countries (LMICs) [5–7]. Whilst a recent multi-country modelling study in DRC, Malawi, Niger, and Nigeria [8] estimated that iCCM may have been responsible for averting nearly 5000 deaths per year in these countries combined due to malaria, diarrhoea and pneumonia, this estimate varied considerably between countries, and was in contrast to a Cochrane systematic review which found no clear impacts of iCCM on morbidity and mortality [9]. Both studies reported poor or uneven implementation of iCCM programmes and a need for further studies that addressed structural deficiencies in health systems and processes that hamper success and scale-up.

Central to the challenge in implementation of iCCM has been the lack of sufficient support for CHW networks, high rates of CHW attrition, low rates of reported motivation and poor quality of disease management practices [10]. The WHO calls for “innovative and tailored approaches” to strengthening child health services [1], yet there are few programmes or even research studies which have explicitly addressed structural barriers in the context of scaling up iCCM effectively.

Digital technologies, and specifically mHealth (the use of mobile technologies in medical and public health practice [11]) is an area of growing interest for the delivery of health services in sub-Saharan Africa [12,13] particularly given the rapid expansion of mobile phone networks and usage in this setting [14]. We designed a systems-strengthening package of programmes collectively called”inSCALE” (interventions at scale for community access and lasting effects). inSCALE was a multi-country study of innovative interventions implemented by the Malaria Consortium in partnership with the governments of Mozambique and Uganda and supported by international funders (the Bill and Melinda Gates Foundation, UNICEF, and the Canadian international Development Agency now Global Affairs Canada (GAC)) and co-designed with academic partners (The London School of Hygiene and Tropical Medicine and University College, London). Reported in this paper are the findings of the inSCALE technology intervention, a disease management support package developed following extensive formative research and building on the study conceptual framework [15]. The package was primarily delivered on Samsung Galaxy smartphones (“the inSCALE app”) that included electronic clinical decision algorithms, personalised reports, messaging, and feedback to CHWs and health facility staff. The Mozambique site tested the inSCALE technology intervention only; the Uganda site tested both the technology intervention and a community-based intervention described elsewhere [16]. The technology interventions in both countries sought to specifically address primary health system challenges that could lead to improved management of key childhood illnesses through first improving CHW performance, motivation and reducing attrition. A detailed description of the development of the inSCALE technology intervention, rationale and conceptual framework is provided elsewhere [15]. The primary aim of the trial was therefore to assess the impact of the inSCALE technology intervention on the coverage of the appropriate treatment of childhood malaria, diarrhoea and pneumonia. This paper reports on the impacts of the technology intervention in Mozambique to accompany the separate impact analysis for the Uganda inSCALE trial [16] and conducts a meta-analysis of pooled impact of the technology intervention across its sites in Uganda and Mozambique.

## Results

**Fig 1:**
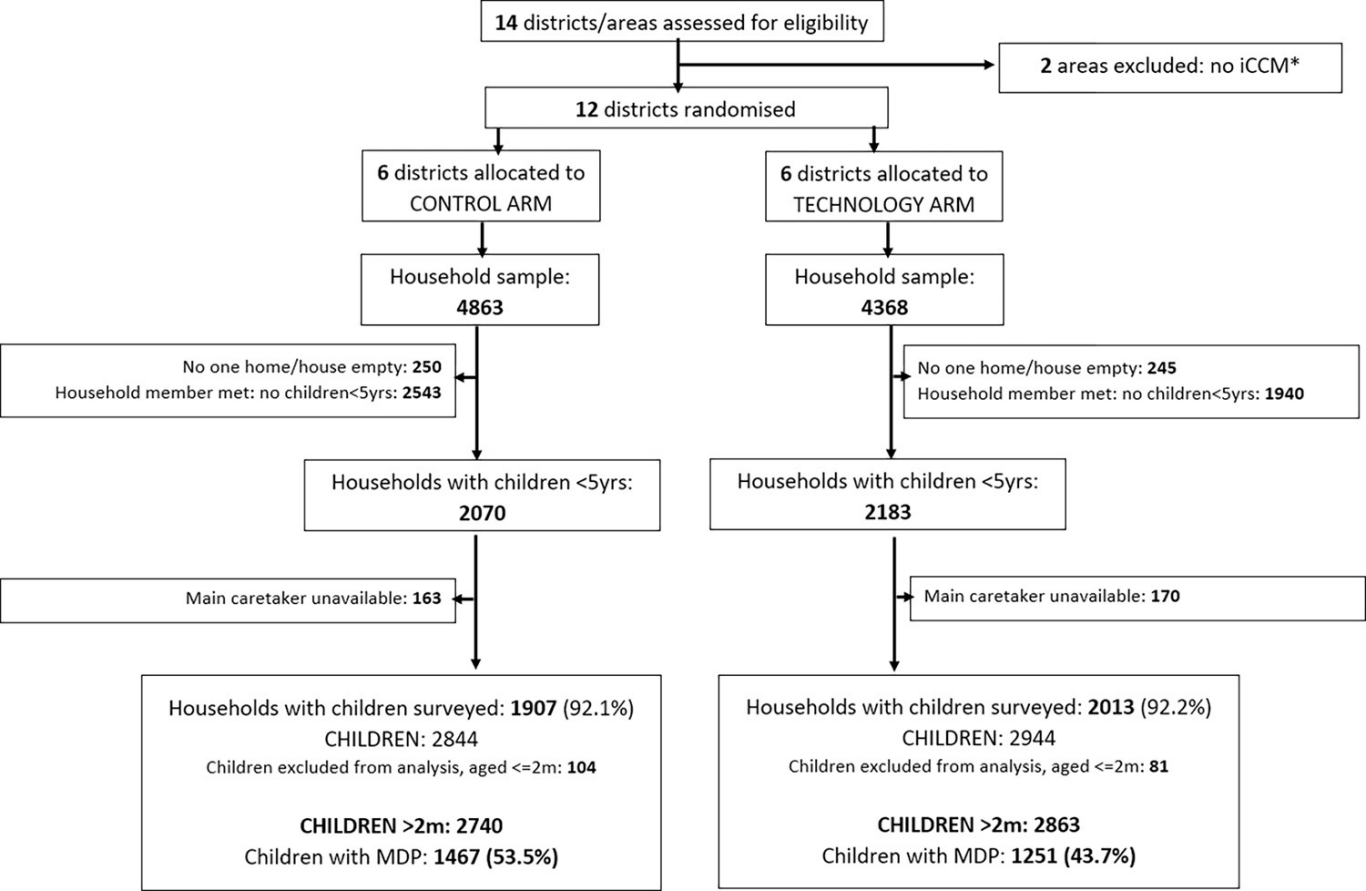
Flow diagram of participant progression through trial phases. Final analysis sample: children aged 2 months to 5 years with suspected malaria, diarrhoea or suspected pneumonia (MDP). * Maxixe and Inhambane, the economic and provincial capitals were excluded from the study (do not implement iCCM).

In total 9231 households in the 12 districts were assessed for eligibility and of these, 495 were empty or no one was home, and 4483 did not contain children under 5 years of age. Overall, the main caretaker was available in 92% (3920) of houses with children under 5 years of age. At the endline survey, a total of 2718 children had had symptoms of suspected malaria, diarrhoea or pneumonia (MDP) in the past month (the impact analysis sample), of which 1467 were in the control arm and 1251 in the intervention arm (Fig 1 flow chart). Household characteristics of sick children were fairly well balanced between arms with small variations in some parameters–Table 1.

**Table 1:** Characteristics of the inSCALE Mozambique study sample.

Characteristic	Control % (n)	Technology % (n)
**Eligible children**	**N = 2740**	**N = 2863**
*%* children with MDP	53.5% (1467)	43.7% (1251)
*%* children with suspected malaria*	48.5% (1300)	39.8% (1118)
*%* children with confirmed malaria*	24.6% (661)	21.8% (612)
*%* children with diarrhoea**	7.2% (198)	4.2% (121)
*%* children with suspected pneumonia**	16.2% (443)	10.6% (304)
**Children with MDP**	**N = 1467**	**N = 1251**
**% Respondent is mother of sick child**	81% (1181)	83% (1036)
**% children are boys**	50% (733)	50% (629)
**Age of mother**		
12–19	10% (144)	8% (104)
20–29	38% (552)	37% (467)
30–39	28% (413)	30% (373)
40–49	12% (181)	14% (175)
50+	8% (113)	6% (76)
Not known	4% (64)	4% (56)
**Highest education level completed (respondent)**		
None	31% (453)	32% (400)
Incomplete primary	48% (706)	45% (557)
Primary or higher	21% (308)	24% (294)
**Mother tongue**		
Bitonga	17% (246)	6% (81)
Chitsua	54% (793)	72% (900)
Chichopi	17% (254)	19% (242)
Other	12% (174)	2% (28)
**Marital status**		
Married	2% (34)	3% (41)
Living with partner (not married)	78% (1151)	74% (929)
Single/divorced/widowed	19% (282)	22% (281)
**% Main occupation is farming/fishing**	75.9% (1113)	64.0% (801)

*Denominator is the 5490 children aged 4months– 5 years

**Denominator is the 5603 children aged 2m – 5 year

The restricted randomisation method resulted in good balance between intervention arms for balance variables as described in the protocol paper [15], which were care seeking rates, CHW motivation, and cost of seeking care (Table 2). Differences for all variables was either less than 5% (percentages), or less than 0.1 (means).

**Table 2:** Key indicators used to balance arms at baseline.

Variables included in the restricted randomisation*	Control arm 662 children with MDP 128 CHWs	Technology 658 children with MDP 128 CHWs	Difference
Mean (log_10_) cost of care seeking for children with MDP (SD)	1.11 (0.09)	1.14 (0.14)	0.03
Cluster mean % care seeking to an CHW for children with MDP (SD)	22.5% (23.03)	22.8% (16.92)	0.26
Cluster mean % care seeking to a public facility for children with MDP (SD)	50.96% (21.63)	48.79% (14.80)	2.17
Cluster mean CHW motivation score (SD)	2.29 (0.46)	2.38 (0.50)	0.09

*Outcomes are presented as mean percentages or mean costs across all clusters in each arm

### Intervention coverage, sickness and care seeking

By the end of the 18-month implementation period, 68% of CHWs reported having a working inSCALE phone and app, and 45% had sent a report via the app in the last 4 weeks (Table 3). Whilst overall prevalence of MDP was similar between arms at baseline, there were significant reductions in the prevalence of suspected malaria, diarrhoea and pneumonia (reductions ranging from 18% to 45%), and a 16% increase in the prevalence of diagnostic testing for malaria in the intervention arm at endline (Table 4). Care seeking to the CHW as the first port of call was 14.4% in the control arm and 15.9% in the intervention arm. After adjusting for the restricted randomisation and clustered design this represented a 63% increase in care-seeking to the CHW, a result that just fell short of significance (Table 5, p = 0.085). There were no significant differences in care seeking rates to other providers or in the prevalence of children for whom no care was sought (Table 5).

**Table 3:** Exposure to intervention components recorded during the endline survey.

Intervention component	Technology
**CHWs**	**N = 104**
% CHWs had a working phone and inSCALE app at endline (n)	68% (69)*
% CHWs submitted at least one inSCALE report through their phone in the last 4 weeks	45.2% (44)*

*data missing for 3 CHWs

**Table 4:** Prevalence of suspected malaria, diarrhoea and pneumonia (MDP) and blood tests for malaria in children under 5 years at baseline and endline.

Prevalence of	Baseline (reported prevalence in last 2 weeks)		Endline (reported prevalence in last 4 weeks)		Impact of inSCALE at endline Risk ratio inSCALE/control RR: 95% CI; pᵜ
	Control arm	Technology arm	Control arm	Technology arm	
Any of MDP	29.3% (662/2257)	30.4% (658/2165)	53.5% (1467/2740)	43.7% (1251/2863)	**0.82** (0.78–0.87) p<0.001
Suspected malaria*	25.9% (562/2171)	26.7% (556/2084)	48.5% (1300/2682)	39.8% (1118/2808)	**0.80** (0.72–0.89) p<0.001
Blood test for malaria**	31.7% (200/631)	29.5% (181/613)	59.7% (937/1570)	62.3% (831/1335)	**1.16** (1.06–1.28) p = 0.002
Confirmed malaria***	65.5% (131/200)	68.5% (124/181)	70.5% (661/937)	73.7% (612/831)	**1.04** (0.92–1.18) p = 0.514
Diarrhoea	5.1% (116/2257)	5.3% (114/2165)	7.2% (198/2740)	4.2% (121/2863)	**0.55** (0.42–0.71) p<0.001
Pneumonia	9.9% (223)	11.8% (256/2165)	16.2% (443/2740)	10.6% (304/2863)	**0.66** (0.44–0.99) p = 0.045

Prevalence (n/N) calculated as total eligible children 2 months-59 months of age (N) with condition in question (n), with the exception of fever and malaria where age range is 4 months-59 months in line with treatment guidelines.

*Fevers, excludes fevers with a negative blood test for malaria.

**denominator includes all children reporting fever in the last two (baseline) or 4 (endline) weeks: the exposure period was extended in the endline survey following recommendations for increasing accuracy of caregiver-reported outcomes published in the interim [17]

***percentage of all blood tested fevers that were positive. **ᵜ**Adjusted for i) parameters used to balance arms at baseline and ii) the baseline prevalence of prevalence parameter

**Table 5:** Care seeking choices for children with MDP at endline.

Outcome % of children (n)	Control (N = 1467)	Technology (N = 1251)	Risk ratio tech/control RR: 95% CI	p
% seeking care at a CHW (first point of call)	14.4% (211)	15.9% (199)	1.63 (0.93–2.85)	0.085
% seeking care at a CHW (at any point)	14.7% (215)	16.2% (203)	1.61 (0.91–2.85)	0.104
% Not seeking care outside the home	18.3% (269)	20.6% (251)	0.98 (0.74–1.30)	0.898
% seeking care at a public facility (at any point)	67.4% (988)	63.7% (797)	1.11 (0.93–1.33)	0.253
% seeking care in the private sector (at any point—private facility, pharmacy, shop, herbalist etc)	3.6% (53)	2.4% (30)	0.94 (0.51–1.71)	0.839

### Appropriate treatment

Baseline levels of appropriate treatment were similar for the combined MDP endpoint but varied by provider and for individual conditions between intervention arms (Table 6). At endline there was an 26% increase in appropriate treatment of cases of MDP (the primary outcome (Table 7) in the inSCALE arm compared to the control arm (95% confidence interval: 12%-42%, p<0.001). This rose to 30% when only episodes treated with recommended first line treatments were defined as appropriately treated (Table 7 95% CI: 12%-52%, p = 0.001). A breakdown of appropriate treatment by provider suggests this impact was driven by significant improvements in appropriate treatment coverage in public health facilities (Table 7) which were the first port of call for caregivers for more than 60% of sick children in both arms (Table 5), and in the appropriate treatment of diarrhoea, where a 76% increase (95% CI 20%-256% p = 0.003) in the use of oral rehydration salts was observed in the intervention arm (Table 8). Note it was not possible to estimate a confidence interval for use of ORS plus zinc due to small cell sizes. Additional improvements came in the use of the recommended first line antibiotic for pneumonia (50% increase in prescribing of amoxicillin in the intervention arm 95% CI: 3%-217%). A whole-child analysis (Table C in S1 Text) estimated an improvement of 40% in the proportion of children appropriately treated for all MDP condition(s) reported in the last 4 weeks in the intervention arm (95% CI: 9% - 79% p = 0.009).

**Table 6:** Primary Outcome at baseline: Percentage of episodes of MDP that were appropriately treated.

Outcome	Control	Technology
	% (n/N*)	% (n/N*)
**Total children in baseline sample**	**2257**	**2165**
**% children with MDP** **	29.3% (662/2257)	30.4% (658/2165)
**Appropriate treatment**		
**Any episode of MDP**	**33.1% (298/901)**	**28.2% (261/926)**
Suspected malaria	31.3% (176/562)	28.4% (158/556)
Confirmed malaria	90.1% (118/131)	88.7% (110/124)
Diarrhoea (ORS)	56.0% (65/116)	44.7% (51/114)
Diarrhoea (ORS and zinc)	2.6% (3/116)	2.6% (3/114)
Pneumonia (any antibiotic)	25.6% (57/223)	20.3% (52/256)
Pneumonia (amoxicillin)	10.3% (23/223)	10.6% (27/256)
Any episode of MDP at the CHW (first care seeking location)	35.7% (71/199)	32.0% (70/219)
Any episode of MDP at the Public Facility (first care seeking location)	46.8% (214/457)	38.1% (171/449)
Any episode of MDP at the Private sector (first care seeking location, includes private facility, pharmacy, shop, herbalist)	18.8% (3/16)	19.2% (5/26)
Any episode of MDP—no care seeking outside of the home	10.1% (22/209)	6.2% (14/226)

*N = total episodes of the described disease; n = total episodes where the correct drugs were received.

**MDP: denominator includes all children with suspected or confirmed malaria, diarrhoea or pneumonia

**Table 7:** Appropriate treatment of MDP at endline: Appropriate treatment of episodes suspected malaria, diarrhoea or suspected pneumonia (MDP).

Appropriate treatment	Control		Technology		Technology v Control**	
	%	n/N*	%	n/N*	RR (95% CI)	p
Any episode of MDP	**47.8%**	**928/1941**	**50.7%**	**782/1543**	**1.26 (1.12–1.42)**	**<0.001**
Any episode of MDP (first line treatments only)***	**42.2%**	**819/1941**	**48.2%**	**744/1543**	**1.30 (1.12–1.52)**	**0.001**
**Appropriate treatment by first care seeking location (any episode of MDP)**						
CHW	49.5%	146/ 295	61.9%	148/239	1.04 (0.86–1.25)	0.675
Public Facility	58.0%	736/1269	62.2%	594/955	1.26 (1.07–1.48)	0.004
Private sector (private facility, pharmacy, shop, herbalist)	29.4%	10/34	21.7%	5/23	1.66 (0.52–5.34)	0.392
No care seeking outside of the home	10.5%	36/343	10.7%	35/326	1.23 (0.75–2.00)	0.408

*N = total episodes of the described disease; n = total episodes where the correct drugs were received

**adjusted for baseline levels of appropriate treatment for the condition(s) in question, and for variables used to balance arms at baseline (rates of facility and CHW care seeking, cost of care seeking, and CHW motivation score)

*** First line treatments: artemisinin combination therapy for malaria, ORS for diarrhoea, and amoxicillin for pneumonia

**Table 8:** Impact of the inSCALE intervention on appropriate treatment by condition.

Outcome	Control arm	Technology arm		
	Freq (%)	Freq (%)	risk ratio versus control (95% CI)	p
**Appropriate treatment by condition**				
Suspected malaria	54.4% (707/1300)	56.6% (633/1118)	1.08 (0.97–1.20)	0.150
Confirmed malaria	96.4% (637/661)	96.2% (589/612)	1.02 (0.99–1.04)	0.221
Diarrhoea (ORS)	29.3% (58/198)	43.8% (53/121)	1.76 (1.20–2.56)	0.003
Diarrhoea (ORS + zinc)	0.0% (0/198)	1.7% (2/121)	-	-
Suspected pneumonia (any antibiotic)	36.8% (163/443)	31.6% (96/304)	0.80 (0.60–1.08)	0.142
Suspected pneumonia (amoxycillin)	12.2% (54/443)	19.1% (58/304)	1.50 (1.03–2.17)	0.033

### CHW motivation, identification, clinical knowledge, attrition, and stockouts

The mean motivation and the mean social identification scores were relatively high and did not differ between arms (difference in motivation factors score: -0.03 95% CI -0.57, 0.51; difference in identification factor score: 0.01 95% CI -0.42, 0.43). CHW attrition rates were approximately 18%-19% in both intervention arms with no detected difference (Table 9). Overall scores for CHW clinical knowledge of the signs and management of MDP, also did not differ significantly between arms (Table 9). A breakdown of CHW knowledge scores by condition suggests a trend of improved knowledge in technology arm CHWs particularly in diagnosing pneumonia and diarrhoea—though none reached the threshold for statistical significance and may be chance findings (Table F in S1 Text). There were no between-arm differences in the rate of stockouts for key MDP treatments in the previous 3 months, though overall rates of stockouts were fairly high at >50% (Table 9).

**Table 9:** Community health worker (CHW) outcomes: Stockouts, attrition, motivation and knowledge in control and intervention arm clusters.

Outcome	Control arm (N = 103)	Technology arm (N = 104)	Effect size (95% CI)	p
	Mean (sd)	Mean (sd)		
% CHWs experienced a stockout of any MDP drug in last 3 months*	52.4% (54)	56.7% (59)	RR: 1.17 (0.92–1.49	0.207
Mean CHW motivation score	64.6 (5.9)	66.4 (5.5)	RD: +0.44 (-4.96, 5.85)	0.872
Mean CHW social identification score	17.4 (1.7)	17.8 (1.8)	RD: -0.03 (-1.52, 1.48)	0.972
Mean CHW knowledge score	19.7 (4.3)	19.8 (4.4)	RD: +0.97 (-2.01, 3.94)	0.524
Proportion CHW attrition (%)	18.4% (13.6)	18.5% (10.0)	RD: +1.31 (-18.66, 21.30)	0.878

* Stockouts of any of artemisinin combination therapy, amoxycillin, or ORS. RR- risk ratio; RD- risk difference. Minimum and maximum: social identity scores 4, 20; motivation scores 25, 77; knowledge scores 0, 56.

### Sensitivity analyses and robustness tests

Sensitivity tests were conducted with two alternative definitions of appropriate treatment: i) only including children with a positive malaria diagnostic test, and ii) including quinine as an appropriate treatment for malaria to account for its use as a second line treatment or during in-patient care (Table B in S1 Text). Both showed a trend of improved coverage of overall appropriate treatment (20% an 14% improvement, p values 0.006 and 0.073 respectively for i) and ii)). We performed robustness tests of the key primary and secondary impact outcomes appropriate treatment, care seeking, CHW motivation, social identity, clinical knowledge, stockouts, and attrition by repeating the analyses using a comparison of cluster-level mean outcomes (Tables D1-E2 in S1 Text). This approach estimated similar significant improvements in appropriate treatment as the random effects model with the exception of the increase in care seeking to CHWs which did not reach significance.

### Meta-analysis of the impact of the inSCALE technology intervention on appropriate treatment for MDP: Uganda and Mozambique

**Fig 2:**
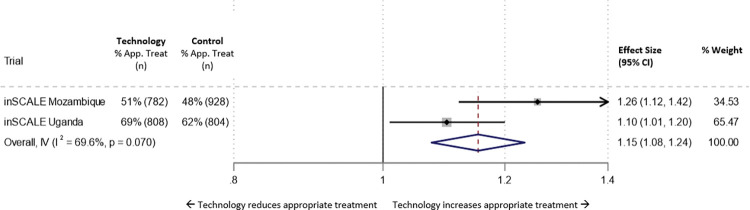
Forest plot displaying an inverse-variance weighted fixed-effect meta-analysis of the effect of the inSCALE technology interventions on appropriate treatment of episodes of suspected malaria, diarrhoea or suspected pneumonia (Uganda and Mozambique). Effect size (ES) is the risk ratio for appropriate treatment. Fixed effects meta analysis (country = fixed effect) Appr. Treat = appropriate treatment of illness episodes (suspected malaria, diarrhoea or suspecter pneumonia). I = intervention arm, C = control arm. ES = baseline-adjusted risk ratio (effect size) of % episodes MDP appropriately treated in intervention versus control arm.

Fig 2 shows the results of a fixed effects meta-analysis of the risk ratios (RRs) comparing coverage of appropriate treatment in control and intervention arms in Uganda and Mozambique. The analysis suggests implementation of the inSCALE technology model could improve appropriate treatment by 15% (8% - 24%) overall.

## Discussion

We have demonstrated that a scalable, innovative technology-based intervention implemented in Mozambique can meaningfully improve the coverage of appropriate treatment for common childhood illnesses covered by the iCCM programme by 26% (any guideline recommended treatment) and 30% (first-line treatments). These changes were mostly driven by significant improvements in the correct treatment of diarrhoea and pneumonia, and in increased appropriate treatment in primary care facilities. We did not observe between arm differences in CHW outcomes (motivation, social identity, knowledge, and attrition). We finally showed in a meta-analysis of two countries (Mozambique and Uganda) implementing similar technology-based interventions that an 8–24% improvement in appropriate treatment is likely achievable elsewhere. Our results suggest that iCCM programmes can contribute to the quality of care provided by primary health services by the use of relatively inexpensive technology that prioritises support, efficiency and performance. As a result of this work, the government of Mozambique has expanded the inSCALE technology intervention and to date seven out of eleven provinces with over 2,500 CHWs and 299 supervisors are using an expanded version of the app. The platform is being rolled out to all 8,800 CHWs and 1,300 supervisors nationally by mid-2023 and a full data integration plan with DHIS2 is ongoing to optimize strategic decision making[18, 19]. The expanded programme name, upSCALE, which includes not only the IMCI sick child module, but also growth monitoring, family planning, adult illness, HIV/TB treatment follow-up and COVID-19, reflects the successful and rapid uptake of our research into national policy and the creation of a national CHW mHealth system[19].

Our conceptual framework for the design of the inSCALE innovation expected that differences in CHW motivation, attrition and social identification would be one of the drivers of improvement in appropriate treatment[15, 20]. As noted in our formative research and in other settings, ownership of local programmes can be fragmented, CHW interventions are often only tenuously linked with the wider health system and therefore poorly supervised, and their contribution under-recognised[21]. The CHWs we surveyed however were in general highly motivated and reported a strong degree of connectedness to the wider health system in both arms at baseline and at the end of follow-up. Control arm CHWs will have received some support offered as standard practice—supervision, review of consultation records and refresher training as required. Local facility records estimated CHW attrition was moderate at 18% over the implementation period and did not differ by intervention arm. Our findings suggest that in contrast to our conceptual framework, alternative routes to impact may be more important in this setting. Though coverage of appropriate treatment in children taken to the CHW was similar across arms, we observed the largest overall increase in coverage of appropriate treatment occurred at public health facilities and in children with diarrhoea and in first line treatments for pneumonia, very similar to the pattern of impact observed in the sister inSCALE trial in Uganda [16]. That these patterns were largely consistent across two countries with separate intervention designs and implementation teams is a persuasive indicator of the alternative impact pathways and mechanisms of impact through which inSCALE operated—and warrants further exploration in future studies. This may highlight the integrated nature of inSCALE intervention where training and usage of the app by both CHWs and supervising staff at primary care facilities will have increased facility-based health worker exposure to guideline-led recommendations for disease management, alerts for low CHW stock for key commodities, motivational messaging, and reporting on prevalence of key childhood conditions. Diarrhoea in particular is often perceived simply as a symptom of a more highly prioritised condition on which treatment is focused [22] and may therefore be overlooked by health workers even if ORS is available [22–24]. These findings point to the gains that may be made by adopting an approach that not only strengthens community based iCCM programmes, but can also positively impact more broadly on the primary health care system.

Coverage of the inSCALE innovation after 18 months of follow-up was moderate to good (68% CHWs had a working phone and inSCALE app and 45% had sent one or more reports in the last month). It is difficult to assess this intervention coverage rate in context of similar interventions due to the variability in intervention components, and in endpoint definitions. A SMS (short message service) message-based randomised controlled trial in Tanzania delivered 7 messages to drug shop vendors with recommendations for correct treatment of malaria and reported 70% of dispensers received at least 75% of the intended SMS messages. Whilst this appears comparable to our reported rates, the difference in the health provider type and in the content of the intervention precludes a direct comparison [25]. Nonetheless a review of a selection of trials of child health-based mHealth interventions in low and middle income country settings (LMICs) report widely varying intervention coverage rates between 24% and 85% [26, 27].

Lack of medicinal stock for common childhood conditions has been a barrier to prompt and appropriate treatment in other settings [28], and affected our study sites in Mozambique during and after the period of implementation [8, 21]. Key issues were the national stock outs of key malaria and antibiotic medications and diagnostic tests within the last 6 months of the study, which may have supressed the full potential impact of the technology intervention for these outcomes. Research by Save the Children in this setting suggests that households who were aware of stockouts at the iCCM CHW may go elsewhere for treatment [21]. More than 50% of CHWs across both arms reporting at least one stockout of supplies for the management of MDP in the previous 3 months; such commodity supply barriers were outside the control of our intervention nonetheless it is encouraging that the intervention remained impactful in the context of these persistent issues and moderate intervention coverage.

### Wider implications for case prevalence, fatality, productivity, user costs and transmission

The highest case fatality rates for pneumonia, malaria and diarrhoea occur in sub-Saharan Africa [29, 30]; and important proximal outcomes of community-based interventions like inSCALE may include a potential to reduce community transmission of causative pathogens and/or the risk of recurrent MDP infections [31] and lowering cost of care seeking and increasing equitable access to care [24] through the strengthening and raised profile of iCCM-trained CHWs. There was a decrease in prevalence of MDP over time across the study site in Mozambique, and the additional significantly faster fall in prevalence in intervention arm districts. The primary aim of inSCALE was to deliver more appropriate treatment for MDP through a strengthening of the health system. A secondary aim of the intervention was to increase community awareness of MDP through the community-based actions of the health workers. Decreased prevalence of illness may arise through the timely use of appropriate medications, and increased awareness of preventative measures; however we cannot rule out the presence of unmeasured confounding including the influence of secular trends.

Our study had several limitations. Our estimates of prevalence of MDP and appropriate treatment were based on caregiver reports which may be subject to misclassification. Studies of caregiver recall for pneumonia and malaria symptoms have sensitivities and specificities in the range of 31%-91% depending on the setting [17, 32]. Recommended strategies to improve the accuracy of disease and treatment estimates include setting the length of the recall period to four weeks (versus the 2 week recall period used in the inSCALE baseline survey), the use of pictures of available treatments to respondents and the development of survey questions which prioritise local terms and are based on well-validated survey instruments [17, 32, 33] including the United Nations- and USAID-supported Multiple Indicator Cluster Survey and Demographic Health Surveys [34, 35]. We incorporated all these strategies in our surveys and used tailored definitions of disease and treatments in line with national guidelines, including the presumptive use of ACT to treat fever, a practice compounded by nationwide stockouts of iCCM drugs and tests at the time. Our sensitivity analyses of the main outcome with alternative definitions of appropriate treatment [see Table B in S1 Text] nonetheless produced similar results–of note, restricting cases of fever to those with a confirmed malaria test estimated a 20% improvement in appropriate treatment which remained highly significant. The inSCALE study evaluated the impact of strategies to improve scale-up and coverage of iCCM programmes using a randomised controlled design as we believed this method would provide the most convincing effectiveness estimates for stakeholders. The number of district clusters available for randomisation was however determined by iCCM programme readiness (iCCM-trained CHWs in place) and lack of concurrent interventions in the area at baseline. This resulted in twelve districts available for randomisation. We tested the robustness of our random-effects models with cluster-averaged t-tests of our primary outcomes as recommended by Hayes and Moulton [36], and whilst less powerful, we observed similar estimates of impact on appropriate treatment (See Table D1 in S1 Text). We are missing information for 495 households (250 and 245 households where no one was at home in the control and intervention arms respectively), which if we assume a similar proportion contain eligible children to the proportion observed in visited household, about half of the 495 may have contained one or more eligible children under 60 months. Any impacts on generalisability are difficult to quantify however these are likely to be minimal given the missing represents 6% of all households/eligible households.

## Conclusions

The body of research evidence for digital and mHealth programmes is increasing, however there remains a significant gap for evidence from good quality studies of the potential for available and emerging technologies to transform health outcome in LMICs for children, particularly when delivered via community-based health workers [37]. Mobile phone penetration is predicted to increase in sub-Saharan Africa [38] and is estimated to be a key medium by which health care access and service delivery can be improved for the underserved. The WHO’s recently published set of global recommendations for digital interventions for health services delivery highlight the potential of mHealth interventions for a range of uses and includes the key components of the inSCALE technology intervention–clinical decision support, stock tracking and provider-to-provider telemedicine [39]. Significantly, the 2019 WHO/UNICEF iCCM strategy meeting in Addis Ababa recommended “Interventions to improve quality, including supportive supervision and mentoring of CHWs in designated health facilities, are essential to ensure high-quality iCCM and should be budgeted for and included in district plans”[40]. This renewed encouragement for implementation of iCCM this decade may encourage relevant stakeholders looking to efficiently scale up programmes to explore digital solutions for their context. Our study and meta-analysis together with the expansion of the inSCALE programme in Mozambique [18, 19] highlight the potential scalability of an mHealth intervention aimed at strengthening iCCM systems to address the largest causes of childhood morbidity and mortality in sub-Saharan Africa.

## Materials and Methods

### Setting

The study took place in the Inhambane Province of southern Mozambique between November 2013 and July 2016. The province covers a geographical area of 68,775km^2^ with a population of approximately 1.4 million of which 17% are under 5 years of age [41]. The Inhambane mortality rate in this age group at the start of the study was 76.4 deaths per 1000 livebirths [42], with more than 60% of all deaths in the province caused by a combination of malaria, diarrhoeal diseases and pneumonia [43, 44]. Since 2011, iCCM had been included in the Inhambane programme of work for Agentes Polivalentes Elementares (APE), the name for the local cadre of community health worker and referred to as CHWs in this paper. Government and donor funding for the programme supported the training, supervision and provision of iCCM commodities for all CHWs in the province throughout the study period. A detailed study protocol for the inSCALE trial in Mozambique has been published elsewhere [15]. Details of the setting and study design for the inSCALE study in Uganda, used for the metanalysis presented in this paper, is published elsewhere [16].

### Study design and participants

We conducted a superiority open label two-arm cluster-randomised controlled trial in 12 districts in Inhambane Province in southern Mozambique. Six districts (Funhalouro, Govuro, Jangamo, Panda, Vilankulo, and Zavala) were randomised to the inSCALE intervention and six (Homoine, Inharrime, Inhassouro, Mabote, Massinga, and Morrumbene) to the control arm. The provincial and economic capitals of Inhambane: Inhambane City and Maxixe respectively, was excluded from the randomisation and Maxixe was instead used for piloting of the intervention in its rural areas during the design stage. Each district was defined as a cluster and randomly allocated to either intervention or control; the rationale being the district was the lowest unit of administration and training for the CHW iCCM programme and therefore for implementation of the inSCALE intervention. Eligible households within the district were defined as those with at least one resident child under five years of age at any point during the study in a community served by at least one iCCM-trained CHW. Local data collected by Malaria Consortium in the area in 2011 suggested that 45% of households within each community fit the eligibility criteria.

Ethical approval for the study was obtained from the Mozambique National Bioethics Committee for Health (reference 345-CNBS-10) and the London School of Hygiene & Tropical Medicine Ethics Committee in the UK (reference 5762). Approval for the random allocation of districts to intervention or control groups and implementation of the interventions was obtained from the Inhambane Provincial Office responsible for health administration for the province. Written informed consent was obtained from the main caregiver of each child under 5 years of age for baseline and follow up data collection. Consent forms included an explanation of the purpose of the data collection and confirmed participants were free to withdraw at any time. The inSCALE trial is registered on clinicaltrials.gov with identifier NCT01972321.

### Randomisation and blinding

In order to reduce the likelihood of chance imbalances between intervention arms at baseline given the number of clusters available for randomisation [36, 45]., we performed a randomisation that was restricted with respect to the percentages of households with sick children seeking care at a public health facility, the percentage seeking care to a CHW, average CHW motivation (composite) score and the log cost of care seeking for sick children. Data for these parameters was obtained from a baseline survey conducted between November- December 2012. We used the statistical software Stata (version 13) to generate the 924 possible randomisation schemes for the 12 districts and picked one at random from the subsection of 84 schemes that fitted our balance criteria [46] [see online S3 Text]. Due to the nature of the technology intervention, it was not possible to blind participants or fieldworkers to the intervention allocation, however allocation was masked in the database and all analyses were initially performed on data with intervention allocation removed and repeated on unblinded data after agreement on the final analysis methods with study partners.

### Study procedures

#### Sample size calculations

The baseline survey was conducted in all households in 2–8 randomly-selected communities (known as enumeration areas or EAs) in each district in order to cover approximately 500 households and 350 children per district (mean of 91 households per village). In the absence of site-specific data, these estimates were based on equivalent baseline data on prevalence of illness and between-cluster correlation from the sister inSCALE trial in Uganda. Following the baseline survey in Mozambique, sample size calculations for the impact analysis were updated using local data on MDP prevalence and the proportion of households with eligible children; indicating that by sampling 530 households with a rate of 48% containing eligible children we would have 90% power to detect an impact of the intervention of a 20% or higher increase in appropriate treatment given an estimated coefficient of variation in the outcome between clusters of 0.16 and a rate of 40% of children with MDP receiving appropriate treatment in the control group. Parameters and calculations are described in more detail in the inSCALE protocol paper [15]. Approximately 21 CHWs were also surveyed per district at baseline (1–2 per village). Community sampling frame data were based on complete enumeration information provided by the District Offices and EA location, size and households were verified by field workers.

#### Data collection

The baseline survey was administered to caregivers of children under 5 years of age, and collected information on symptoms of malaria, diarrhoea, and pneumonia (MDP) in these children in the last 2 weeks, care seeking for these illnesses, costs of care, socioeconomic data at the household level. Household questionnaires were based on standard health survey instruments used in the country (Mozambique DHS [34] and Malaria Indicator Cluster Surveys [35]). The CHW survey included data on CHWs’ clinical knowledge, time use, motivation and ‘social identity’–or sense and degree of connectedness to the local and national CHW and health services systems. The CHW clinical knowledge instrument was developed based on the format and example clinical event scenarios in the WHO Health Facility Survey Tool template [47]. The likert scale-based CHW motivation and identity tool used a pictorial scale indicating degree of agreement with statements about enthusiasm for the job, motivation and identification with the local and national iCCM programme, and was developed based on extensive formative research by the study team on health worker motivation and social identity [48, 49]. Both tools were adapted to the Mozambique setting prior to usage with the input of facility staff and CHWs in an iterative process that developed wording, accompanying graphics and reference to local health system structures and clinical guidelines.

Household and CHW surveys were also extensively field piloted with field staff including workshops role playing and refining of translations, and all tools were delivered in Portuguese. A quality control check of 10% of the weekly survey households was undertaken by field worker supervisors. Pictures of locally available medications were used in the household surveys to improve the accuracy of reports of treatment for MDP [17].

At the end of the follow-up period an endline impact survey was conducted in May-June 2015 in the same villages visited at baseline in households with children meeting inclusion criteria at endline, and again covering all active CHWs serving the selected villages. The survey collected data across the same themes to estimate study impact and included additional questions to CHWs and caregivers on coverage of the inSCALE innovation components in the intervention arm. The recall period for recent illness in children (caregiver reported) was extended to 4 weeks in the endline survey following the publication of a series of seminal papers recommending increasing the exposure period to 28 days to improve accuracy of reports of suspected pneumonia [17] household and CHW data collection tools are available in S2 Text.

### Intervention

The inSCALE technology intervention was developed in collaboration with Dimagi a social enterprise corporation focused on the development of digital solutions for health workers in low and middle income settings [50]. Steering and input was received from the Mozambique ministry of health, community and facility-based stakeholders, funding bodies and the inSCALE trial steering committee [15, 51]. The aim of the innovation was to leverage technological strategies to strengthen links between fragmented CHW programmes and the primary care health services including links between CHW peers and health facility staff, and by doing so to improve CHW outcomes and child health outcomes for children. The inSCALE technology intervention development process and conceptual framework linking intervention components, outputs and outcomes is described in detail in the protocol paper [15].

The technology intervention was implemented in July 2013, and after a 6 month roll-out and embedding period, follow-up to impact analysis continued to July 2015 (18 months). iCCM-trained CHWs and their health facility supervisors in the intervention arm were provided with a Samsung Galaxy smartphone installed with the inSCALE mHealth application (with CHW and facility supervisor versions), a solar charger with a number of pin adapters, and a solar lamp for night-time consultations. Intervention arm CHWs and health facility supervising staff were trained in the use of the mHealth tools. Training was conducted using a cascade approach where 7 master trainers trained 18 facility- and community-based trainers who in turn trained 47 health facility supervisors and 132 CHWs in the technology arm over 4 days with follow-up support provided by the project team during the implementation period. The inSCALE mHealth mobile app consisted of a sick child decision support algorithm in a user-friendly interface including multimedia, audio and images to improve adherence to iCCM treatment protocols. CHWs were additionally able to use the phones to track consultations with sick children, make diagnoses and management choices, report key statistics to a central server, request stock from their supervising facilities, and maintain a register of patients seen. These reports were forwarded on a weekly basis to health facility supervising staff who received tailored follow-up supervision prompts. The inSCALE app also worked offline, therefore where there were network coverage issues, CHWs and health facility supervising staff had the option to delay sending of reports until they were in a location with better coverage. CHWs and supervisors were allocated to local closed user groups which enabled complimentary calls with the inSCALE smartphones to be made without charge to fellow members in the group. Finally, CHWs and their health facility supervisors received weekly (CHWs only) and monthly (CHWs and facility supervisors) automated motivational messages tailored to their report results with the tone and emphasis based on formative work. In brief this included pretesting of messages with subgroups of CHWs and supervisors where versions of the app were evaluated on acceptability, comprehension, and usefulness, and phone functionalities were adapted accordingly. Email report formats were pretested with supervisors and iteratively updated. A series of action-oriented tips were developed based on a range of common performance-related scenarios, and all materials were adapted to Mozambique’s context and android phone software, and translated into Portuguese.

After an initial 6-month roll-out and embedding phase, the technology intervention continued to be implemented in intervention arm districts for an additional 18 months. Standard care during this period was available in both intervention and control arm districts, where CHWs and health facilities provided guideline-led management for childhood illnesses via the underlying iCCM programme and integrated management of neonatal and childhood illnesses (IMNCI) services [9, 52] respectively.

Data on CHW recruitment, training, resignations, and replacements were tracked by supervising health facilities at district level and this data was regularly extracted into the inSCALE database during the follow-up period by the study team in order to estimate attrition rates.

### Standard practice in control arm

Control arm CHWs did not receive the inSCALE package and accessories. CHWs in both arms received training in iCCM, health promotion and education, supplies of commodities including rapid diagnostic tests and drugs for common childhood illnesses, monthly supervision, and a $40 monthly subsidy. CHWs in both arms maintained paper based registers. Additional details of the components of the intervention arms is provide in Table I in S1 Text

### Outcomes

All study outcomes were based on data collected on questionnaires during the cross sectional survey at the end of the intervention follow-up period as described above (Data Collection). The primary outcome for the trial was the percentage of reported recent cases of MDP in the last 4 weeks in children 2 months to 5 years of age that were treated using appropriate medication [15]. Secondary outcomes were the prevalence of MDP in children at endline, the percentage of cases of MDP appropriately treated with recommended first line medications, the percentage of children who sought care for MDP by provider type, the percentage of CHWs who had had a stock-out of iCCM medications in the past 3 months, CHW attrition rate calculated as the proportion of CHWs who left their post during the follow-up period out of the total CHWs trained at baseline, CHW clinical knowledge scores (overall and by condition subgroup), and composite scores describing CHW enthusiasm and drive for the job (motivation) and sense of connectedness to the local and national iCCM programme (social identification). CHW knowledge scores were calculated as the sum of correct clinical decisions mentioned by the CHW in response to a series of scenarios involving children with symptoms of mild and severe disease. Points for correct items were weighted according to an algorithm defined in Tables G1-G6 in S1 Text. CHW motivation and social identification scores were calculated by a factor analysis and empirical item reduction process applied to original field tool responses. The methods have been detailed previously [53], and resulted in a three-factor CHW motivation scale of 17 items measuring general motivation, feelings of reward for effort, and retention in role. An additional single factor CHW scale of 4 items was identified which explained the majority of the variation in social identification between CHWs. Retained items from both the motivation and social identification tools showed good internal consistency (alpha = 0.67 for CHW motivation and alpha = 0.69 for CHW social identification). The final motivation scores were calculated as the product of the rotated factor analysis regression coefficients and the Likert value for each original item. The final social identification scores were calculated as the sum of the original 4 items (after reverse coding as appropriate).

Additional impact measures included the percentage of children who were appropriately treated overall whether they had symptoms of one or more of MDP in the last 4 weeks (i.e. a per-child analysis), and two sensitivity analyses of the main impact outcome using alternative definitions of appropriate treatment. All definitions of appropriate treatment and medications used in primary secondary and additional endpoints are provided in Table H in S1 Text.

### Statistical analysis

All analyses were based on intention-to-treat principles and accounted for the cluster-randomised design. Multivariate mixed-effects models were specified for all impact outcomes, which account for the clustering of outcomes by inclusion of a random intercept at the district level. Intervention arm, the cluster mean percentage of children who were appropriately treated at baseline, and the parameters used to balance intervention arms during randomisation were included as fixed effects. Unadjusted analyses of impact were also performed, and the results included in Table A in S1 Text. For the primary outcome and other binary outcomes, odds ratios were converted to risk ratios using the marginal standardisation technique and 95% CIs estimated using the delta method [54]. Additional sensitivity analyses of the primary outcome were conducted to account for alternative treatments available for MDP [see Table H in S1 Text for definitions]. Robustness checks of the primary outcomes were conducted by estimating cluster averaged results using t-tests following the procedures of Hayes and Moulton [36], recommended when the number of clusters per arm is less than 10[36] [see Tables D1-E2 in S1 Text]. CHW attrition was estimated using cluster-averaged mean rates as human resources information was provided at this level only from district health offices. In order to estimate a pooled effect of the inSCALE technology intervention on appropriate treatment across both Uganda and Mozambique, we performed a meta-analysis of effect sizes by combining the results of the Mozambique (this study) and Uganda [16] trials of the inSCALE intervention. As there was no significant heterogeneity (<50% estimated from exploratory meta-analysis models) between studies, we present the pooled result from a fixed effects meta-analysis.

## Supporting information

S1 Text Table A. Unadjusted analyses of primary outcomes. Table B. Sensitivity analyses for primary outcome at endline (any episode of MDP): Alternative definitions of appropriate malaria treatment. Table C. Whole Child Analysis–Appropriate treatment. Table D1. Robustness tests (cluster-averaged outcomes)–Primary Outcomes. Table D2. Robustness tests (cluster-averaged outcomes)–Secondary Outcomes: appropriate treatment stratified by condition or provider Table E1. Robustness tests (cluster-averaged outcomes)–Secondary outcomes: prevalence of suspected and confirmed malaria, malaria blood testing, diarrhoea, and pneumonia. Table E2. Robustness tests (cluster-averaged outcomes)–Secondary outcomes: prevalence of care-seeking for suspected malaria, diarrhoea and pneumonia. Table F. CHW clinical knowledge by subgroup (diagnosis of MDP, treatment of MDP). Table G1. Item weighting–Case study: Francis/Alberte (pneumonia and diarrhoea). Table G2. Item weighting–Case study: Hope/Tina (malaria). Table G3. Item weighting–Case study: Beatrice/Janete (severe pneumonia). Table G4. Item weighting–Case study: Muteesa/Kizito (severe disease/malaria). Table G5. Item weighting–Case study: James (severe disease/malaria). Table G6. CHW Knowledge scoring for subcategories: diagnosis and treatment of suspected malaria, diarrhoea and pneumonia. Table H. Definitions of appropriate treatment and other key child health outcomes. Table I. **Intervention and control arm components—inSCALE Mozambique.**
(DOCX)

S2 Text inSCALE data collection forms. (DOCX)

S3 Text Restricted Randomisation Procedure and codes–inSCALE Mozambique. (DOCX)
