# Supplementary material for: Improving outcomes for children with malaria, diarrhoea and pneumonia in Mozambique: A cluster randomised controlled trial of the inSCALE technology innovation
Source: PLOS Digit Health. 2023 Jun 12;2(6):e0000235. doi: 10.1371/journal.pdig.0000235 (PMC10260254; doi:10.1371/journal.pdig.0000235)
Supplement: S1 Text — Table B. Sensitivity analyses for primary outcome at endline (any episode of MDP): Alternative definitions of appropriate malaria treatment. Table C. Whole Child Analysis–Appropriate treatment. Table D1. Robustness tests (cluster-averaged outcomes)–Primary Outcomes. Table D2. Robustness tests (cluster-averaged outcomes)–Secondary Outcomes: appropriate treatment stratified by condition or provider Table E1. Robustness tests (cluster-averaged outcomes)–Secondary outcomes: prevalence of suspected and confirmed malaria, malaria blood testing, diarrhoea, and pneumonia. Table E2. Robustness tests (cluster-averaged outcomes)–Secondary outcomes: prevalence of care-seeking for suspected malaria, diarrhoea and pneumonia. Table F. CHW clinical knowledge by subgroup (diagnosis of MDP, treatment of MDP). Table G1. Item weighting–Case study: Francis/Alberte (pneumonia and diarrhoea). Table G2. Item weighting–Case study: Hope/Tina (malaria). Table G3. Item weighting–Case study: Beatrice/Janete (severe pneumonia). Table G4. Item weighting–Case study: Muteesa/Kizito (severe disease/malaria). Table G5. Item weighting–Case study: James (severe disease/malaria). Table G6. CHW Knowledge scoring for subcategories: diagnosis and treatment of suspected malaria, diarrhoea and pneumonia. Table H. Definitions of appropriate treatment and other key child health outcomes. Table I. Intervention and control arm components—inSCALE Mozambique. (DOCX) [file pdig.0000235.s001.docx]

**S1 Text: additional analyses.**

**Improving community health worker treatment for malaria, diarrhoea and pneumonia in Mozambique through the inSCALE mHealth innovation: A cluster randomised controlled trial**

Seyi Soremekun, Karin Källander, Raghu Lingam, Ana-Cristina Castel Branco, Neha Batura, Daniel Strachan, Abel Muiambo, Nelson Salomao, Juliao Condoane, Fenias Benhane, Frida Kasteng, Anna Vassall, Zelee Hill, Guus ten Asbroek, Sylvia Meek, James Tibenderana; and Betty Kirkwood

**Table A Unadjusted analyses of primary outcomes**

| **Outcome** | **Control** | | **Technology** | | **Technology v Control** | |
| --- | --- | --- | --- | --- | --- | --- |
|  | **%** | **n/N*** | **%** | **n/N*** | **Effect estimate (95% CI)** | **p** |
| Appropriate treatment for MDP | 47.8% | 928/1941 | 50.7% | 782/1543 | RR: **1.11** (0.75 - 1.64) | 0.594 |
| Appropriate treatment for MDP (first line treatments only) | 42.2% | 819/1941 | 48.2% | 744/1543 | RR: **1.19** (0.78 - 1.82) | 0.425 |
| Care seeking to the CHW (first port of call) | 14.4% | 211/1467 | 15.9% | 199/1251 | RR: **1.20** (0.56-2.57) | 0.631 |
| % CHWs experienced a stockout of any MDP drug in last 3 months* | 52.4% | 54/103 | 56.7% | 59/104 | RR: **1.10** (0.74 – 1.63) | 0.640 |
| Mean CHW motivation score | 4.6 (SD 0.5) | - | 4.7 (SD 0.5) | - | RD: **-0.01** (-0.51 – 0.48) | 0.954 |
| Mean CHW social identity score | 4.7 (SD 0.6) | - | 4.8 (SD 0.5) | - | RD: **0.03** (-0.35 – 0.41) | 0.860 |
| Mean CHW knowledge score | 19.7 (SD 4.3) | - | 19.8 (SD 4.4) | - | RD: **0.56** (-2.04 – 3.15) | 0.675 |
| CHW attrition (%) | 18.4% (SD 13.6) | - | 18.5% (SD 10.0) | - | RD: **0.11** (-15.23, 15.45) | 0.987 |

* Stockouts of any of artemisinin combination therapy, amoxycillin, or ORS. RR = risk ratio; RD = risk difference

**Table B Sensitivity analyses for primary outcome at endline (any episode of MDP): Alternative definitions of appropriate malaria treatment**

| **Primary Impact Outcome (Appropriate treatment): Sensitivity definition** | **Control** | | **Technology** | | **Technology v Control** | |
| --- | --- | --- | --- | --- | --- | --- |
|  | **%** | **n/N*** | **%** | **n/N*** | **RR (95% CI)** | **p** |
| Appropriate treatment for episodes of MDP (where malaria episodes restricted to test confirmed malaria only) | 65.9% | 858/1302 | 71.2% | 738/1037 | **1.20** (1.05-1.37) | 0.006 |
| Appropriate treatment includes quinine for suspected malaria episodes (adjusted for baseline) | 48.3% | 938/1941 | 50.9% | 786/1543 | **1.14** (0.99-1.32) | 0.073 |

Random effects model (cluster = random effect) including baseline parameters: percentage of care seeking to the CHW, to a health facility, CHW factor motivation score and the log cost of seeking care, all at cluster level.

* N is the total number of children >2 months of age (diarrhoea and pneumonia) and >4 months of age (malaria) with MDP

**Table C Whole Child Analysis – Appropriate treatment**

The whole child analysis is the proportion of children who received appropriate treatment for all the conditions they presented with in the last 4 weeks (i.e. one or more of suspected malaria, diarrhoea and/or suspected pneumonia)

| **Outcome** | **Control** | **Technology** | |
| --- | --- | --- | --- |
|  | **% (N)** | **% (N)** | **Versus Control**  **RR (95% CI) P** |
| Appropriate treatment | 41.45 (608) | 48.04 (601) | **1.40** (1.09-1.79) p=0.009 |
| Appropriate treatment (ORS + Zinc for diarrhoea) | 39.13 (574) | 45.56 (570) | **1.40** (1.12-1.76) p=0.003 |
| Appropriate treatment (first line drugs only) | 36.13 (530) | 46.28 (579) | **1.54** (1.20-1.98) p=0.001 |

Robustness checks.

Comparison of the mean of the cluster level mean values for key outcomes as recommended in Hayes and Moulton 2017 for studies with <10 clusters per arm (38). Models were adjusted for cluster-level means of variables used to restrict the randomisation (care-seeking to the CHW and to public health facilities, CHWV motivation scores and mean log cost of care-seeking for families) and for the cluster-averaged baseline level of the outcome in question. Note CHW attrition data was only available at cluster level, therefore model output is identical to the result in the main paper.

**Table D1 Robustness tests (cluster-averaged outcomes) – Primary Outcomes**

| **Outcome** | **Control** | **Technology** | | |
| --- | --- | --- | --- | --- |
|  | **Mean % coverage (SD)** | **Mean % coverage (SD)** | **RR (95% CI)** | **p** |
| Appropriate treatment (any of MDP) | 44.6% (17.2) | 48.9% (15.4) | **1.26** (1.01 – 1.57) | 0.0412 |
| Appropriate treatment - first line treatments (any of MDP) | 39.7% (15.6) | 46.4% (16.8) | **1.30** (0.99 – 1.72) | 0.0581 |
| Care seeking to the CHW (first port of call) | 15.6% (12.4) | 15.6% (6.5) | **1.58** (0.67 – 3.75) | 0.2314 |
| % CHWs experienced a stockout of any MDP drug in last 3 months* | 53.01 (16.0) | 58.31 (22.7) | **1.12** (0.93 – 1.37) | 0.1823 |
| **Outcome** | **Mean score (SD)** | **Mean score (SD)** | **Score difference (95% CI)** | **p** |
| CHW attrition (cluster percentage) | 18.4% (13.6) | 18.5% (10.0) | **+1.31** (-18.66, 21.30) | 0.878 |
| APE motivation (mean factor score) | 4.62 (0.41) | 4.60 (0.46) | **-0.03** (-0.71 – 0.64) | 0.907 |
| APE identity (mean factor score) | 4.72 (0.34) | 4.74 (0.33) | **-0.00** (-0.53 – 0.53) | 0.998 |
| CHW knowledge score | 19.65 (4.31) | 19.81 (4.39) | **1.03** (-2.86 – 4.93) | 0.525 |

**Table D2 Robustness tests (cluster-averaged outcomes) – Secondary Outcomes: appropriate treatment stratified by condition or provider**

| **Outcome** | **Control** | **Technology** | | |
| --- | --- | --- | --- | --- |
|  | **Mean % coverage (SD)** | **Mean % coverage (SD)** | **RR (95% CI)** | **p** |
| **By condition** | | | | |
| Suspected malaria | 54.4% (707/1300) | 56.6% (633/1118) | **1.06** (0.88-1.27) | 0.4606 |
| Confirmed malaria | 96.4% (637/661) | 96.2% (589/612) | **1.00** (0.94-1.08) | 0.7506 |
| Diarrhoea (ORS) | 29.3% (58/198) | 43.8% (53/121) | **1.92** (1.02-3.63) | 0.0465 |
| Diarrhoea (ORS + zinc) | 0.0% (0/198) | 1.7% (2/121) | - | 0.1823 |
| Suspected pneumonia (any antibiotic) | 36.8% (163/443) | 31.6% (96/304) | **0.88** (0.61-1.26) | 0.4033 |
| Suspected pneumonia (amoxycillin) | 12.2% (54/443) | 19.1% (58/304) | **1.51** (1.00-2.27) | 0.0475 |
| **By provider (cases of MDP)** | | | | |
| CHW | 49.5% (146/ 295) | 61.9% (148/239) | **1.09** (0.77-1.56) | 0.646 |
| Public Facility | 58.0% (736/1269) | 62.2% (594/955) | **1.37** (0.90-2.09) | 0.1115 |
| Private sector (private facility, pharmacy, shop, herbalist) | 29.4% (10/34) | 21.7% (5/23) | **1.06** (0.51-2.18) | 0.8533 |
| No care seeking outside of the home | 10.5% (36/343) | 10.7% (35/326) | **1.09** (0.54-2.22) | 0.7561 |

**Table E1 Robustness tests (cluster-averaged outcomes) – Secondary outcomes: prevalence of suspected and confirmed malaria, malaria blood testing, diarrhoea, and pneumonia**

| **Outcome** | **Control arm % (n/N)** | **Technology arm % (n/N)** | **Impact of inSCALE at endline**  **Risk ratio inSCALE/control**  **RR: 95% CI; p***** |
| --- | --- | --- | --- |
|  |  |  |  |
| Prevalence any of MDP | 53.5% (1467/2740) | 43.7% (1251/2863) | **0.80** (0.70-0.92) p=0.0066 |
| Prevalence suspected malaria* | 48.5% (1300/2682) | 39.8% (1118/2808) | **0.80** (0.69-0.94) p=0.0156 |
| Prevalence blood test for malaria | 59.7% (937/1570) | 62.3% (831/1335) | **1.16** (1.06-1.28) p=0.002 |
| Prevalence confirmed malaria** | 70.5% (661/937) | 73.7% (612/831) | **1.07** (0.85-1.35) p=0.4794 |
| Prevalence diarrhoea | 7.2% (198/2740) | 4.2% (121/2863) | **0.56** (0.37-0.83) p=0.0130 |
| Prevalence pneumonia | 16.2% (443/2740) | 10.6% (304/2863) | **0.77** (0.42-1.41) p=0.2955 |

**Table E2 Robustness tests (cluster-averaged outcomes) – Secondary outcomes: prevalence of care-seeking for suspected malaria, diarrhoea and pneumonia**

| **Outcome** | **Control (N=1467)** | **Technology (N=1251)** | **Impact of inSCALE at endline**  **Risk ratio inSCALE/control**  **RR: 95% CI; p***** |
| --- | --- | --- | --- |
|  |  |  |  |
| % seeking care at a CHW (at any point) | 14.7% (215) | 16.2% (203) | **1.56** (0.67-3.79) p=0.2552 |
| % Not seeking care outside the home | 18.3% (269) | 20.6% (251) | **0.91** (0.59-1.40) p=0.6032 |
| % seeking care at a public facility (at any point) | 67.4% (988) | 63.7% (797) | **1.14** (0.86-1.53) p=0.2822 |
| % seeking care in the private sector (at any point - private facility, pharmacy, shop, herbalist etc) | 3.6% (53) | 2.4% (30) | **0.70** (0.23-2.15) p=0. 0.4630 |

Prevalence (n/N) calculated as total eligible children 2 months-59 months of age (N) with condition in question (n). Note age range for fever and malaria is 4 months-59 months in line with treatment guidelines. *Fevers, excludes blood test negative fevers. **percentage of all blood tested fevers that were positive. *** Adjusted for i) parameters used to balance arms at baseline and ii) the baseline prevalence of prevalence parameter

**Table F. CHW clinical knowledge by subgroup (diagnosis of MDP, treatment of MDP)**

| **Condition** | **Control arm (103)** | **Technology arm (104)** | **risk ratio versus control (95% CI)** | **p** |
| --- | --- | --- | --- | --- |
|  | **% (n)** | **% (n)** |  |  |
| Identification and diagnosis of suspected malaria* | 97.09% (100) | 99.04% (103) | 0.86 (0.00-4.08*10^10^) | 0.990 |
| Treatment of malaria | 97.09% (100) | 98.08% (102) | 1.07 (0.92-1.23) | 0.386 |
| Identification and diagnosis of diarrhoea | 20.39% (21) | 25.00% (26) | 1.38 (0.77-2.48) | 0.283 |
| Treatment of diarrhoea (ORS) | 46.60% (48) | 44.23% (46) | 1.00 (0.74-1.35) | 0.988 |
| Treatment of diarrhoea (ORS + zinc) | 29.13% (30) | 28.85% (30) | 1.11 (0.65-1.88) | 0.702 |
| Identification and diagnosis of suspected pneumonia | 66.02% (68) | 73.08% (28) | 1.16 (0.96-1.41) | 0.131 |
| Treatment of pneumonia | 62.14% (64) | 61.54% (64) | 1.03 (0.79-1.35) | 0.812 |

**inSCALE CHW Clinical Knowledge: Instrument scoring**

The inSCALE CHW Performance assessment tool was based on the WHO Health Worker Performance Assessment Instrument: Case Scenarios (49). The performance of CHWs in treating malaria, diarrhoea and pneumonia both in mild and severe forms were tested by an inSCALE interviewer using theoretical scenarios involving sick children. For every unprompted correct action mentioned in response, the interviewer marked the corresponding item on the checklist (see data collection forms).

**Table G1. Item weighting – Case study: Francis/Alberte (pneumonia and diarrhoea)**

| item | Score weight | item | Score weight | item | Score weight |
| --- | --- | --- | --- | --- | --- |
| Asks about the duration of child’s cough | 1 | Prescribes antibiotics; amoxicillin | 1 | Arranges a follow-up visit | 0.5 |
| Asks if child had fever | 0.5 | Prescribes zinc tablets | 1 | Records visit in VHT registration book | 0.5 |
| Asks if any blood in child’s stool | 0.5 | Advises dose of AL/Coartem – 3 days | 0 | Advises continued feeding and fluids for child | 0.5 |
| Asks if child has been vomiting | 0.5 | Advises dose of ORS – as often as needed | 1 | Advises to return or take child to facility if worsens | 0.5 |
| Asks if child had convulsions | 0.5 | Gives first dose of amoxicillin (or cotrimoxazole – Mozambique) | 1 | Checks child health or vaccination record | 0.5 |
| Ask about the duration of child’s diarrhoea | 1 | Gives first dose of ORS | 1 |  |  |
| Asks about the duration of child’s fever | 0 | Gives first dose of zinc | 1 |  |  |
| Asks/checks whether child can drink/breastfeed | 0.5 | Prescribes anti-malarial; Coartem | 0 |  |  |
| Asks/checks if child is very sleepy/unconsciousness | 0.5 | Prescribes ORS | 1 |  |  |
| Asks if child had any other problem | 0.5 | Advises dose of amoxicillin – 5 days (or cotrimoxazole – Mozambique) | 1 |  |  |
| Counts breaths of child | 1 | Advises dose of zinc – 10 days | 1 |  |  |
| Performs an RDT (blood test for malaria) on child | 0 | Gives first dose of AL/Coartem | 0 |  |  |
| Checks for chest indrawing | 1 | Gives dose of rectal artesunate/plasmotrim | 0 |  |  |
| Identifies the child as having fast breathing/pneumonia | 1 | Child does not need any treatment from VHT | 0 |  |  |
| Identifies the child as having danger sign/referral symptom or severe illness (any) | 0 | Refers to health facility | 0 |  |  |
| Identifies the child as having malaria | 0 | Writes referral note | 0 |  |  |
| Identifies the child as having diarrhoea | 1 | Advises use of ITN for child | 0.5 |  |  |

**Table G2. Item weighting – Case study: Hope/Tina (malaria)**

| item | Score weight | item | Score weight | item | Score weight |
| --- | --- | --- | --- | --- | --- |
| Asks about the duration of child’s fever | 1 | Advises dose of AL/Coartem – 3 days | 1 | Advises continued feeding and fluids for child | 0.5 |
| Asks if the child has a cough | 0.5 | Advises dose of ORS – as often as needed | 0 | Advises to return or take child to facility if worsens | 0.5 |
| Asks if child had convulsions | 0.5 | Gives first dose of amoxicillin | 0 | Checks child health or vaccination record | 0.5 |
| Asks if child had any other problem | 0.5 | Gives first dose of ORS | 0 |  |  |
| Asks if the child has diarrhoea | 0.5 | Gives first dose of zinc | 0 |  |  |
| Asks if child has been vomiting | 0.5 | Prescribes anti-malarial; Coartem | 1 |  |  |
| Asks/checks whether child can drink/feed | 0.5 | Prescribes ORS | 0 |  |  |
| Asks/checks if child is very sleepy/unconsciousness | 0.5 | Advises dose of amoxicillin – 5 days | 0 |  |  |
| Counts breaths of child | 0 | Advises dose of zinc – 10 days | 0 |  |  |
| Performs an RDT (blood test for malaria) on child | 1 | Gives first dose of AL/Coartem | 1 |  |  |
| Checks for chest indrawing in the child | 0 | Gives dose of rectal artesunate/plasmotrim | 0 |  |  |
| Identifies the child as having fast breathing/pneumonia | 0 | Child does not need any treatment from VHT | 0 |  |  |
| Identifies the child as having danger sign/referral symptom or severe illness (any) | 0 | Refers to health facility | 0.5 |  |  |
| Identifies the child as having malaria | 1 | Writes referral note | 0.5 |  |  |
| Identifies the child as having diarrhoea | 0 | Advises use of ITN for child | 0.5 |  |  |
| Prescribes antibiotics; amoxicillin | 0 | Arranges a follow-up visit | 0.5 |  |  |
| Prescribes zinc tablets | 0 | Records visit in VHT registration book | 0.5 |  |  |

**Table G3 Item weighting – Case study: Beatrice/Janete (severe pneumonia)**

| item | Score weight | item | Score weight | item | Score weight |
| --- | --- | --- | --- | --- | --- |
| Identifies the child as having fast breathing/pneumonia | 1 | Prescribes anti-malarial; Coartem | 0 | Advises continued feeding and fluids for child | 0.5 |
| Identifies the child as having danger sign/referral symptom or severe illness (any) | 1 | Prescribes ORS | 0 | Advises to return or take child to facility if worsens | 0 |
| Identifies the child as having malaria/diarrhoea | 0 | Advises dose of amoxicillin – 5 days | 0 | Checks child health or vaccination record | 0.5 |
| Identifies the child has no major problem | 0 | Advises dose of zinc – 10 days | 0 |  |  |
| Prescribes antibiotics; amoxicillin | 1 | Gives first dose of AL/Coartem | 0 |  |  |
| Prescribes zinc tablets | 0 | Gives dose of rectal artesunate/plasmotrim | 0 |  |  |
| Advises dose of AL/ Coartem – 3 days | 0 | Child does not need any treatment from VHT | 0 |  |  |
| Advises dose of ORS – as often as needed | 0 | Refers to health facility | 1 |  |  |
| Gives first dose of amoxicillin (or cotrimoxazole – Mozambique) | 1 | Writes referral note | 0.5 |  |  |
| Gives first dose of ORS | 0 | Advises use of ITN for child | 0.5 |  |  |
| Gives first dose of zinc | 0 | Arranges a follow-up visit | 0.5 |  |  |

**Table G4. Item weighting – Case study: Muteesa/Kizito (severe disease/malaria)**

| item | Score weight | item | Score weight | item | Score weight |
| --- | --- | --- | --- | --- | --- |
| Identifies the child as having fast breathing/pneumonia | 0 | Prescribes anti-malarial; Coartem | 0 | Advises continued feeding and fluids for child | 0.5 |
| Identifies the child as having danger sign/referral symptom or severe illness (any) | 1 | Prescribes ORS | 0 | Advises to return or take child to facility if worsens | 0 |
| Identifies the child as having malaria | 0.5 | Advises dose of amoxicillin – 5 days | 0 | Checks child health or vaccination record | 0.5 |
| Identifies the child as having diarrhoea | 1 | Advises dose of zinc – 10 days | 0 | Advises use of ITN for child | 0.5 |
| Prescribes antibiotics; amoxicillin | 0 | Gives first dose of AL/Coartem | 0 | Arranges a follow-up visit | 0.5 |
| Prescribes zinc tablets | 0 | Gives dose of rectal artesunate/plasmotrim | 1 |  |  |
| Advises dose of AL/ Coartem – 3 days | 0 | Child does not need any treatment from VHT | 0 |  |  |
| Advises dose of ORS – as often as needed | 0 | Refers to health facility | 1 |  |  |
| Gives first dose of amoxicillin | 0 | Writes referral note | 0.5 |  |  |

**Table G5. Item weighting – Case study: James (severe disease/malaria)**

| item | Score weight | item | Score weight | item | Score weight |
| --- | --- | --- | --- | --- | --- |
| Identifies the child as **NOT** having fast breathing/pneumonia | 1 | Prescribes anti-malarial; Coartem | 0 | Advises continued feeding and fluids for child | 0.5 |
| Identifies the child as **NOT** having danger sign/referral symptom or severe illness (any) | 1 | Prescribes ORS | 0 | Advises to return or take child to facility if worsens | 0.5 |
| Identifies the child as **NOT** having malaria/diarrhoea | 1 | Advises dose of amoxicillin – 5 days | 0 | Checks child health or vaccination record | 0.5 |
| Identifies the child has no major problem | 1 | Advises dose of zinc – 10 days | 0 | Advises use of ITN for child | 0.5 |
| Prescribes antibiotics; amoxicillin | 0 | Gives first dose of AL/Coartem | 0 | Arranges a follow-up visit | 0.5 |
| Prescribes zinc tablets | 0 | Gives dose of rectal artesunate/plasmotrim | 0 |  |  |
| Advises dose of AL/ Coartem – 3 days | 0 | Child does not need any treatment from VHT | 0 |  |  |
| Advises dose of ORS – as often as needed | 0 | Refers to health facility | 0 |  |  |
| Gives first dose of amoxicillin | 0 | Writes referral note | 0 |  |  |

**Table G6. CHW Knowledge scoring for subcategories: diagnosis and treatment of suspected malaria, diarrhoea and pneumonia**

| **subcategory** | **Knowledge item awarded if below items mentioned in response** |
| --- | --- |
| Identification and diagnosis of suspected malaria (scenario Table S1b) | Identifies the child as having malaria |
| Treatment of malaria (scenario Table S1b) | Recommends one or more of:  Prescribes anti-malarial; Coartem  Gives first dose of AL/Coartem  Advises dose of AL/Coartem – 3 days  Gives dose of rectal artesunate/plasmotrim if severe |
| Identification and diagnosis of diarrhoea (scenario Table S1a) | Identifies the child as having diarrhoea |
| Treatment of diarrhoea (scenario Table S1a) | For outcome “ORS only”; Recommends one or more of:   - Prescribes ORS - Gives first dose of ORS - Advises dose of ORS – as often as needed   For outcome “ORS+zinc” must also recommend one or more of:   - Gives first dose of zinc - Prescribes zinc tablets - Advises dose of zinc – 10 days |
| Identification and diagnosis of suspected pneumonia (scenario Table S1a) | Identifies the child as having fast breathing/pneumonia |
| Treatment of pneumonia (scenario Table S1a) | Recommends one or more of:   - Prescribes antibiotics; amoxicillin - Gives first dose of amoxicillin (or cotrimoxazole – Mozambique) - Advises dose of amoxicillin – 5 days (or cotrimoxazole – Mozambique) |

**Table H: Definitions of appropriate treatment and other key child health outcomes**

| Illness condition | Illness definition | Appropriate treatment (first line treatments in bold) |
| --- | --- | --- |
| Suspected malaria | Reported fever in the previous four weeks, excluding those confirmed through a blood test as not having malaria. | **Any Artemisinin combination Therapy**: Coartem, Duo-Cotecxin, Amodiaquin-Artesunate, or Artesunate-Fansidar. Rectal Artesunate if malaria + danger sign. Any ACT or Quinine will be analysed as a secondary outcome. |
| Confirmed malaria | Reported fever in the previous four weeks, restricted to those with a positive blood test for malaria |  |
| Diarrhoea | Reported diarrhoea in the previous 4 weeks, specifically three or more watery stools in a 24hr period. | **Oral rehydration salts** (homemade or ready-made). ORS plus zinc supplementation will be analysed as a secondary outcome. |
| Suspected pneumonia (“pneumonia” in this report) | Reported cough with fast/difficult breathing (which was not due to a blocked nose), or chest indrawing | Amoxicillin, Chloramphenicol, Erythromycin, Ceftriaxone, Azythromycin, Cephalexin, or Cotrimoxazole (baseline only) |
| Episode of any of suspected malaria, diarrhoea or suspected pneumonia | Each episode where one of the above sets of signs occurs is viewed as a separate case |  |

**Table I: Intervention and control arm components - inSCALE Mozambique**

|  | **Control arm** | **mHealth arm** |
| --- | --- | --- |
| **Training** | - Basic training in health promotion and health education - Training in iCCM | - Basic training in health promotion and health education - Training in iCCM - CHWs and supervisors trained in ‘inSCALE CommCare application’ to assess, treat and refer sick children, submit weekly patient and stock data and receive motivational feedback messages. |
| **Accessories and materials** | - Supply of commodities | - Supply of commodities - Samsung smartphone (Android) - Solar lamp (Sun King Pro) with multiple phone charging pins - Job aids |
| **Supervision and support** | - Monthly and quarterly supervision (though in reality it was less frequent and irregular) | Monthly and quarterly supervision (Monthly and quarterly supervision (though in reality it was less frequent and irregular) Automated SMS sent to supervisors flagging problems and strengths identified in the data submitted, and alerting supervisors about CHWs requiring targeted supervision.CHW supervisors trained in effective supervision skills using electronic core competency assessment tools and as trainers of CHW in the mHealth intervention.  - Closed user groups for free calls between CHWs and with their supervisors |
| **Reporting tools** | - Paper based CHW register - Monthly paper-based aggregated reports | - Paper based CHW register - Monthly paper-based aggregated reports - ‘inSCALE CHW CommCare app’ to send aggregated weekly reports on patients seen (sex, mRDT results, symptoms and classification of signs, treatment given and outcome of treatment) and current drug stock levels. |
| **CHW motivation** | - Monthly subsidy (approximately US$40) | - Monthly subsidy (approximately US$40) - Relevant and personalised weekly feedback messages based on submitted data sent by SMS. |

Restricted Randomisation Procedure and codes – inSCALE Mozambique

**1. Rationale for restricted randomisation**

Restricted randomisation aims to balance intervention arms on key covariates related to the primary outcome. An alternative is simple (non-restricted), randomisation which poses an increased risk of chance imbalance between arms for key variables particularly in trials with a relatively small number of clusters (Moulton 2004). The primary study outcome was appropriate treatment of major childhood illness (diarrhoea, malaria and pneumonia). Restricted randomisation was based on indicators available at baseline thought to vary proportionally to appropriate treatment: percentages of households with sick children seeking care at a public health facility, percentage seeking care to an CHW, CHW motivation (composite) score and cost of care seeking at baseline.

**2. Data sources for indicators used in the restricted randomisation and procedure**

Data on restriction variables was collected for all 12 districts (clusters) from a sample of households and from all CHWs during a baseline survey in November-December 2012.

There are 924 possible unique ways (12! ÷ (6!*6!)) to sort 12 districts into a control group (group 1) and intervention group (group 2);the scheme to be picked at random would be from a sub-sample of these 924 where there was a good balance between the arms for:

1. CHW motivation score – no more than a **0.5 difference** between arms
2. Cost (log10) of care seeking – no more than a **0.1 difference** between arms
3. % care seeking to an CHW – no more than an **3.5% difference** between arms
4. % care seeking to a public facility – no more than a **5% difference** between arms

**Iterative random sorting:** In an iterative process, the clusters were sorted randomly into two groups and repeated- up to 100,000 times; 84 unique allocation schemes which met all balance criteria were saved.

**3. FINAL RANDOMISATION SCHEME**

**84/924 schemes fit all the above criteria.**

A scheme was picked at random from the final 84 schemes (seed=05042013, the date of the randomisation) as the final scheme for the Mozambique study (Table 1):

| **Control** | **Technology** |
| --- | --- |
| Funhalouro | Homoine |
| Govuro | Inharrime |
| Jangamo | Inhassouro |
| Panda | Mabote |
| Vilankulo | Massinga |
| Zavala | Morrumbene |

**Table S9: Final randomisation scheme for the inSCALE intervention in Mozambique 2013**

**Randomisation Procedure Code (Stata v 13)**

**********

*INSCALE Randomisation procedure Mozambique 2013: Restricted on 4 variables:

*facility care seeking rate, APE (CHW) care seeking rate, log cost of care seeking for households, and CHW motivation score

*March 2013, updated May 2013 and annotated Dec 2021

*SSoremekun

*NOTES*

*Using Cluster_m database (all cluster level summary vars used in randomisation)

*This do file creates a file containing all allocation schemes meeting criteria; using different balance criteria limits: allowed_tm1/...tm12

*final agreed set of balance criteria saved in file: allowed_tm10

**********

clear all

cd D:\inscale\mozambique

************************

*Part I - randomisation and restrictions

file open fh using allowed_tm10.txt, write replace

file write fh "allocation" _tab "ds1" _tab "ds2" _tab "ds3" _tab "ds4" _tab "ds5" _tab "ds6" _tab "ds7" _tab "ds8" ///

_tab "ds9" _tab "ds10" _tab "ds11" _tab "ds12"

forvalues i=1/100000 {

use cluster_m, clear

rename district ds

set seed `i'

generate random = runiform()

sort random

generate group = group(2)

tabstat facill apeill clusmotf cluslogcost if group==1, stat(mean) save

matrix stats=r(StatTotal)

tabstat facill apeill clusmotf cluslogcost if group==2, stat(mean) save

matrix stats2=r(StatTotal)

if stats[1, 1]-stats2[1, 1]>=-5 & stats[1, 1]-stats2[1, 1]<=5 & ///

stats[1, 2]-stats2[1, 2]>=-2.5 & stats[1, 2]-stats2[1, 2]<=2.5 & ///

stats[1, 3]-stats2[1, 3]>=-0.5 & stats[1, 3]-stats2[1, 3]<=0.5 & ///

stats[1, 4]-stats2[1, 4]>=-0.2 & stats[1, 4]-stats2[1, 4]<=0.2 {

*randomisation scheme long--> wide and into text file:

sort group ds

keep ds

gen number=_n

gen all=`i'

reshape wide ds, i(all) j(number)

file write fh _n (all) _tab (ds1) _tab (ds2) _tab (ds3) _tab (ds4) _tab (ds5) _tab (ds6) _tab (ds7) _tab (ds8) ///

_tab (ds9) _tab (ds10) _tab (ds11) _tab (ds12)

}

else {

continue

}

}

file close fh

insheet using allowed_tm10.txt,clear

// identical schemes sorted to be together + duplicates dropped

sort ds1-ds12

compress

gen dup=1 if ds1==ds1[_n+1] & ds2==ds2[_n+1] & ds3==ds3[_n+1] & ds4==ds4[_n+1] & ds5==ds5[_n+1] & ds6==ds6[_n+1] ///

& ds7==ds7[_n+1] & ds8==ds8[_n+1] & ds9==ds9[_n+1] & ds10==ds10[_n+1] & ds11==ds11[_n+1] & ds12==ds12[_n+1]

drop if dup==1

browse

save allowed_tm10, replace

!"C:\Users\seyi\AppData\Local\Google\Chrome\Application\chrome.exe" "http://www.youtube.com/watch?v=-_kwXNVCaxY"

stop

*For reference: e.g. of other notification sound files on machine:

!"C:\Program Files\Windows Media Player\wmplayer" "C:\Windows\Media\Raga\Windows Exclamation.wav"

!"C:\Program Files\Windows Media Player\wmplayer" C:\Windows\Media\tada.wav

**********************

*PART II: Check for bias in schemes meeting balance criteria

clear all

cd D:\inscale\mozambique

use allowed_tm10

file open bs using bias_m10.txt, write replace

file write bs "distr_1" _tab "distr_2" _tab "freq" _tab "arm"

local listall Funhalouro Govuro Homoine Inharrime Jangamo Massinga Inhassouro Mabote Morrumbene Panda Vilankulo Zavala

foreach dista in `listall' {

gen `dista'1=.

foreach var of varlist ds1-ds6 {

replace `dista'1=1 if `var'=="`dista'"

} // end of ds1-6 for 1st 'pair foreach'

gen `dista'2=.

foreach var of varlist ds7-ds12 {

replace `dista'2=1 if `var'=="`dista'"

} // end of ds7-ds12 for 1st 'pair foreach'

foreach distb in `listall' {

if "`distb'"=="`dista'" {

continue

}

else {

gen `distb'1=.

foreach var of varlist ds1-ds6 {

replace `distb'1=1 if `var'=="`distb'"

} // end of ds1-6 for 2nd 'pair foreach'

gen `distb'2=.

foreach var of varlist ds7-ds12 {

replace `distb'2=1 if `var'=="`distb'"

} // end of ds7-12 for 2nd 'pair foreach'

count if `dista'1==1 & `distb'1==1

local a=r(N)

count if `dista'2==1 & `distb'2==1

local b=r(N)

count

local all=r(N)

local t=`a'+`b'

if `t'>`all'*0.65 | `t'<`all'*0.35 {

disp "flagged: frequency of `dista' and `distb' is `t'/`all'"

file write bs _n ("`dista'") _tab ("`distb'") _tab (`t'/`all') _tab "both arms"

drop `distb'*

} // end of embedded if for write to file flagged pairs

else {

drop `distb'*

} // end of embedded else for write to file flagged pairs

} // end of first else after distb 'pair foreach'

} // end of distb 'pair foreach'

drop `dista'*

} // end of overall/dista 'pair foreach' - each pair will be assessed twice as both combinations (x+y and y+x)

file close bs

insheet using bias_m10.txt,clear

// Each problem pair is saved twice e.g. as zavala/funhalouro and funhalouro/zavala

// in order to identify duplicates: reshape, sort district, reshape back, drop

gen id=_n

reshape long distr_, i(id) j(newvar)

sort id distr_

by id: gen nv=_n

drop newvar

reshape wide distr_, i(id) j(nv) // now pairs are same way round,, can drop dups

duplicates drop distr_1 distr_2, force

sort freq

drop id

br //

save biasfinal10_mon, replace

!"C:\Program Files\Windows Media Player\wmplayer" C:\Windows\Media\tada.wav

*****************************

*Part III pick final scheme and save

use allowed_tm10, clear // tm10 was the criteria set picked, after 100000 runs 05-04-2013, low bias

set seed 05042013 // = date when final allocation picked randomly from allowed set number 9

generate random = runiform()

sort random

keep if _n==1

list allocation // show the scheme number that was picked

drop dup random allocation // have to drop all numeric variables added onto the end for sxpose

sxpose, clear // had to download the string version of "xpose" which does the same as excel's transpose (wide-->long but NOT stata way)

generate group = group(2) // back to the same two groups as before re-shaped wide in first foreach above

rename group arm

rename _var1 district // for merge with cluster_m.dta/motherchild_m etc

sort district

label data

label data "Final scheme 6102 picked after 100000 runs using criteria set #10 SS 042013"

save inscale_tm10_6102,replace

**Analysis codes (Stata v 13) – Primary and Secondary Outcomes**

**Appropriate treatment by condition and coverage of blood tests for malaria, adjusted for baseline variables**

(fever = suspected malaria; malaria = confirmed malaria; diarrhoea = diarrhoea = pneum = suspected pneumonia; testblood =blood test for malaria; whch_apptreat = whole child appropriate treatment)

xtlogit fever i.inscale bfeverdis bclupub bcluape clusmotf clus1logcost, re i(dist) or

xtlogit malaria i.inscale bmalardis bclupub bcluape clusmotf clus1logcost, re i(dist) or

xtlogit diarrhoea i.inscale bdiarrdis bclupub bcluape clusmotf clus1logcost, re i(dist) or

xtlogit pneum i.inscale bpneudis bclupub bcluape clusmotf clus1logcost, re i(dist) or

xtlogit testblood i.inscale bclupub bcluape clusmotf clus1logcost, re i(dist) or

xtlogit whch_apptreat i.inscale bclusapp clusmotf clus1logcost if provf==1, re i(dist) or

**Rates of care seeking by health provider type**

(vhtf =CHW first port of call; vhtany= CHW at any point pubany = public facility at any point; sector_priva = private sector provider at any point; nocare = no care sought)

xtlogit vhtf i.inscale bcluape_a bclupub bcluape clusmotf clus1logcost, re i(dist) or

xtlogit vhtany i.inscale bcluape_a bclupub bcluape clusmotf clus1logcost, re i(dist) or

xtlogit pubany i.inscale bclupub_a bclupub bcluape clusmotf clus1logcost, re i(dist) or

xtlogit sector_priva i.inscale bclupriv_a bclupub bcluape clusmotf clus1logcost, re i(dist) or // combine because numbers are few

xtlogit nocare i.inscale bclunocare bclupub bcluape clusmotf clus1logcost, re i(dist) or

**Appropriate treatment by condition**

(malapprop = suspected malaria; mal2pprop = confirmed malaria; diapprop = diarrhoea = pnapprop = suspected pneumonia with any eligible antibiotic; pnapprop_a = suspected pneumonia with amoxicillin)

xtlogit malapprop i.inscale bmalap bclupub bcluape clusmotf clus1logcost, re i(dist) or // in wide database only (and below to rows too)

xtlogit mal2pprop i.inscale bmal2ap bclupub bcluape clusmotf clus1logcost, re i(dist) or

xtlogit diapprop i.inscale bdiarzap bclupub bcluape clusmotf clus1logcost , re i(dist) or

xtlogit pnapprop i.inscale bpneuap bclupub bcluape clusmotf clus1logcost, re i(dist) or

xtlogit pnapprop_a i.inscale bpneuap_a bclupub bcluape clusmotf clus1logcost, re i(dist) or

**CHW outcomes**

(APEidentity = social identity score; APEMot = motivation score; perform = clinical knowledge score prop_leave = attrition; anydrug = stockouts of any drugs for common childhood illesse)

xtreg perform i.inscale bclusperf bclupub bcluape clusmotf clus1logcost, i(dist) re // final - in paper

xtreg APEidentity i.inscale bclupub bcluape clusmotf clus1logcost, i(dist) re // final - in paper

xtreg APEMot i.inscale bclupub bcluape clusmotf clus1logcost, i(dist) re // final - in paper

xtreg prop_leave i.inscale bclupub bcluape clusmotf clus1logcost, be i(dist) // - in paper

xtlogit anydrug i.inscale bclupub bcluape clusmotf clus1logcost, re i(dist) or // in paper
