# Supplementary material for: Improving outcomes for children with malaria, diarrhoea and pneumonia in Mozambique: A cluster randomised controlled trial of the inSCALE technology innovation
Source: PLOS Digit Health. 2023 Jun 12;2(6):e0000235. doi: 10.1371/journal.pdig.0000235 (PMC10260254; doi:10.1371/journal.pdig.0000235)
Supplement: S2 Text — (DOCX) [file pdig.0000235.s002.docx]

**S2 Text: data collection forms**

**Improving community health worker treatment for malaria, diarrhoea and pneumonia in Mozambique through the inSCALE mHealth innovation: A cluster randomised controlled trial**

Seyi Soremekun, Karin Källander, Raghu Lingam, Ana-Cristina Castel Branco, Neha Batura, Daniel Strachan, Abel Muiambo, Nelson Salomao, Juliao Condoane, Fenias Benhane, Frida Kasteng, Anna Vassall, Zelee Hill, Guus ten Asbroek, Sylvia Meek, James Tibenderana; and Betty Kirkwood

| **Manejo Integrado de Casos na Comunidade de Doenças na Infância em Moçambique**  INSCALE ENDLINE  QUESTIONÁRIO PARA APE |  | [CÓDIGO DO FORMULÁRIO COLOQUE A ETIQUETA AQUI] | FORMNO |
| --- | --- | --- | --- |

1. **Parte 1: Informações da entrevista**

| Nome do Distrito | | | | | |
| --- | --- | --- | --- | --- | --- |
|  | 1. Funhalouro | 2.Govuro | 3.Homoine | 4. Inharrime | DISTRICT |
|  | 5. Inhassouro | 6. Jangamo | 7. Mabote | 8. Massinga |  |
|  | 9. Morrumbene | 10. Panda | 11. Vilanculos | 12. Zavala |  |

| Localidade |  | LOC |
| --- | --- | --- |

| Área de Enumeração |  |  |  | EA |
| --- | --- | --- | --- | --- |

| Nome da Vila |  | VILLAGE |
| --- | --- | --- |

| Código da Entrevista |  |  | ICODE |
| --- | --- | --- | --- |

| Data da Entrevista (DDMM20AA) |  |  |  |  | 20 |  |  | IDATE |
| --- | --- | --- | --- | --- | --- | --- | --- | --- |

| O consentimento informado escrito foi fornecido? **[O consentimento informado é OBRIGATÓRIO para cada formulário preenchido – por favor faça um círculo para mostrar que você leu o termo de Consentimento e o entrevistado aceitou]** | | | |
| --- | --- | --- | --- |
| Sim | 1 |  | CONSENT |

| **[Por favor, seleccione se o APE trabalha na área de tecnologia móvel inSCALE ou Área de Controlo].** | | | |
| --- | --- | --- | --- |
| Área de Controlo | 0 |  | VHTARM |
| Tecnologia (telemóvel) | 2 |  |  |

1. **Parte 2: Situação socio-económica e demográfica**

| Qual é o seu nome [Nome oficial] |  | VHTNAME |
| --- | --- | --- |

| Tem outro nome pelo qual as pessoas na comunidade conhecem? [nome pelo qual é mais conhecido] |  | VHTNICK |
| --- | --- | --- |

| **[Observar o sexo do/a entrevistado/a]** | | | |
| --- | --- | --- | --- |
| Masculino | 1 |  | VHTSEX |
| Feminino | 2 |  |  |

| Em que ano nasceu? **[AAAA]** | **Não Sabe=9999** |  |  |  |  | VHTYOB |
| --- | --- | --- | --- | --- | --- | --- |

| Em que mês nasceu? | | | | | | |
| --- | --- | --- | --- | --- | --- | --- |
| 1  Janeiro | 2  Fevereiro | 3  Março | 4  Abril | 5  Maio | 6  Junho | VHTMOB |
| 7  Julho | 8  Agosto | 9  Setembro | 10  Outubro | 11  Novembro | 12  Dezembro |  |
| 99  Não Sabe |  |  |  |  |  |  |

| Qual é a sua língua materna? | | | | | |  |
| --- | --- | --- | --- | --- | --- | --- |
| 1  Bitonga | 2  Chitsua | 3  ChiChopi | 4  Outra [ESPECIFIQUE ABAIXO] | | VHTLANG |  |
| *Se ‘Outra’ Especifique aqui____________________________________________*___________________________ | | | | VHTLANGO | | |

| Qual é a sua religião? | | | | | |  |
| --- | --- | --- | --- | --- | --- | --- |
| 1  Cristã | 2  Muçulmana | 3  Não tem religião | 88  Outra [ESPECIFIQUE ABAIXO] | | VHTFAITH |  |
| *Se ‘Outra’ Especifique aqui____________________________________________*___________________________ | | | | VHTFAITHO | | |

| Você está actualmente solteiro, casado, vivendo com um parceiro, viúvo, divorciado ou separado? | | | | |
| --- | --- | --- | --- | --- |
| 1  Casado | 2  Maritalmente/ União de facto | 3  Viúvo | 4  Divorciado | MARRIED |
| 5  Separado | 6  Solteiro |  | |  |

| Qual é o nível de educação mais alto em que concluiu? **[responder mesmo se o entrevistado ainda estiver a estudar]** | | | | |
| --- | --- | --- | --- | --- |
| 1  Nenhuma | 2  Primário - Incompleto | 3  Primária | 4  Secundário | VHTED |
| 5  Pré-Universitário | 6  Técnico | 7  Profissional / Estágio | 8  Nível Universitário |  |

| Qual é a sua principal ocupação? | | | | | |
| --- | --- | --- | --- | --- | --- |
| 1 | Empregado do Sector Público: profissional / semi-qualificado | 6 | Trabalhadores por conta própria: comerciante / motorista de táxi / pequeno negócio | | VHTOCCPM |
| 2 | Empregado do Sector Público: manual/trabalhador assalariado | 7 | Trabalhadores por conta própria: agricultor/pescador | |  |
| 3 | Empregado privado /ONG: profissional / semi-qualificado | 8 | Doméstica | |  |
| 4 | Empregado privado /ONG: manual/trabalhador assalariado | 9 | Desempregado | |  |
| 5 | Trabalhador ocasional ou diário | 10 | Doente/ Incapaz de trabalhar, deficiente | |  |
| **Se as opções forem 1-5, vá para 2.11]** | | 11 | Estudante | |  |
|  |  | **[Se as opções de 6-11, vá para 2,12]** | | |  |
| 88 | Outra |  |  |  |  |
| *Se ‘Outra’ Especifique _____________________________________________*_______________ | | | | **[vá para 2.11]** | VHTOCUPO |

| **Se na pergunta 2.10 as opções foram 1-5, pergunte:] Quanto é que você foi pago no último mês em que trabalhou?** | | | | |
| --- | --- | --- | --- | --- |
| 1  MTN: 0 - <640,00 | 2  MTN: 640,00 - <1.600,00 | 3  MTN: 1600,00 - <3.200,00 | 4  MTN: 3.200,00 ou mais | VHTMONTH |

| 1  MTN: 0- <1000,00 | 2  MTN: 1000,00 - <3.400,00 | 3  MTN: 3400,00 - <5.200,00 | 4  MTN: 5.200,00 ou mais | RSPMONTH |
| --- | --- | --- | --- | --- |

| Quantos membros da sua família estão actualmente a trabalhar no total (incluindo você)? |  |  | MHHWORK |
| --- | --- | --- | --- |

| Qual é a renda média mensal em dinheiro de todos os salários / vencimentos / produzidos no seu agregado familiar?**[se necessário, permitir que ao inquirido para verificar com chefe de família / outros membros]** | | | | |
| --- | --- | --- | --- | --- |
| 1  MTN: 0.0/mês | 2  MTN: 1 - < 640,00/mês | 3  MTN: 640,00-< 1.280,00 | 4  MTN:1.280,00-< 2.560,00 | HHINCOME |
| 5  MTN: 2.560,00-< 3.840,00 | 6  MTN:3.840,00-< 6.400,00 | 7  MTN: 6.400,00 ou mais por mês | 99  Não sabe |  |

| 1  MTN: 0.0/mês | 2  MTN: 1 - < 1000,00/mês | 3  MTN: 1000,00-< 2.500,00 | 4  MTN:2.500,00-< 3.200,00 | HHINCOME |
| --- | --- | --- | --- | --- |
| 5  MTN: 3.200,00-< 5.200,00 | 6  MTN:5.200,00-< 10.000,00 | 7  MTN: 10.000,00 ou mais por mês | 99  Não sabe |  |

| Será que algum dos membros do agregado familiar possui um dos seguintes meios de transporte? [Leia a lista e seleccione no sim ou não] | | | | | |
| --- | --- | --- | --- | --- | --- |
|  | Um carrinho de tracção animal | Sim | 1 |  | CART |
|  |  | Não | 0 |  |  |
|  | Bicicleta | Sim | 1 |  | BICYCLE |
|  |  | Não | 0 |  |  |
|  | Uma mota | Sim | 1 |  | SCOOTE |
|  |  | Não | 0 |  |  |
|  | Um carro/camião | Sim | 1 |  | CAR |
|  |  | Não | 0 |  |  |
|  | Um barco/canoa sem motor | Sim | 1 |  | BOAT |
|  |  | Não | 0 |  |  |
|  | Um barco/canoa a motor | Sim | 1 |  | MBOAT |
|  |  | Não | 0 |  |  |
| Outro [especifique ]______________________________________________ | |  | | | OTRPO |

| Qual é a PRINCIPAL fonte de iluminação para a casa? | | | |
| --- | --- | --- | --- |
| Lanterna de Querosene / parafina | 1 |  | HHLIGHT |
| Vela de parafina | 2 |  |  |
| Lenha | 3 |  |  |
| Vela (Cera) | 4 |  |  |
| Eletricidade | 5 |  |  |
| Energia solar | 6 |  |  |
| Lâmpada do telemóvel | 7 |  |  |
| Outra [ESPECIFIQUE ABAIXO] | 88 |  |  |
| *Se ‘Outro’ especifique ________________________________________________*____________________________ | | | HHLIGHTO |

| Será que o seu agregado familiar tem ALGUM dos seguintes elementos a funcionar bem? **[Leia a lista e seleccione sim / não]** | | | | | |
| --- | --- | --- | --- | --- | --- |
|  | Eletricidade (linha fixa) | 1. SIM | 0. NÃO |  | ELECTR |
|  | Energia solar / gerador | 1. SIM | 0. NÃO |  | SOLAR |
|  | Radio | 1. SIM | 0. NÃO |  | RADIO |
|  | A televisão | 1. SIM | 0. NÃO |  | TELVI |
|  | Um telemóvel | 1. SIM | 0. NÃO |  | MPHONE |
|  | Um telefone fixo | 1. SIM | 0. NÃO |  | FPHONE |
|  | Uma geleira | 1. SIM | 0. NÃO |  | REFRIG |
|  | Uma cama | 1. SIM | 0. NÃO |  | BED |
|  | Um colchão de espuma | 1. SIM | 0. NÃO |  | MATRESS |
|  | Um sofá | 1. SIM | 0. NÃO |  | SOFA |
|  | Roupeiro (para vestuário) | 1. SIM | 0. NÃO |  | CUPBOARD |
|  | Um relógio | 1. SIM | 0. NÃO |  | CLOCK |
|  | Uma rede mosquiteira que pode ser usado enquanto dorme | 1. SIM | 0. NÃO |  | NET |

| O seu filho mais novo dormiu na última noite sob uma rede mosquiteira? | | | |
| --- | --- | --- | --- |
| Sim | 1 |  | CHILDNET |
| Não | 0 |  |  |
| Não Sabe | 99 |  |  |
| Não é aplicável – não possui uma rede | 77 |  |  |

| No total, quantas crianças com idade inferior a 5 anos dormiram debaixo de uma rede mosquiteira na noite passada? **Não Sabe=99, Não se aplica/não tem rede=77** |  |  | NUMNET |
| --- | --- | --- | --- |

| Será que algum membro do seu agregado familiar possui alguns dos seguintes animais?[Não incluir gatos ou cães]: [preencher o número para cada animal (ex: 5 = 005), ou 000 se nenhum **[Preencha 999 se agregado familiar possui um tipo de animal, mas o número de animais é desconhecida]** | | | | | | |
| --- | --- | --- | --- | --- | --- | --- |
|  | Bois |  |  |  |  | COWS |
|  | Cavalo, burro ou mula |  |  |  |  | HORSES |
|  | Cabrito |  |  |  |  | GOATS |
|  | Ovelha |  |  |  |  | SHEEP |
|  | Porco |  |  |  |  | PIGS |
|  | Galinha, Patos, Peru ou Coelho |  |  |  |  | CHDUTURA |
|  | Colmeias (em uso) |  |  |  |  | BEEHIVES |
|  | Outro [ESPECIFIQUE ABAIXO] |  |  |  |  | ANIMALO |
| *Se ‘Outro’ especifique ________________________________________________*__________ | | | | | | ANIMO |

| Será que algum membro do seu agregado familiar tem terra própria? | | | |
| --- | --- | --- | --- |
| Sim | 1 |  | HHLAND |
| Não | 0 | **🡪2.24** |  |

| Quanta terra é que os membros da sua família possuem? |  |  |  |  |  | · |  |  | hectares | HHACRES |
| --- | --- | --- | --- | --- | --- | --- | --- | --- | --- | --- |
| **[Use o seu conversor, se a medida não é dada em hectares. Faça o seu cálculo na íntegra neste formulário e mostrar o seu supervisor para confirmação]** | **Exemplo; se a resposta for um e meio de hectares,**  **preencha 001,50, se a resposta for de três hectares**  **preencher 003,00**  **Não sei = 999,99** | | | | | | | | | |
|  |  | | | | | | | | | |

| Da terra que possui quanto são terrenos agrícolas? **Não Sabe=999.99** |  |  |  |  |  | · |  |  | hectares | HHAGRIC |
| --- | --- | --- | --- | --- | --- | --- | --- | --- | --- | --- |

| Quanta terra que você possui é para outros fins, como para negócios ou aluguer? **Não Sabe =999.99** |  |  |  |  |  | · |  |  | hectares | HHOP |
| --- | --- | --- | --- | --- | --- | --- | --- | --- | --- | --- |

| Qual é o PRINCIPAL tipo de combustível utilizado na preparação dos alimentos? | | | |
| --- | --- | --- | --- |
| Lenha | 1 |  | MEFUEL |
| Carvão | 2 |  |  |
| Parafina / Querosene | 3 |  |  |
| Gás | 4 |  |  |
| Eletricidade | 5 |  |  |
| Resíduos /palha /capim | 6 |  |  |
| Excremento de animais | 7 |  |  |
| Energia solar | 8 |  |  |
| Nenhum alimento é preparado na casa | 9 |  |  |
| Outro [ESPECIFIQUE ABAIXO] | 88 |  |  |
| *Se ‘Outro’ especifique aqui ________________________________________________*__________ | | | MEFUELO |

| Qual é a PRINCIPAL fonte de água potável para os membros do seu agregado familiar? | | | |
| --- | --- | --- | --- |
| Água canalizada para casa | 1 |  | MWATER |
| Torneira/Fontenária pública | 2 |  |  |
| Poço desprotegido | 3 |  |  |
| Poço protegido | 4 |  |  |
| Fonte de água desprotegida | 5 |  |  |
| Fonte de água protegida | 6 |  |  |
| Furo | 7 |  |  |
| Rio / Riacho | 8 |  |  |
| Lagoa/lago | 9 |  |  |
| Recolha de águas pluviais | 10 |  |  |
| Compra de água / caminhão | 11 |  |  |
| Outro [ESPECIFIQUE ABAIXO] | 88 |  |  |
| *Se ‘Outro’ especifique aqui ________________________________________________*__________ | | | MWATERO |

# Como você se sente com o seu trabalho?

**Eu gostaria de ler algumas afirmações sobre o seu trabalho como APE. As afirmações vão parecer que você mesmo estará a dizê-las. Por favor, pense sobre cada afirmação e me diga se no momento você concorda totalmente, concorda, é neutro (significa que não concorda nem discorda), discorda, ou discorda totalmente.**

**[**Por **favor dê ao APE a cópia da página inteira da imagem abaixo para usar quando ele/ela responde às seguintes afirmações]**

**
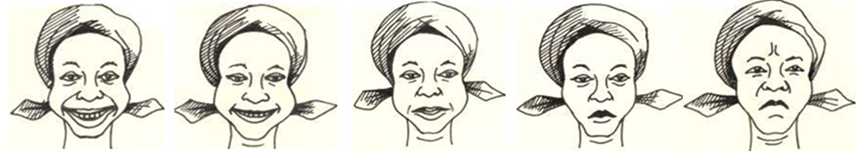
**

| **Concordo totalmente 1** | **Concordo 2** | **Neutro 3** | **Discorda 4** | **Discorda totalmente 5** |
| --- | --- | --- | --- | --- |

**Exemplos das afirmações**

| **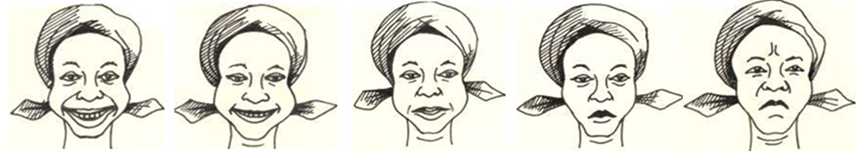**Nesta semana eu achei que o tempo está muito bom | | | | | |  |
| --- | --- | --- | --- | --- | --- | --- |
|  | concordo totalmente 1 | concordo  2 | Neutro  3 | discorda  4 | **Discorda totalmente 5** |  |

**[Leia cada afirmação abaixo e seleccione a resposta que o APE der]**

**Afirmações**

| De um modo geral, estou satisfeito com meu trabalho como APE **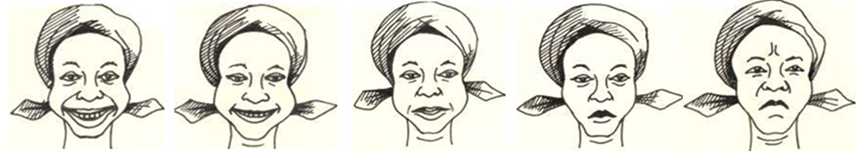** | | | | | |  |
| --- | --- | --- | --- | --- | --- | --- |
|  | concordo totalmente 1 | concordo  2 | neutro  3 | discorda  4 | discorda  totalmente  5 | FSATIS |

| Acho que vale a pena o tempo que gasto nas tarefas que me pediram para fazer | | | | | |  |
| --- | --- | --- | --- | --- | --- | --- |
|  | concordo totalmente 1 | concordo  2 | neutro  3 | discorda  4 | discorda  totalmente  5 | FWORTH |

| O trabalho que eu faço como APE me proporciona o que eu preciso | | | | | |  |
| --- | --- | --- | --- | --- | --- | --- |
|  | concordo totalmente 1 | concordo  2 | neutro  3 | discorda  4 | discorda  totalmente  5 | FNEED |

| É importante que eu faça um bom trabalho como APE só assim o programa do APE funciona bem | | | | | |  |
| --- | --- | --- | --- | --- | --- | --- |
|  | concordo totalmente 1 | concordo  2 | neutro  3 | discorda  4 | discorda  totalmente  5 | FWORKS |

| Sou respeitado na minha comunidade pelo trabalho que faço como APE | | | | | |  |
| --- | --- | --- | --- | --- | --- | --- |
|  | concordo totalmente 1 | concordo  2 | neutro  3 | discorda  4 | discorda  totalmente  5 | FRESPEC |

| No momento, não tenho muita vontade em trabalhar arduamente, como eu poderia | | | | | |  |
| --- | --- | --- | --- | --- | --- | --- |
|  | concordo totalmente 1 | concordo  2 | Neutro  3 | discorda  4 | discorda  totalmente  5 | FMOTIVA |

**
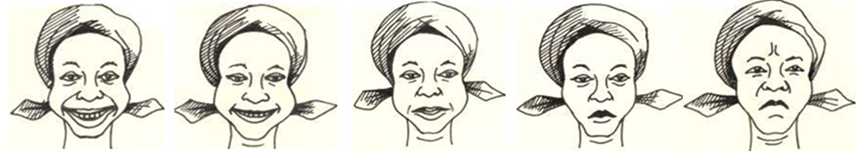
**

| Eu só faço esse trabalho pelos benefícios | | | | | |  |
| --- | --- | --- | --- | --- | --- | --- |
|  | concordo totalmente 1 | concordo  2 | neutro  3 | discorda  4 | discorda  totalmente  5 | FBENEFIT |

| Eu sinto me a vontade em exercer as minhas funções exigidas de mim como APE | | | | | |  |
| --- | --- | --- | --- | --- | --- | --- |
|  | concordo totalmente 1 | concordo  2 | neutro  3 | discorda  4 | discorda  totalmente  5 | FPERF |

| Tenho orgulho de estar a trabalhar como APE | | | | | |  |
| --- | --- | --- | --- | --- | --- | --- |
|  | concordo totalmente 1 | concordo  2 | neutro  3 | discorda  4 | discorda  totalmente  5 | FPROUD |

| Sinto-me comprometido com a minha função de APE | | | | | |  |
| --- | --- | --- | --- | --- | --- | --- |
|  | concordo totalmente 1 | concordo  2 | neutro  3 | discorda  4 | discorda  totalmente  5 | FCOMMIT |

| O que o programa dos APEs quer atingir é o mesmo que eu quero alcançar | | | | | |  |
| --- | --- | --- | --- | --- | --- | --- |
|  | concordo totalmente 1 | concordo  2 | neutro  3 | discorda  4 | discorda  totalmente  5 | FACHIEV |

| Eu tenciono parar de trabalhar como APE | | | | | |  |
| --- | --- | --- | --- | --- | --- | --- |
|  | concordo totalmente 1 | concordo  2 | neutro  3 | discorda  4 | discorda  totalmente  5 | FSTOP |

| Eu faço todo o trabalho que me é esperado para fazer | | | | | |  |
| --- | --- | --- | --- | --- | --- | --- |
|  | concordo totalmente 1 | concordo  2 | neutro  3 | discorda  4 | discorda  totalmente  5 | FCOMPLE |

**
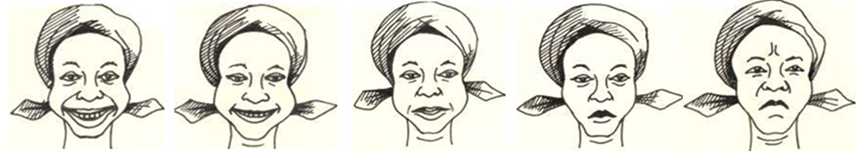
**

| Estou disposto a fazer mais do que se pede a mim como APE | | | | | |  |
| --- | --- | --- | --- | --- | --- | --- |
|  | concordo totalmente 1 | concordo  2 | neutro  3 | discorda  4 | discorda  totalmente  5 | FWILL |

| Às vezes não entendo porque me pedem para fazer certas coisas como APE, mas eu as faço na mesma. | | | | | |  |
| --- | --- | --- | --- | --- | --- | --- |
|  | concordo totalmente 1 | concordo  2 | neutro  3 | discorda  4 | discorda  totalmente  5 | FSENSE |

| Aqueles APEs que são os melhores no seu trabalho, são os que recebem a maioria dos benefícios | | | | | |  |
| --- | --- | --- | --- | --- | --- | --- |
|  | concordo totalmente 1 | concordo  2 | neutro  3 | discorda  4 | discorda  totalmente  5 | FBEST |

| O programa dos APEs fornece-me tudo o que eu preciso para trabalhar corretamente. | | | | | |  |
| --- | --- | --- | --- | --- | --- | --- |
|  | concordo totalmente 1 | concordo  2 | neutro  3 | discorda  4 | discorda  totalmente  5 | FEVERY |

| Suponho que vou continuar a trabalhar como APE no futuro | | | | | |  |
| --- | --- | --- | --- | --- | --- | --- |
|  | concordo totalmente 1 | concordo  2 | neutro  3 | discorda  4 | discorda  totalmente  5 | FSTAY |

| Sugestões dadas pelos APEs sobre como melhorar o seu trabalho, são normalmente ignorados pelos supervisores. | | | | | |  |
| --- | --- | --- | --- | --- | --- | --- |
|  | concordo totalmente 1 | concordo  2 | Neutro  3 | discorda  4 | discorda  totalmente  5 | FIGNORE |

| A supervisão do meu trabalho é boa. | | | | | |  |
| --- | --- | --- | --- | --- | --- | --- |
|  | concordo totalmente 1 | concordo  2 | neutro  3 | discorda  4 | discorda  totalmente  5 | FGOOD |

**
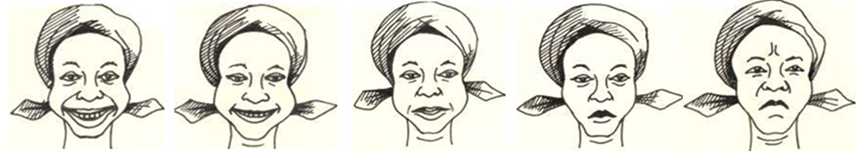
**

| O subsídio que eu recebo reflecte um salário justo sobre as minhas habilidades, conhecimentos, e formação. | | | | | |  |
| --- | --- | --- | --- | --- | --- | --- |
|  | concordo totalmente 1 | concordo  2 | neutro  3 | discorda  4 | discorda  totalmente  5 | FINCOME |

| Se eu fizer bem o meu trabalho como APE, irei conseguir alcançar meus objectivos | | | | | |  |
| --- | --- | --- | --- | --- | --- | --- |
|  | concordo totalmente 1 | concordo  2 | neutro  3 | discorda  4 | discorda  totalmente  5 | FDREAMS |

| Eu consigo resolver a maioria dos problemas que tenho como APE, se eu trabalhar arduamente | | | | | |  |
| --- | --- | --- | --- | --- | --- | --- |
|  | concordo totalmente 1 | concordo  2 | neutro  3 | discorda  4 | discorda  totalmente  5 | FSOLVE |

| Adquiro conhecimento por ser um APE | | | | | |  |
| --- | --- | --- | --- | --- | --- | --- |
|  | concordo totalmente 1 | concordo  2 | neutro  3 | discorda  4 | discorda  totalmente  5 | FKNOW |

| Meu trabalho é importante porque ajudo as pessoas | | | | | |  |
| --- | --- | --- | --- | --- | --- | --- |
|  | concordo totalmente 1 | concordo  2 | neutro  3 | discorda  4 | discorda  totalmente  5 | FHELP |

| O trabalho como APE providencia pagamento aceitável comparado com trabalhos similares | | | | | |  |
| --- | --- | --- | --- | --- | --- | --- |
|  | concordo totalmente 1 | concordo  2 | neutro  3 | discorda  4 | discorda  totalmente  5 | FCOMPAR |

**
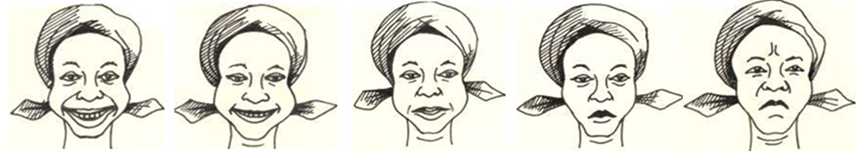
**

| Eu me identifico com o programa dos APEs | | | | | |  |
| --- | --- | --- | --- | --- | --- | --- |
|  | concordo totalmente 1 | concordo  2 | neutro  3 | discorda  4 | discorda  totalmente  5 | FIDENTG |

| Sinto-me ligado ao programa do APE | | | | | |  |
| --- | --- | --- | --- | --- | --- | --- |
|  | concordo totalmente 1 | concordo  2 | neutro  3 | discorda  4 | discorda  totalmente  5 | FCONNG |

|  |  |
| --- | --- |

| Eu sintome parte do programa do APE | | | | | |  |
| --- | --- | --- | --- | --- | --- | --- |
|  | concordo totalmente 1 | concordo  2 | neutro  3 | discorda  4 | discorda  totalmente  5 | FRELATG |

| Sinto-me comprometido com o programa do APE | | | | | |  |
| --- | --- | --- | --- | --- | --- | --- |
|  | concordo totalmente 1 | concordo  2 | neutro  3 | discorda  4 | discorda  totalmente  5 | FCOMMG |

|  | | | | | |  |
| --- | --- | --- | --- | --- | --- | --- |
| Estou contente por ser um APE | | | | | |  |
|  | concordo totalmente 1 | concordo  2 | neutro  3 | discorda  4 | discorda  totalmente  5 | FGLADG |

| Ser APE contribuiu em grande medida do que sou como pessoa | | | | | |  |
| --- | --- | --- | --- | --- | --- | --- |
|  | concordo totalmente 1 | concordo  2 | neutro  3 | discorda  4 | discorda  totalmente  5 | FDEFING |

|  |  |
| --- | --- |

# Parte 4: Papel do APE

| Quantas crianças entre as idades compreendidas dos 2 meses aos cinco anos de idade viu no último MÊS **[Contagem a partir de um livro de registro. No Crianças=00]** | Número |  |  | Se 00  **🡪4.4** | VHTMSEE |
| --- | --- | --- | --- | --- | --- |

| Quanto tempo você gastou, em média, em cada criança neste último mês? **[ex: 45mins = 00HRS, 45MINS]** | **HRS** |  |  | VHTHOUR |
| --- | --- | --- | --- | --- |
|  | **MINS** |  |  | VHTMIN |

| Das consultas em crianças entre as idades de 2 meses e 5 anos de idade no mês passado, quantas acontece em: | |  | | |  |
| --- | --- | --- | --- | --- | --- |
|  | Sua casa? | **Nenhuma=00** |  |  | CONHME |
|  | Na casa dos membros da comunidade **[por exemplo durante as visitas domiciliárias]?** | **Nenhuma=00** |  |  | CONCOM |
|  | Outro local? | **Nenhuma=00** |  |  | CONOTHER |
| *Especifique se foi noutro local*_______________________________________________________ | | | | | CONOTHO |

| Quantas famílias visitou no último mês como APE? - por exemplo, visitas com enfoque na prevenção de doença ou cuidados / seguimento de todas as crianças menores de cinco anos de idade, incluindo recém-nascidos | Número |  |  | Se 00  **🡪4.7** | HHVISIT |
| --- | --- | --- | --- | --- | --- |

| Quanto tempo em media gastou em cada família visitada no mês passado? **[ex: 5mins = 00HRS, 05MINS]** | **HRS** |  |  | HHHOUR |
| --- | --- | --- | --- | --- |
|  | **MINS** |  |  | HHMINS |

| Quanto tempo você gasta no TOTAL a caminhar / viajar de e para visitar as famílias (casa a casa) no mês passado? **[ex: 1hr 15mins = 01HRS, 15MINS]** | **HRS** |  |  | TRHOUR |
| --- | --- | --- | --- | --- |
|  | **MINS** |  |  | TRMINS |

| Será que você se ocupou com uma das atividades de APE abaixo mencionadas no último mês**?****[leia todas as atividades abaixo]** | | | | | | | | |
| --- | --- | --- | --- | --- | --- | --- | --- | --- |
|  | Visita a uma unidade sanitária a pedido do supervisor e / ou receber kits de medicamentos | Sim | | 1 |  | | | HF2WKS |
|  |  | Não | | 0 | **🡪4.7.3** | | |  |
|  | **[Se sim:]** Por quanto tempo? | | **HRS** | | |  |  | HFHRS |
|  | | | **MINS** | | |  |  | HFMINS |

|  | Tratamento/aconselhamento a doentes membros da comunidade com 5 anos e mais velhos (no mês passado)? | Sim | | 1 |  | | OLD2WKS |
| --- | --- | --- | --- | --- | --- | --- | --- |
|  |  | Não | | 0 | **🡪4.7.5** | |  |
|  | **[Se sim:]** Por quanto tempo? | | **HRS** | |  |  | OLDHRS |
|  | | | **MINS** | |  |  | OLDMINS |

|  | Preenchimento do relatório /preparativos/comunicação (no mês passado)? | Sim | | 1 |  | | ADMN2WKS |
| --- | --- | --- | --- | --- | --- | --- | --- |
|  | **[Preenchimento de relatório - pode ser através do preenchimento do livro de registo e /** **ou através do preenchimento e envio de relatórios via celular]** | Não | | 0 | **🡪4.7.7** | |  |
|  | **[Se sim:]** Por quanto tempo? | | **HRS** | |  |  | ADMHRS |
|  | | | **MINS** | |  |  | ADMINS |

|  | Outras actividades no mês passado, exemplo, levar uma criança doente ao centro de saúde, encontros, etc? *Especifique*___________________________________________________________ | | Se nenhum **🡪4.8** | | OAVHT |  |
| --- | --- | --- | --- | --- | --- | --- |
|  | **[Se mencionou outra actividade:]** Por quanto tempo? | **HRS** |  |  | OAHRS | |
|  | | **MINS** |  |  | OAMINS | |

| Nos últimos **3 meses** quantas vezes foi ao centro de saúde para encontrar-se com o seu supervisor, submeter relatórios, ou fazer a reposição de materiais (medicamentos, etc)? | Número |  |  | Se 00  **🡪4.11** | VHTTIMES |
| --- | --- | --- | --- | --- | --- |

| Quanto tempo normalmente leva de e para o centro de saúde, **incluindo as viagens** **o tempo que fica (ou gasta) no centro de saúde**? | **HRS** |  |  | TRPHOUR |
| --- | --- | --- | --- | --- |
|  | **MINS** |  |  | TRPMINS |

| Você teve alguma despesa monetária como consequência do seu trabalho de APE nos últimos **3 meses**? | | | |
| --- | --- | --- | --- |
| Sim | 1 |  | MONET |
| Não | 0 | **🡪4.25** |  |

| **[Se sim:]** Você tem alguma despesa monetária nas viagens que faz de e para o centro de saúde quando vai encontrar-se com seu supervisor, ou levar medicamentos, ou submeter relatórios (incluindo despesas dealimentação) nos últimos **3 meses**? | | | |
| --- | --- | --- | --- |
| Sim | 1 |  | TRAVEL |
| Não | 0 | **🡪4.16** |  |

| **[Se sim]** Quanto? |  |  |  |  |  | **,** |  |  |  | Mts | TRAVAMNT |
| --- | --- | --- | --- | --- | --- | --- | --- | --- | --- | --- | --- |

| Recebe algum reembolso por parte do seu supervisor? | | | |
| --- | --- | --- | --- |
| Sim, algum ou todo o custo | 1 | **🡪4.15** | TRAVREMB |
| Não, nada foi reembolsado | 0 | **🡪4.16** |  |

| **[Se sim:]** Quanto foi reembolsado? |  |  |  |  |  | **,** |  |  |  | MtS | TRAVREMC |
| --- | --- | --- | --- | --- | --- | --- | --- | --- | --- | --- | --- |

| **[Se sim:]** Você teve alguma despesa monetária por estar a usar o seu telefone privado paratrabalhos relacionados com APE nos últimos **3 meses**? | | | |
| --- | --- | --- | --- |
| Sim | 1 |  | MMOBILE |
| Não | 0 | **🡪4.20** |  |

| **[Se sim]** Quanto? |  |  |  |  |  | **,** |  |  |  | Mts | MOBAMNT |
| --- | --- | --- | --- | --- | --- | --- | --- | --- | --- | --- | --- |

| É reembolsado pelo seu supervisor? | | | |
| --- | --- | --- | --- |
| Sim,algum ou todo o custo | 1 | **🡪4.19** | MOBREMB |
| Não, nada foi reembolsado | 0 | **🡪4.20** |  |

| **[Se sim]** Quanto foi reembolsado? |  |  |  |  |  | **,** |  |  |  | Mts | MOBREMC |
| --- | --- | --- | --- | --- | --- | --- | --- | --- | --- | --- | --- |

| **[Se sim:]** Você teve alguma OUTRA despesa por estar a fazer trabalhos relacionados com o programa dos APEs, nos últimos **3 meses?** | | | |
| --- | --- | --- | --- |
| Sim | 1 |  | OTHMONET |
| Não | 0 | **🡪4.25** |  |

| Se sim, especifique as outras actividades pelas quais teve despesas monetárias **[escreva uma actividade em cada linha]** |  |
| --- | --- |
| Outra actividade 1____________________________________________________________________________ | OTHMONE1 |
| Outra actividade 2____________________________________________________________________________ | OTHMONE2 |
| Outra actividade 3___________________________________________________________________________ | OTHMONE3 |
| Outra actividade 4____________________________________________________________________________ | OTHMONE4 |
| Outra actividade 5___________________________________________________________________________ | OTHMONE5 |

| **[Se sim]** Quanto teve de despesas no **total** para as “outras” actividades mencionadas acima na QN 4.21? | | | | | | | | | | |  |
| --- | --- | --- | --- | --- | --- | --- | --- | --- | --- | --- | --- |
|  |  |  |  |  |  | **,** |  |  |  | Mts | MONETEXP |

| Recebe algum reembolso destas despesas por parte do seu supervisor? | | | |
| --- | --- | --- | --- |
| Sim, algum ou todo o custo | 1 | **🡪4.24** | MONETREM |
| Não, nada foi reembolsado | 0 | **🡪4.25** |  |

| **[Se sim]** Quanto foi reembolsado? |  |  |  |  |  | **,** |  |  |  | Mts | MONETREC |
| --- | --- | --- | --- | --- | --- | --- | --- | --- | --- | --- | --- |

| Você recebeu alguma contribuição não-monetárias de indivíduos da sua aldeia, como resultado do seu trabalho como APE nos últimos **3 meses**? Inclui **produtos e/ou serviços**, tal como pessoas que ajudaram numa tarefa ou no trabalho agrícola, ou se você recebeu ofertas de alimentos, materiais etc.? | | | |
| --- | --- | --- | --- |
| Sim | 1 |  | OTHMONET |
| Não | 0 | **🡪S5** |  |

|  | Produtos e serviços 1___________________________________________________________ | | | | | | | | | | | | SERV1 |
| --- | --- | --- | --- | --- | --- | --- | --- | --- | --- | --- | --- | --- | --- |
|  | Montante/quantidade [1 Kg = 001, 2 ovos = 002, 20 ovos=020 | | | | |  | |  | |  | |  | AMOUNT1 |
|  | Unidade por esta quantidade____________________________________________________ | | | | | | | | | | | | QTY1 |
|  | Quanto pode estimar em valor monetário? **[55,000 METICAIS = 055000]** | Mts |  |  |  | |  | |  | |  | | MON1 |

|  | Produtos e serviços 2___________________________________________________________ | | | | | | | | SERV2 |
| --- | --- | --- | --- | --- | --- | --- | --- | --- | --- |
|  | Montante/quantidade | | | |  |  |  |  | AMOUNT2 |
|  | Unidade por esta quantidade____________________________________________________ | | | | | | | | QTY2 |
|  | Quanto pode estimar em valor monetário? **[55,000 METICAIS = 055000]** | Mts |  |  |  |  |  |  | MON2 |

|  | Produtos e serviços 3___________________________________________________________ | | | | | | | | SERV3 |
| --- | --- | --- | --- | --- | --- | --- | --- | --- | --- |
|  | Montante/quantidade | | | |  |  |  |  | AMOUNT3 |
|  | Unidade por esta quantidade_____________________________________________________ | | | | | | | | QTY3 |
|  | Quanto pode estimar em valor monetário? **[55,000 METICAIS = 055000]** | Mts |  |  |  |  |  |  | MON3 |

|  | Produtos e serviços 4___________________________________________________________ | | | | | | | | SERV4 |
| --- | --- | --- | --- | --- | --- | --- | --- | --- | --- |
|  | Montante/quantidade | | | |  |  |  |  | AMOUNT4 |
|  | Unidade por esta quantidade____________________________________________________ | | | | | | | | QTY4 |
|  | Quanto pode estimar em valor monetário? **[55,000 METICAIS = 055000]** | Mts |  |  |  |  |  |  | MON4 |

|  | Produtos e serviços 5___________________________________________________________ | | | | | | | | SERV5 |
| --- | --- | --- | --- | --- | --- | --- | --- | --- | --- |
|  | Montante/quantidade | | | |  |  |  |  | AMOUNT5 |
|  | Unidade por esta quantidade_____________________________________________________ | | | | | | | | QTY5 |
|  | Quanto pode estimar em valor monetário? **[55,000 METICAIS = 055000]** | Mts |  |  |  |  |  |  | MON5 |

| No mês passado, você recebeu alguma remuneração, salário ou contribuições monetárias do Ministério da Saúde ou do seu supervisor ou do governo pelos serviços no programa do APE? | | | |
| --- | --- | --- | --- |
| Sim | 1 |  | SALARY |
| Não | 0 | **🡪S5** |  |

| **[Se Sim]** Quanto é que recebeu? |  |  |  |  |  | **,** |  |  |  | Mts | SALAMNT |
| --- | --- | --- | --- | --- | --- | --- | --- | --- | --- | --- | --- |

# Parte 5: Telefones

**Esta secção é só para ser preenchida se o APE pertence ao distrito com a tecnologia móvel inSCALE (telefones) área de intervenção.**

Confira a sua resposta comQN 1.8 na página um:

Se o APE NÃO trabalha nos distritos com a intervenção da tecnologia móvel, risque com linha dupla nesta secção e vá para secção 7.

Se o APE NÃO trabalha na área de intervenção tecnológica;

Diga:

Agora irei fazer algumas perguntas acerca do seu trabalho relacionado com os telemóveis e acessórios que recebeu como parte do projecto inSCALE da Malaria Consortium*:*

| O seu telefone oferecido pelo projecto inSCALE está a funcionar? **[Mostre uma imagem do telefone e dos acessórios do telefone se tiveres; o telefone deve estar a funcionar HOJE]** | | | |
| --- | --- | --- | --- |
| Sim | 1 | **🡪5.4** | TECMOB |
| Não | 0 |  |  |

| **[Se Não:]** Porque não tens o telefone oferecido como parte do projecto inSCALE? **[Somente a principal razão]** | | | |
| --- | --- | --- | --- |
| Eu nunca recebi um telefone do inSCALE | 1 | **🡪S6** | NOTEC |
| Eu recebi um telefone mais já não funciona | 2 | **🡪5.3** |  |
| Eu recebi mas perdi/roubaram | 3 |  |  |
| Outro | 88 |  |  |
| *Se ‘Outro’ especifique* __________________________________________________________ | |  | NOTECO |

| **[Se está avariado, perdeu ou roubaram:]** Quando foi a ultima vez que teve o telefone do inSCALE a funcionar?**[Leia todas as opções]** | | | |
| --- | --- | --- | --- |
| No mês passado | 1 |  | LASTTEC |
| Mais de um mês, mas menos de 3 meses atrás | 2 |  |  |
| 3 ou mais meses, mas menos de 6 meses atrás | 3 |  |  |
| 6 ou mais messes atrás | 4 |  |  |
| Nunca | 5 | **🡪S6** |  |

**Se o APE nunca recebeu, ou nunca usou um telemóvel com carregador solar e lâmpada (QN 5.3 = 5), risque com linha dupla nesta secção e vá para secção** **6.**

Se alguma vez já teve um telefone e acessórios inSCALE,

## Diga:

Agora irei fazer algumas perguntas acerca do seu trabalho relacionado com os telemóveis e acessórios que recebeu como parte do projecto inSCALE da Malaria Consortium.

[Pergunte a todos os APEs abrangidos na tecnologia móvel inSCALE se NALGUM MOMENTO usaram o telemóvel, mesmo que o telemóvel esteja no momento avariado ou tenha perdido]

| Quantos relatórios semanais submeteu através do telefone nas **últimas 4 semanas**? | Número |  |  | TECREP |
| --- | --- | --- | --- | --- |

| Quanto tempo levou para resumir, escrever e enviar o seu relatório semanal **mais recente** através do seu telefone **[ex.: 10mins = 00HRS, 10MINS]** | | **HRS** | | | |  | |  | REPHOUR | | |
| --- | --- | --- | --- | --- | --- | --- | --- | --- | --- | --- | --- |
|  | | **MINS** | | | |  | |  | REPMIN | | |
| Este é o tempo normal que levas para submeter o relatório semanal? | | | | | | | | | | |  |
| Sim | 1 | | **🡪6.8** | | | | REPTYP | | | |  |
| Não | 0 | |  | | | |  |  |  |  |  |
| **[Se não:]** Porque não?_________________________________________________________ | | | | | | | REPNOREA | | | |  |
| 5.10 Quando você usa o seu telefone inSCALE e os acessórios durante uma consulta com um doente **(por exemplo se usas o contador de frequência respiratória, uso das etapas de consulta de criança doente)** A consulta levaa) Aproximadamente o mesmo tempo, b) mais tempo, ou c) menos tempo do que leva sem usar o telefone celular e acessórios? | | | | | | | | | |  |  |
| Leva cerca de mesmo tempo sem usar o telemóvel/acessórios | | | | 1 |  | | | | TIMECONS |  |  |
| Leva mais tempo sem usar o telemóvel/acessórios | | | | 2 |  | | | |  |  |  |
| Leva menos tempo sem usar o telemóvel/acessórios | | | | 3 |  | | | |  |  |  |

| Tens carregado telefonesde outros membros da comunidade (sem contar com você e sua família) usando o seu carregador solar? | | | | | | |  |
| --- | --- | --- | --- | --- | --- | --- | --- |
| Sim | | 1 | |  | | TECHARGE |  |
| Não | | 0 | | **🡪S6** | |  |  |
| [Se sim:] Durante a **última semana,** quantos telefones carregou que não pertencem a sua família**?** | Número de telefones | |  | |  | NUMCHARG | |

| Recebeu algum pagamento por ter carregado os telefones dos membros da comunidade? | | | |
| --- | --- | --- | --- |
| Sim | 1 |  | TECHPAY |
| Não | 0 | **🡪S6** |  |

| [Se sim] Quanto você costuma receber por telefone carregado? [Preencha mesmo que eles não tenham cobrado qualquer telefones na semana passada] |  |  | **,** |  |  |  | Mts | TEPAYREC |
| --- | --- | --- | --- | --- | --- | --- | --- | --- |

| **[Se sim]** E quanto no total é que você recebeu na **semana passada** de carregar telefones? **[00,000 se não carregou qualquer telemóvel semana passada]** |  |  | **,** |  |  |  | Mts | TRECLAST |
| --- | --- | --- | --- | --- | --- | --- | --- | --- |

| E essa é o valor normal do rendimento que recebe por carregar celulares durante uma semana? | | | |
| --- | --- | --- | --- |
| Sim | 1 | **🡪S6** | TERECTYP |
| Não | 0 |  |  |

| **[Se não]** Quanto é no total o **rendimento normal semanal** por carregar telefones? |  |  | **,** |  |  |  | Mts | TECWKTYP |
| --- | --- | --- | --- | --- | --- | --- | --- | --- |

# Stock de medicamentos e equipamento

**[DIGA:] agora eu gostaria de lhe perguntar sobre os medicamentos que são fornecidos no seu kit**

Em algum momento no mês passado ou nos três últimos meses, teve ruptura de stock de algum dos medicamentos listados abaixo?

|  | Zinco | 1. SIM – no mês passado | 2. SIM, > 1 mês , mas dentro dos últimos três meses | 0. Não |  | ZINC | |
| --- | --- | --- | --- | --- | --- | --- | --- |
|  | SRO | 1. SIM – no mês passado | 2. SIM, > 1 mês , mas dentro dos últimos três meses | 0. Não |  | ORS | |
|  | Amoxicilina (125mg) | 1 SIM – no mês passado | 2. SIM, > 1 mês , mas dentro dos últimos três meses | 0. Não |  | AMOXG | |
|  | Amoxicilina (250mg) | 1. SIM – no mês passado | 2. SIM, > 1 mês , mas dentro dos últimos três meses | 0. Não |  | AMOXP | |
|  | TDRs para malaria | 1. SIM – no mês passado | 2. SIM, > 1 mês , mas dentro dos últimos três meses | 0. Não |  | RDT | |
|  | Coartem azul (AL) | 1. SIM – no mês passado | 2. SIM, > 1 mês , mas dentro dos últimos três meses | 0. Não |  | COARB | |
|  | Coartem amarelo (AL) | 1. SIM – no mês passado | 2. SIM, > 1 mês , mas dentro dos últimos três meses | 0. Não |  | COARY | |
|  | Artesunato supositorio | 1. SIM – no mês passado | 2. SIM, > 1 mês , mas dentro dos últimos três meses | 0. Não |  | RECTAR |  |

| [DIGA:] Tem a funcionar: | | | | | | |
| --- | --- | --- | --- | --- | --- | --- |
|  | Contador de frequência respiratória ou relógio | 1. Presente | 2. Presente, não funcional | 0. Não presente |  | RESPTIME |
|  | Termómetro | 1. Presente | 2. Presente, não funcional | 0. Não presente |  | THERMO |

# Conhecimento e Prática Clínica Prevista

# DIGA:

**Agora nós gostaríamos de lhe perguntar sobre como responderia em situações com crianças doentes com menos de 5 anos de idade.**

**Irei ler algumas histórias de CINCO crianças doentes. Depois de ler, para cada história irei perguntar te oque farias para ajudar a criança.**

**Podes usar o seu álbum seriado da consulta/8 passos ou registos se quiseres.**

**As suas respostas não serão usadas para julgar o seu desempenho pessoal de forma alguma.**

**A informação não será transmitidapara o seu supervisor, centro de saúde, líder comunitário, ou pessoal do Ministério da Saúde a todos os níveis.**

**Nas histórias a seguir, por favor, suponha que você tem todas as drogas que você precisa, todo o seu equipamento está em ordem e há um serviço de referência por perto.**

**Use o máximo de detalhes que puder para responder as perguntas em cada história;**

**Depois de eu ler a questão seguinte para cada história você não pode voltar a uma pergunta anterior e alterar a resposta que já deu.**

***Vá para a página seguinte 🡪***

**LEIA A HISTÓRIA PARA O APE:**

HISTÓRIA 1: ALBERTO

| **CARTÃO1:** **Alberto é um menino de 4 meses que veio até você através da avó que cuida dele. A avó está preocupada porque ele tem tido tosse e diarreia recentemente.** Por favor agora diz me, devagar, em suas próprias palavras as perguntas que você iria fazer a avó e as ações que você levaria para descobrir o que se passa com Alberto. Nesta fase, você não precisa dizer me como você o trataria, apenas como você iria diagnosticar, a doença que ele tem. |
| --- |

**[NÃO LER as opções abaixo para o APE e NÃO SUGERIR.**

**Se o APE menciona espontaneamente qualquer das seguintes acções na lista, faça um círculo no 1=SIM ao lado da acção. Quando ele/a terminar pergunte: “É tudo?” e continue a perguntar até que não tenha mais respostas para dar. Quando tiver terminado, faça círculos nas restantes que NÃO foram mencionadas como 0=NÃO].**

**[DIAGNOSIS]**

| Pergunta sobre a duração da tosse da criança | **Sim**  **1** | **Não**  **0** | DURC |  | Pergunta sobre a duração da diarreia infantil | **Sim**  **1** | **Não**  **0** | DURD |
| --- | --- | --- | --- | --- | --- | --- | --- | --- |
| Pergunta se criança teve febre | **Sim**  **1** | **Não**  **0** | FEV |  | Pergunta sobre a duração da febre da criança | **Sim**  **1** | **Não**  **0** | DURF |
| Pergunta se tem sangue nas fezes da criança | **Sim**  **1** | **Não**  **0** | BSTOOL |  | Pergunta / verifica se criança pode beber / amamentar | **Sim**  **1** | **Não**  **0** | NILM |
| Pergunta se a criança tem vómitos | **Sim**  **1** | **Não**  **0** | VOM |  | Pergunta / verifica se a criança é muito sonolenta / perde consciência | **Sim**  **1** | **Não**  **0** | LETH |
| Pergunta se criança teve convulsões | **Sim**  **1** | **Não**  **0** | CONV |  | Pergunta se criança teve qualquer outro problema | **Sim**  **1** | **Não**  **0** | OPRB |
|  |  |  |  |  |  |  |  |  |
| Contagens de frequência respiratória na criança | **Sim**  **1** | **Não**  **0** | CBRE |  | Verifica a existência de a tiragem | **Sim**  **1** | **Não**  **0** | INDR |
| Faz o TDR (teste rápido de sangue para a malária) na criança | **Sim**  **1** | **Não**  **0** | RDT |  |  |  |  |  |
|  |  |  |  |  |  |  |  |  |

| **CARTÃO 2: Depois de fazer perguntas para a avó e de ter examinado o Alberto de 4 meses de idade, você percebe que ele teve diarreia por 2 dias sem sangue, tosse por 5 dias e uma frequência respiratória de 56 respirações por minuto. E confirma que não há outros sintomas.** Diga me primeiro - O que acha que tem o Alberto?Como vais controlar a doença desta criança, incluindo qualquer tipo de tratamento ou que conselho você daria? |
| --- |

**[NÃO LER as opções abaixo para o APE e NÃO SUGERIR.**

**Se o APE menciona espontaneamente qualquer das seguintes acções na lista, faça um círculo no 1=SIM ao lado da acção. Quando ele/a terminar pergunte: “É tudo?” e continue a perguntar até que não tenha mais respostas para dar. Quando tiver terminado, faça círculos nas restantes que NÃO foram mencionadas como 0=NÃO].**

**-**

**[DIAGNOSIS]**

| 7.2.1 Identifica a criança como tendo respiração rápida / pneumonia | **Sim**  **1** | **Não**  **0** | FASB |  | 7.2.2 Identifica a criança como tendo malária | **Sim**  **1** | **Não**  **0** | MAL |
| --- | --- | --- | --- | --- | --- | --- | --- | --- |
| 7.2.3 Identifica a criança como tendo sinal de perigo / sintoma para referir ou doença grave (qualquer) | **Sim**  **1** | **Não**  **0** | SEV |  | 7.2.4 Identifica a criança como tendo diarreia | **Sim**  **1** | **Não**  **0** | DIAR |
| **[TREATMENT]** |  |  |  |  |  |  |  |  |
| Prescreve antibióticos; amoxicilina | **Sim**  **1** | **Não**  **0** | PAMO |  | Prescreve anti-maláricos; Coartem | **Sim**  **1** | **Não**  **0** | PAL |
| Prescreve comprimidos de zinco | **Sim**  **1** | **Não**  **0** | PZN |  | Prescreve SRO | **Sim**  **1** | **Não**  **0** | PORS |
| Recomenda dose de AL / Coartem - 3 dias | **Sim**  **1** | **Não**  **0** | DAL |  | Recomenda dose de amoxicilina - 5 dias | **Sim**  **1** | **Não**  **0** | DAMO |
| Recomenda dose de SRO- quantas vezes for necessário | **Sim**  **1** | **Não**  **0** | DORS |  | Recomenda dose de zinco - 10 dias | **Sim**  **1** | **Não**  **0** | DZN |
| Dá primeira dose de amoxicilina | **Sim**  **1** | **Não**  **0** | FAMO |  | Dá primeira dose da AL / Coartem | **Sim**  **1** | **Não**  **0** | FAL |
| Dá primeira dose de SRO | **Sim**  **1** | **Não**  **0** | FORS |  | Dá dose de rectal artesunato | **Sim**  **1** | **Não**  **0** | FRA |
| Dá primeira dose de zinco | **Sim**  **1** | **Não**  **0** | FZN |  | Criança não precisa de nenhum tratamento do APE | **Sim**  **1** | **Não**  **0** | WELL |
| **[REFERRAL]** |  |  |  |  |  |  |  |  |
| Referiu à unidade de sanitária | **Sim**  **1** | **Não**  **0** | REF |  | Passou uma guia de transferência | **Sim**  **1** | **Não**  **0** | NOTE |
| **[ADVICE AND VISIT RECORDING]** |  |  |  |  |  |  |  |  |
| Aconselha a utilização de Rede Mosquiteira Tratada na criança | **Sim**  **1** | **Não**  **0** | AITN |  | Aconselha a continuar a alimentar e a dar líquidos na criança | **Sim**  **1** | **Não**  **0** | AFLU |

| Marca uma visita de seguimento | **Sim**  **1** | **Não**  **0** | FOL |  | Aconselha para voltar ou levar a criança para unidade sanitária se agravar | **Sim**  **1** | **Não**  **0** | AWRS |
| --- | --- | --- | --- | --- | --- | --- | --- | --- |
| Regista as visitas no livro de Registos de Consultas | **Sim**  **1** | **Não**  **0** | VREC |  | Verifica o estado de saúde de criança ou cartão de vacinas | **Sim**  **1** | **Não**  **0** | CREC |

HISTÓRIA 2: TINA

| **CARTÃO 3: Tina foi levada ao posto de saúde onde trabalhas. Ela é uma menina de 2 anos e foi acompanhada pela sua irmã mais velha. Ela tem tido febre por já alguns dias e não tem se alimentado bem e também não dorme bem.** Por favor diz me agora, devagar, com suas próprias palavras as perguntas que você iria fazer a irmã da Tina as acções que levaria para descobrir o que tem a Tina. Nesta fase, você não precisa dizer me como você o trataria, apenas como você iria diagnosticar, a doença que ele tem. |
| --- |

**[NÃO LER as opções abaixo para o APE e NÃO SUGERIR.**

**Se o APE menciona espontaneamente qualquer das seguintes acções na lista, faça um círculo no 1=SIM ao lado da acção. Quando ele/a terminar pergunte: “É tudo?” e continue a perguntar até que não tenha mais respostas para dar. Quando tiver terminado, faça círculos nas restantes que NÃO foram mencionadas como 0=NÃO].**

**[DIAGNOSIS]**

| Pergunta sobre a duração da febre da criança | **Sim**  **1** | **Não**  **0** | DURF2 |  | Pergunta se criança teve diarreia | **Sim**  **1** | **Não**  **0** | ADIA2 |
| --- | --- | --- | --- | --- | --- | --- | --- | --- |
| Pergunta se criança teve tosse | **Sim**  **1** | **Não**  **0** | CGH2 |  | Pergunta se criança tem vomitado | **Sim**  **1** | **Não**  **0** | VOM2 |
| Pergunta se a criança teve convulsões | **Sim**  **1** | **Não**  **0** | CONV2 |  | Pergunta / verifica se criança pode beber / alimentar | **Sim**  **1** | **Não**  **0** | **NILM2** |
| Pergunta se a criança teve outro problema | **Sim**  **1** | **Não**  **0** | OPRB2 |  | Pergunta / verifica se a criança é muito sonolenta / perde consciência | **Sim**  **1** | **Não**  **0** | LETH2 |
|  |  |  |  |  |  |  |  |  |
|  |  |  |  |  |  |  |  |  |
| Contagens de frequência respiratório na criança | **Sim**  **1** | **Não**  **0** | CBRE2 |  | Verifica se há a tiragem | **Sim**  **1** | **Não**  **0** | INDR2 |
| Faz TDR (teste rápido de sangue para a malária) na criança | **Sim**  **1** | **Não**  **0** | RDT2 |  |  |  |  |  |
|  |  |  |  |  |  |  |  |  |

| **CARTÃO 4: Descobres com a irmã da Tina, que ela tem febre que dura ha já 5, 6 dias. Tina também conseguiu comer uma papa um pouco antes de chegar ao centro de saúde. Examinas a Tina e sentes que ela está quente. Fazes TDR a Tina usando o sangue do dedo. O TDR é positivo. Não há outros sintomas.** Diga me primeiro - O que acha que tem a Tina?Como vais controlar a doença da Tina, incluindo qualquer tipo de tratamento ou conselho que você daria? |
| --- |

**[NÃO LER as opções abaixo para o APE e NÃO SUGERIR.**

**Se o APE menciona espontaneamente qualquer das seguintes acções na lista, faça um círculo no 1=SIM ao lado da acção. Quando ele/a terminar pergunte: “É tudo?” e continue a perguntar até que não tenha mais respostas para dar. Quando tiver terminado, faça círculos nas restantes que NÃO foram mencionadas como 0=NÃO].**

**[DIAGNOSIS]**

| 7.5.1 Identifica a criança como tendo frequência respiratório alta/ pneumonia | **Sim**  **1** | **Não**  **0** | FASB2 |  | 7.5.2 Identifica a criança como tendo malária | **Sim**  **1** | **Não**  **0** | MAL2 |
| --- | --- | --- | --- | --- | --- | --- | --- | --- |
| 7.5.3 Identifica a criança como tendo sinal de perigo / sintomas para referir ou uma doença grave (qualquer) | **Sim**  **1** | **Não**  **0** | SEV2 |  | 7.5.4 Identifica a criança como tendo diarreia | **Sim**  **1** | **Não**  **0** | DIAR2 |
| **[TREATMENT]** |  |  |  |  |  |  |  |  |
| Prescreve antibióticos; amoxicilina | **Sim**  **1** | **Não**  **0** | PAMO2 |  | Prescreve anti-maláricos; Coartem | **Sim**  **1** | **Não**  **0** | PAL2 |
| Prescreve comprimidos de zinco | **Sim**  **1** | **Não**  **0** | PZN2 |  | Prescreve SRO | **Sim**  **1** | **Não**  **0** | PORS2 |
| Recomenda dose de AL / Coartem - 3 dias | **Sim**  **1** | **Não**  **0** | DAL2 |  | Recomenda dose de amoxicilina - 5 dias | **Sim**  **1** | **Não**  **0** | DAMO2 |
| Recomenda dose de SRO- quantas vezes for necessário | **Sim**  **1** | **Não**  **0** | DORS2 |  | Recomenda dose de zinco - 10 dias | **Sim**  **1** | **Não**  **0** | DZN2 |
| Dá primeira dose de amoxicilina | **Sim**  **1** | **Não**  **0** | FAMO2 |  | Dá primeira dose da AL / Coartem | **Sim**  **1** | **Não**  **0** | FAL2 |
| Dá primeira dose de SRO | **Sim**  **1** | **Não**  **0** | FORS2 |  | Dá dose de rectal artesunato | **Sim**  **1** | **Não**  **0** | FRA2 |
| Dá primeira dose de Zinco | **Sim**  **1** | **Não**  **0** | FZN2 |  | Criança não precisa de nenhum tratamento do APE | **Sim**  **1** | **Não**  **0** | WELL2 |
| **[REFERRAL]** |  |  |  |  |  |  |  |  |
| Referiu à unidade de sanitária | **Sim**  **1** | **Não**  **0** | REF2 |  | Passou uma guia de transferência | **Sim**  **1** | **Não**  **0** | NOTE2 |
| **[ADVICE AND VISIT RECORDING]** |  |  |  |  |  |  |  |  |
| Aconselha a utilização de Rede Mosquiteira Tratada na criança | **Sim**  **1** | **Não**  **0** | AITN2 |  | Aconselha a continuar a alimentar e a dar líquidos na criança | **Sim**  **1** | **Não**  **0** | AFLU2 |

| Marca uma visita de seguimento | **Sim**  **1** | **Não**  **0** | FOL2 |  | Aconselha para voltar ou levar a criança para unidade sanitária se agravar | **Sim**  **1** | **Não**  **0** | AWRS2 |
| --- | --- | --- | --- | --- | --- | --- | --- | --- |
| Regista as visitas no livro de Consultas | **Sim**  **1** | **Não**  **0** | VREC2 |  | Verifica o estado de saúde de criança ou cartão de vacinas | **Sim**  **1** | **Não**  **0** | CREC2 |

STORY 3: LUISA

| **CARTÃO 5: Luisa é uma menina de 3 anos e meio de idade. A mãe trouxe a menina a sua casa a noite, porque ela teve tosse que foi piorando e ela está tendo dificuldade para respirar** **Você descobre que Luisa tem tosse por quase um mês. Você examina e vê que ela tem tiragem, mas sem outros sintomas.** Diga me primeiro - O que acha que têm a Luisa? **E,**Como vais controlar a doença da Luisa, incluindo qualquer tipo de tratamento ou conselho que você daria? |
| --- |

**[NÃO LER as opções abaixo para o APE e NÃO SUGERIR.**

**Se o APE menciona espontaneamente qualquer das seguintes acções na lista, faça um círculo no 1=SIM ao lado da acção. Quando ele/a terminar pergunte: “É tudo?” e continue a perguntar até que não tenha mais respostas para dar. Quando tiver terminado, faça círculos nas restantes que NÃO foram mencionadas como 0=NÃO].**

**[DIAGNOSIS]**

| 7.7.1 Identifica a criança como tendo frequência respiratório alta/ pneumonia | **Sim**  **1** | **Não**  **0** | FASB3 |  | 7.7.2 Identifica a criança como tendo diarreia / malaria | **Sim**  **1** | **Não**  **0** | MALD3 |
| --- | --- | --- | --- | --- | --- | --- | --- | --- |
| 7.7.3 Identifica a criança como tendo sinal de perigo / sintomas para referir ou uma doença grave (qualquer) | **Sim**  **1** | **Não**  **0** | SEV3 |  | 7.7.4 A criança não tem nenhum problema grave | **Sim**  **1** | **Não**  **0** | WELL3 |
| **[TREATMENT]** |  |  |  |  |  |  |  |  |
| Prescreve antibióticos; amoxicilina | **Sim**  **1** | **Não**  **0** | PAMO3 |  | Prescreve anti-maláricos; Coartem | **Sim**  **1** | **Não**  **0** | PAL3 |
| Prescreve comprimidos de zinco | **Sim**  **1** | **Não**  **0** | PZN3 |  | Prescreve SRO | **Sim**  **1** | **Não**  **0** | PORS3 |
| Recomenda dose de AL / Coartem - 3 dias | **Sim**  **1** | **Não**  **0** | DAL3 |  | Recomenda dose de amoxicilina - 5 dias | **Sim**  **1** | **Não**  **0** | DAMO3 |
| Recomenda dose de SRO- quantas vezes for necessário | **Sim**  **1** | **Não**  **0** | DORS3 |  | Recomenda dose de zinco - 10 dias | **Sim**  **1** | **Não**  **0** | DZN3 |
| Dá primeira dose de amoxicilina | **Sim**  **1** | **Não**  **0** | FAMO3 |  | Dá primeira dose da AL / Coartem | **Sim**  **1** | **Não**  **0** | FAL3 |
| Dá primeira dose de SRO | **Sim**  **1** | **Não**  **0** | FORS3 |  | Dá dose de rectal artesunato | **Sim**  **1** | **Não**  **0** | FRA3 |
| Dá primeira dose de Zinco | **Sim**  **1** | **Não**  **0** | FZN3 |  | Criança não precisa de nenhum tratamento do APE | **Sim**  **1** | **Não**  **0** | WELL3 |

**[REFERRAL]**

| Referiu à unidade de sanitária | **Sim**  **1** | **Não**  **0** | REF3 |  | Passou uma guia de transferência | **Sim**  **1** | **Não**  **0** | NOTE3 |
| --- | --- | --- | --- | --- | --- | --- | --- | --- |
| **[ADVICE AND VISIT RECORDING]** |  |  |  |  |  |  |  |  |
| Aconselha a utilização de Rede Mosquiteira Tratada na criança | **Sim**  **1** | **Não**  **0** | AITN3 |  | Aconselha a continuar a alimentar e a dar líquidos na criança | **Sim**  **1** | **Não**  **0** | AFLU3 |
| Marca uma visita de seguimento | **Sim**  **1** | **Não**  **0** | FOL3 |  | Aconselha para voltar ou levar a criança para unidade sanitária se agravar | **Sim**  **1** | **Não**  **0** | AWRS3 |
| Regista a visita no livro de Consultas | **Sim**  **1** | **Não**  **0** | VREC3 |  | Verifica o estado de saúde de criança ou o cartão de vacinas | **Sim**  **1** | **Não**  **0** | CREC3 |

HISTÓRIA 4: FABIÃO

| **CARTÃO 6: Fabião é um menino de 10 meses que foi levado a sua casa pelo seu pai, porque ele está com corpo quente a 10 dias, e não consegue comer nada há um dia e meio, porque não consegue ficar acordado. Você examina Fabião e vê que ele não está se movendo muito e seus olhos estão fechados. Você sacode o braço e bate as palmas na frente de seu rosto, mas ele não responde e seus olhos permanecem fechados. Não há outros problemas.** Diga me primeiro - O que acha que têm o Fabião? **E,**Como vais controlar a doença do Fabião, incluindo qualquer tipo de tratamento ou conselho que você daria? |
| --- |

**[NÃO LER as opções abaixo para o APE e NÃO SUGERIR.**

**Se o APE menciona espontaneamente qualquer das seguintes acções na lista, faça um círculo no 1=SIM ao lado da acção. Quando ele/a terminar pergunte: “É tudo?” e continue a perguntar até que não tenha mais respostas para dar. Quando tiver terminado, faça círculos nas restantes que NÃO foram mencionadas como 0=NÃO].**

**[DIAGNOSIS]**

| 7.9.1 Identifica a criança como tendo frequência respiratório alta/ pneumonia | **Sim**  **1** | **Não**  **0** | FASB4 |  | 7.9.2 Identifica a criança como tendo malária | **Sim**  **1** | **Não**  **0** | MAL4 |
| --- | --- | --- | --- | --- | --- | --- | --- | --- |
| 7.9.3 Identifica a criança como tendo sinal de perigo / sintomas para referir ou uma doença grave (qualquer) | **Sim**  **1** | **Não**  **0** | SEV4 |  | 7.9.4 Identifica a criança como tendo diarreia | **Sim**  **1** | **Não**  **0** | DIAR4 |
| **[TREATMENT]** |  |  |  |  |  |  |  |  |
| Prescreve antibióticos; amoxicilina | **Sim**  **1** | **Não**  **0** | PAMO4 |  | Prescreve anti-maláricos; Coartem | **Sim**  **1** | **Não**  **0** | PAL4 |
| Prescreve comprimidos de zinco | **Sim**  **1** | **Não**  **0** | PZN4 |  | Prescreve SRO | **Sim**  **1** | **Não**  **0** | PORS4 |
| Recomenda dose de AL / Coartem - 3 dias | **Sim**  **1** | **Não**  **0** | DAL4 |  | Recomenda dose de amoxicilina - 5 dias | **Sim**  **1** | **Não**  **0** | DAMO4 |
| Recomenda dose de SRO- quantas vezes for necessário | **Sim**  **1** | **Não**  **0** | DORS4 |  | Recomenda dose de zinco - 10 dias | **Sim**  **1** | **Não**  **0** | DZN4 |
| Dá primeira dose de amoxicilina | **Sim**  **1** | **Não**  **0** | FAMO4 |  | Dá primeira dose da AL / Coartem | **Sim**  **1** | **Não**  **0** | FAL4 |
| Dá primeira dose de SRO | **Sim**  **1** | **Não**  **0** | FORS4 |  | Dá dose de rectal artesunato | **Sim**  **1** | **Não**  **0** | FRA4 |
| Dá primeira dose de Zinco | **Sim**  **1** | **Não**  **0** | FZN4 |  | Criança não precisa de nenhum tratamento do APE | **Sim**  **1** | **Não**  **0** | WELL4 |
| **[REFERRAL]** |  |  |  |  |  |  |  |  |
| Referiu à unidade de sanitária | **Sim**  **1** | **Não**  **0** | REF4 |  | Passou uma guia de transferência | **Sim**  **1** | **Não**  **0** | NOTE4 |

**[ADVICE AND VISIT RECORDING]**

| Aconselha a utilização de Rede Mosquiteira Tratada na criança | **Sim**  **1** | **Não**  **0** | AITN4 |  | Aconselha a continuar a alimentar e a dar líquidos na criança | **Sim**  **1** | **Não**  **0** | AFLU4 |
| --- | --- | --- | --- | --- | --- | --- | --- | --- |
| Marca uma visita de seguimento | **Sim**  **1** | **Não**  **0** | FOL4 |  | Aconselha para voltar ou levar a criança para unidade sanitária se agravar | **Sim**  **1** | **Não**  **0** | AWRS4 |
| Regista a visita no livro de Consultas | **Sim**  **1** | **Não**  **0** | VREC4 |  | Verifica o estado de saúde de criança ou o cartão de vacinas | **Sim**  **1** | **Não**  **0** | CREC4 |

HISTÓRIA 5: LUCAS

| **CARTÃO 7: Você encontra se com Lucas quando está a fazer visitas domiciliárias, na sua aldeia. Ele tem 4 anos e sua mãe está preocupada porque ele tem estado tossindo por dois dias e tem dificuldade para respirar. Ele também teve uma febre baixa por dois ou três dias. Ela lhe pede para examiná-lo. Ao tocar sentes que ele está quente. Você verifica sua frequência respiratória e é 32 respirações por minuto. O resultado do TDR é negativo. Não há outros problemas.** Diga me primeiro - O que acha que tem o Lucas? **E,**Como vais controlar a doença da Lucas, incluindo qualquer tipo de tratamento ou conselho que você daria? |
| --- |

**[NÃO LER as opções abaixo para o APE e NÃO SUGERIR.**

**Se o APE menciona espontaneamente qualquer das seguintes acções na lista, faça um círculo no 1=SIM ao lado da acção. Quando ele/a terminar pergunte: “É tudo?” e continue a perguntar até que não tenha mais respostas para dar. Quando tiver terminado, faça círculos nas restantes que NÃO foram mencionadas como 0=NÃO].**

**[DIAGNOSIS]**

| 7.11.1 Identifica a criança como tendo frequência respiratório alta/ pneumonia | **Sim**  **1** | **Não**  **0** | FASB5 |  | 7.11.2 Identifica a criança como tendo diarreia / malaria | **Sim**  **1** | **Não**  **0** | MALD5 |
| --- | --- | --- | --- | --- | --- | --- | --- | --- |
| 7.11.3 Identifica a criança como tendo sinal de perigo / sintomas para referir ou uma doença grave (qualquer) | **Sim**  **1** | **Não**  **0** | SEV5 |  | 7.11.4 A criança não tem nenhum problema grave | **Sim**  **1** | **Não**  **0** | DIAR5 |
| **[TREATMENT]** |  |  |  |  |  |  |  |  |
| Prescreve antibióticos; amoxicilina | **Sim**  **1** | **Não**  **0** | PAMO5 |  | Prescreve anti-maláricos; Coartem | **Sim**  **1** | **Não**  **0** | PAL5 |
| Prescreve comprimidos de zinco | **Sim**  **1** | **Não**  **0** | PZN4 |  | Prescreve SRO | **Sim**  **1** | **Não**  **0** | PORS5 |
| Advises dose of AL/ Coartem – 3 days | **Sim**  **1** | **Não**  **0** | DAL5 |  | Recomenda dose de amoxicilina - 5 dias | **Sim**  **1** | **Não**  **0** | DAMO5 |
| Recomenda dose de SRO- quantas vezes for necessário | **Sim**  **1** | **Não**  **0** | DORS5 |  | Recomenda dose de zinco - 10 dias | **Sim**  **1** | **Não**  **0** | DZN5 |
| Dá primeira dose de amoxicilina | **Sim**  **1** | **Não**  **0** | FAMO5 |  | Dá primeira dose da AL / Coartem | **Sim**  **1** | **Não**  **0** | FAL5 |
| Dá primeira dose de SRO | **Sim**  **1** | **Não**  **0** | FORS5 |  | Dá dose de rectal artesunato / | **Sim**  **1** | **Não**  **0** | FRA5 |
| Dá primeira dose de Zinco | **Sim**  **1** | **Não**  **0** | FZN5 |  | Criança não precisa de nenhum tratamento do APE | **Sim**  **1** | **Não**  **0** | WELL5 |
| **[REFERRAL]** |  |  |  |  |  |  |  |  |
| Referiu à unidade de sanitária | **Sim**  **1** | **Não**  **0** | REF5 |  | Passou uma guia de transferência | **Sim**  **1** | **Não**  **0** | NOTE5 |
| **[ADVICE AND VISIT RECORDING]** |  |  |  |  |  |  |  |  |
| Aconselha a utilização de Rede Mosquiteira Tratada na criança | **Sim**  **1** | **Não**  **0** | AITN5 |  | Aconselha a continuar a alimentar e a dar líquidos na criança | **Sim**  **1** | **Não**  **0** | AFLU5 |
| Marca uma visita de seguimento | **Sim**  **1** | **Não**  **0** | FOL5 |  | Aconselha para voltar ou levar a criança para unidade sanitária se agravar | **Sim**  **1** | **Não**  **0** | AWRS5 |
| Regista a visitas no livro de Consultas | **Sim**  **1** | **Não**  **0** | VREC5 |  | Verifica o estado de saúde de criança ou o cartão de vacinas | **Sim**  **1** | **Não**  **0** | CREC5 |

#

# Parte 7 : Observação

| **SECÇÃO FINAL: As seguintes perguntas podem ser concluída em sua maioria por você observando as instalações, o uso doméstico os materiais de construção doméstico.(nota: considerar as alterações feitas no HH form na parte 4)** |
| --- |

| **[Observe ou peça:]** Que tipo de instalações sanitárias os agradados familiares fizeram para o uso doméstico? | | | | |
| --- | --- | --- | --- | --- |
| 1 | Autoclismo (tanque pia) | 4 | Latrina (cova) | STOILET |
| 2 | Latrina VIP | 5 | Banheiro público / Comum |  |
| 3 | Latrina melhorada | 6 | Nenhuma instalação (mato / campo) |  |
| 88 | Outro [ESPECIFIQUE ABAIXO |  |  |  |
| *Se ‘Outro’ Especifique*__________________________________________________________________________ | | | | STOILETO |

| **[Observe:]** Qual é o PRINCIPAL material que o telhado da casa é feito [Se mais de um material, a maioria do material usado no telhado**]** | | | | |
| --- | --- | --- | --- | --- |
| 1 | **NATURAL:** capim palha | 6 | **TERMINADO:** amianto (*lusalite*) | SEROOF |
| 2 | **NATURAL:** Lama / argila | 7 | **TERMINADO:** telhas |  |
| 3 | **NATURAL:** Papiro / folha de bananeira | 8 | **TERMINADO: Estanho (lata)** |  |
| 4 | **TERMINADO:** tabuas de madeira | 9 | **TERMINADO:** cimento |  |
| 5 | **TERMINADO:** Chapas / zinco / alumínio Ferro | 88 | Outro [ESPECIFIQUE ABAIXO] |  |
| *Se ‘Outro’ especifique___________________________________________________________________________* | | | | SROOFO |

| **[Observe:]** Qual é o PRINCIPAL material utilizado nas paredes da sua casa? [Se mais de um material, a maioria do material usado nas paredes] | | | | |
| --- | --- | --- | --- | --- |
| 1 | **NATURAL:** Caniço | 6 | **TERMINADO:** Cimento não rebocado | SWALLS |
| 2 | **NATURAL:** Palha | 7 | **TERMINADO:** Cimento rebocado |  |
| 3 | **RUDIMENTAR:** Lama | 8 | **TERMINADO:** Pedras |  |
| 4 | **RUDIMENTAR:** Tijolos (não queimado) | 9 | **TERMINADO:** Madeira |  |
| 5 | **TERMINADO:** Tijolos (queimado) | 88 | Outro [ESPECIFIQUE ABAIXO] |  |
| *Se ‘Outro’ especifique ___________________________________________________________________________* | | | | SWALLSO |

| **Observe:]** Qual é o PRINCIPAL material utilizado no piso da sua casa? [Se mais de um material, a maioria do material usado no piso**]** | | | | |
| --- | --- | --- | --- | --- |
| 1 | **NATURAL:** Areia / cascalho | 4 | **TERMINADO:** Madeira / tábuas | SFLOORS |
| 2 | **NATURAL:** Apenas Terra | 5 | **TERMINADO:** Cimento |  |
| 3 | **NATURAL:** Terra misturado com estrume de vaca | 88 | Outro [ESPECIFIQUE ABAIXO] |  |
| *Se ‘Outro’ especifique __________________________________________________________________________* | | | | SFLOORSO |

**Usar o seu GPS para identificar as coordenadas do agregado familiar do APE**

| Coordenadas do Agregado: EASTING | |  |  |  |  |  |  |  | “E | ECORDV |
| --- | --- | --- | --- | --- | --- | --- | --- | --- | --- | --- |
| Coordenadas do Agregado: NORTHING |  |  |  |  |  |  |  |  | “N | NCORDV |

**Fim do formulário do APE. Verifique o seu formulário e agradeça o APE.**

| **Manejo Integrado de Casos na Comunidade de Doenças na Infância em Moçambique**  INSCALE ENDLINE  QUESTIONÁRIO PARA AGREGADO FAMILIAR |  | [CÓDIGO DO FORMULÁRIO COLOQUE A ETIQUETA AQUI] | FORMNO |
| --- | --- | --- | --- |

PREENCHA ESTE FORMULÁRIO **UMA VEZ** PARA CADA AGREGADO FAMILIAR ONDE HÁ CRIANÇAS MENORES DE CINCO ANOS DE IDADE [NO BLANKS ALLOWED OTHER THAN SKIPS]

# Parte 1: Informações da entrevista

| Nome do Distrito | | | | | |
| --- | --- | --- | --- | --- | --- |
|  | 1. Funhalouro | 2. Govuro | 3. Homoine | 4. Inharrime | DISTRICT |
|  | 5. Inhassouro | 6. Jangamo | 7. Mabote | 8. Massinga |  |
|  | 9. Morrumbene | 10. Panda | 11. Vilanculos | 12. Zavala |  |

| Nome da Localidade |  | LOC |
| --- | --- | --- |

| Área de Enumeração |  |  |  | EA |
| --- | --- | --- | --- | --- |

| Nome da Vila |  | VILLAGE |
| --- | --- | --- |

| Código da Inquiridor |  |  | ICODE |
| --- | --- | --- | --- |

| Data da Entrevista (DDMM20AA) |  |  |  |  | 20 |  |  | IDATE |
| --- | --- | --- | --- | --- | --- | --- | --- | --- |

| O consentimento informado escrito foi fornecido? **[O consentimento informado é OBRIGATÓRIO para cada formulário preenchido – por favor faça um círculo para mostrar que você leu o consentimento e o entrevistado aceitou]** | | | |
| --- | --- | --- | --- |
| Sim | 1 |  | CONSENT |

| Código do Agregado Familiar **[Copie a partir da lista]** |  |  |  |  | **E** |  |  |  | · |  |  |  | HHID |
| --- | --- | --- | --- | --- | --- | --- | --- | --- | --- | --- | --- | --- | --- |

# Parte 2: Situação socio-económica e demográfica

# Esta secção é para a mãe ou responsável de crianças menores de 5 anos neste agregado familiar.

| Qual é o seu nome [Nome oficial] |  | RSPNAME |
| --- | --- | --- |

| Tem outro nome pelo qual as pessoas na comunidade o/a conhecem? [nome pelo qual é mais conhecido] |  | | | | RSPNICK | |
| --- | --- | --- | --- | --- | --- | --- |
| **[Observar o sexo do/a entrevistado/a]** | | | | | |  |
|  | | 1. Masculino | 2. Feminino |  | RSPSEX |  |

| Em que ano nasceu? **[AAAA]** [YEAR – BUT CAN TAKE VALUE 9999] | | | **Não Sabe=9999**  **Can chage on phone to 2020** | | |  |  | |  |  | | RSPYOB | |
| --- | --- | --- | --- | --- | --- | --- | --- | --- | --- | --- | --- | --- | --- |
| Em que mês nasceu? | | | | | | | | | | | | |  |
| 1  Janeiro | 2  Fevereiro | 3  Março | | 4  Abril | 5  Maio | | | 6  Junho | | | RSPMOB | |  |
| 7  Julho | 8  Agosto | 9  Setembro | | 10  Outubro | 11  Novembro | | | 12  Dezembro | | |  |  |  |
| 99  Não Sabe |  |  | |  |  | | |  | | |  | |  |

| Qual é a sua língua materna? | | | | | |  |
| --- | --- | --- | --- | --- | --- | --- |
| 1  Bitonga | 2  Chitsua | 3  ChiChopi | 4  Outra [ESPECIFIQUE ABAIXO] | | RSPLANG |  |
| *Se ‘Outra’ Especifique aqui____________________________________________*___________________________ | | | | RSPLANGO | | |

| Qual é a sua religião? | | | | | |  |
| --- | --- | --- | --- | --- | --- | --- |
| 1  Cristã | 2  Muçulmana | 3  Não tem religião | 88  Outra [ESPECIFIQUE ABAIXO] | | RSPFAITH |  |
| *Se ‘Outra’ Especifique aqui____________________________________________*___________________________ | | | | RSPFAITHO | | |

| Qual é o nível de educação mais alto que concluiu? **[responder mesmo se o entrevistado ainda estiver a estudar]** | | | | |
| --- | --- | --- | --- | --- |
| 1  Nenhuma | 2  Primário - Incompleto | 3  Primária – 2º Ciclo | 4  Secundário | RSPED |
| 5  Pré-Universitário | 6  Técnico Profissional | 7  Técnico Vocacional | 8  Nível Universitário |  |

| Você está actualmente solteiro, casado, vivendo com um parceiro, viúvo, divorciado ou separado? | | | | |
| --- | --- | --- | --- | --- |
| 1  Casado | 2  Maritalmente/ União de facto | 3  Viúvo | 4  Divorciado | MARRIED |
| 5  Separado | 6  Solteiro |  | |  |

| Qual é a sua principal ocupação? | | | | | |
| --- | --- | --- | --- | --- | --- |
| 1 | Funcionário do Sector Público: profissional / semi-qualificado | 6 | Trabalhadores por conta própria: comerciante / motorista de táxi / pequeno negócio | | RSPOCCPM |
| 2 | Funcionário do Sector Público: manual/trabalhador | 7 | Trabalhadores por conta própria: agricultor/pescador | |  |
| 3 | Funcionário privado /ONG: profissional / semi-qualificado | 8 | Doméstica | |  |
| 4 | Funcionário privado /ONG: manual/trabalhador | 9 | Desempregado | |  |
| 5 | Trabalhador ocasional ou diário | 10 | Doente/ Incapaz de trabalhar, deficiente | |  |
| **Se as opções forem 1-5, vá para 2.11]** | | 11 | Estudante | |  |
|  |  | **[Se as opções de 6-11, vá para 2.12]** | | |  |
| 88 | Outra |  |  |  |  |
| *Se ‘Outra especifique______________________________________________*_______________ | | | | **[Se as opções forem 6-11, vá a 2.12]** | RSPOCUPO |

| **Se na pergunta 2.10 as opções foram 1-5, pergunte:]** Quanto é que você recebeu no último mês em que trabalhou? | | | | |
| --- | --- | --- | --- | --- |
| 1  MTN: 0- <1000,00 | 2  MTN: 1000,00 - <3.400,00 | 3  MTN: 3400,00 - <5.200,00 | 4  MTN: 5.200,00 ou mais | RSPMONTH |

| Quantos membros da sua família estão actualmente a trabalhar no total (incluindo você)? [NO BLANKS ALLOWED] |  |  | MHHWORK |
| --- | --- | --- | --- |

| Qual é a renda média mensal em dinheiro de todos os salários / vencimentos / produzidos no seu agregado familiar?**[se necessário, permitir que ao inquirido para verificar com chefe de família / outros membros]** | | | | |
| --- | --- | --- | --- | --- |
| 1  MTN: 0.0/mês | 2  MTN: 1 - < 1000,00/mês | 3  MTN: 1000,00-< 2.500,00 | 4  MTN:2.500,00-< 3.200,00 | HHINCOME |
| 5  MTN: 3.200,00-< 5.200,00 | 6  MTN:5.200,00-< 10.000,00 | 7  MTN: 10.000,00 ou mais por mês | 99  Não sabe |  |

| Será que algum dos membros do agregado familiar possui um dos seguintes meios de transporte? [Leia a lista e faça um círculo no sim ou não] | | | | | |
| --- | --- | --- | --- | --- | --- |
|  | Um carrinho de tracção animal | 1. SIM | 0. NÃO |  | CART |
|  | Bicicleta | 1. SIM | 0. NÃO |  | BICYCLE |
|  | Uma mota | 1. SIM | 0. NÃO |  | SCOOTE |
|  | Um carro/camião | 1. SIM | 0. NÃO |  | CAR |
|  | Um barco/canoa sem motor | 1. SIM | 0. NÃO |  | BOAT |
|  | Um barco.canoa a motor | 1. SIM | 0. NÃO |  | MBOAT |
| Outro [especifique] ______________________________________________ | |  | | | OTRPO |

| Qual é a PRINCIPAL fonte de iluminação para a casa? | | | | | |  |
| --- | --- | --- | --- | --- | --- | --- |
| 1  Candeeiro de querosene / parafina / petróleo | 2  Vela de parafina | 3  Lenha | 4  Vela (Cera) | | HHLIGHT |  |
| 5  Eletricidade | 6  Lâmpada solar | 7Lanterna | 88  Outra [ESPECIFIQUE ABAIXO] | |  |  |
| *Se ‘Outro’ especifique ___________________________________________*___________________________ | | | | HHLIGHTO | | |

| O seu agregado familiar tem ALGUM dos seguintes elementos a funcionar bem? **[Leia a lista e faça um círculo sim / não ]** | | | | | |
| --- | --- | --- | --- | --- | --- |
|  | Eletricidade (linha fixa) | 1. SIM | 0. NÃO |  | ELECTR |
|  | Energia solar / gerador | 1. SIM | 0. NÃO |  | SOLAR |
|  | Rádio | 1. SIM | 0. NÃO |  | RADIO |
|  | A televisão | 1. SIM | 0. NÃO |  | TELVI |
|  | Um telemóvel | 1. SIM | 0. NÃO |  | MPHONE |
|  | Um telefone fixo | 1. SIM | 0. NÃO |  | FPHONE |
|  | Uma geleira | 1. SIM | 0. NÃO |  | REFRIG |
|  | Uma cama com uma base, por exemplo, feito de madeira, ferro | 1. SIM | 0. NÃO |  | BED |
|  | Um colchão de espuma | 1. SIM | 0. NÃO |  | MATRESS |
|  | Um sofá | 1. SIM | 0. NÃO |  | SOFA |
|  | Roupeiro (para vestuário) | 1. SIM | 0. NÃO |  | CUPBOARD |
|  | Um relógio | 1. SIM | 0. NÃO |  | CLOCK |
|  | Uma rede mosquiteira que pode ser usado enquanto dorme | 1. SIM | 0. NÃO |  | NET |

| O seu filho mais novo dormiu na última noite debaixo duma rede mosquiteira? | | | |
| --- | --- | --- | --- |
| Sim | 1 |  | CHILDNET |
| Não | 0 |  |  |
| Não Sabe | 99 |  |  |
| Não é aplicável – não possui uma rede | 77 |  |  |

| No total, quantas crianças com idade inferior a 5 anos dormiram debaixo de uma rede mosquiteira na noite passada? **Não Sabe=99, Não se aplica/não tem rede=77 [NO BLANKS – but CAN TAKE VALUE 77** |  |  | NUMNET |
| --- | --- | --- | --- |

| **Será que algum membro do seu agregado familiar possui alguns dos seguintes animais?**  [**Não incluir gatos ou cães]: [NO BLANKS ALLOWED, BUT CAN BE 999]**  **[Preencha 999 se agregado familiar possui um tipo de animal, mas o número de animais é desconhecido]** | | | | | | | |
| --- | --- | --- | --- | --- | --- | --- | --- |
|  | Bois |  |  |  |  |  | COWS |
|  | Cavalo, burro ou mula |  |  |  |  |  | HORSES |
|  | Cabrito |  |  |  |  |  | GOATS |
|  | Ovelha |  |  |  |  |  | SHEEP |
|  | Porco |  |  |  |  |  | PIGS |
|  | Galinha, Patos, Peru ou Coelho |  |  |  |  |  | CHDUTURA |
|  | Colmeias (em uso) |  |  |  |  |  | BEEHIVES |
|  | Outro [ESPECIFIQUE ABAIXO] |  |  |  |  |  | ANIMALO |
| *Se ‘Outro’ especifique ________________________________________________*__________ | | | | | | | ANIMO |

| Será que algum membro do seu agregado familiar tem a sua própria terra? | | | |
| --- | --- | --- | --- |
| Sim | 1 |  | HHLAND |
| Não | 0 | **🡪2.24** |  |

| Qual a quantidade de terra que os membros da sua família possuem? [NO BLANKS unless skipped – BUT CAN BE 999,99] |  |  |  |  |  | · |  |  | hectares | HHACRES |
| --- | --- | --- | --- | --- | --- | --- | --- | --- | --- | --- |
| **[Use o seu conversor, se a medida não é dada em hectares. Faça o seu cálculo na íntegra neste formulário e mostrar o seu supervisor para confirmação]** | **Exemplo; se a resposta for um e meio de hectares, preencha 001,50; se a resposta for de três hectares, preencher 003,00;**  **Não sei = 999,99** | | | | | | | | | |

| Da terra que você possui, qual a dimensão dos que são terrenos agrícolas? **Não Sabe=999.99** |  |  |  |  |  | · |  |  | hectares | HHAGRIC |
| --- | --- | --- | --- | --- | --- | --- | --- | --- | --- | --- |

| Quanta terra que você possui é para outros fins, como para negócios ou aluguer? **Não Sabe =999.99** |  |  |  |  | · |  |  | hectares | HHOP |
| --- | --- | --- | --- | --- | --- | --- | --- | --- | --- |

| Qual é o PRINCIPAL tipo de combustível utilizado na preparação dos alimentos? | | | | | |  |
| --- | --- | --- | --- | --- | --- | --- |
| 1  Lenha | 2  Carvão | 3  Parafina / Querosene/ petróleo | 4  Gás | | HHFUEL |  |
| 5  Eletricidade | 6  Resíduos /palha /capim | 7  Excremento de animais | 8  Energia solar | |  |  |
| 9  Nenhum alimento é preparado na casa | 88  Outro [ESPECIFIQUE ABAIXO] |  | | |  |  |
| *Se ‘Outro’ especifique aqui ____________________________________________*___________________________ | | | | HHFUELO | | |

| Qual é a PRINCIPAL fonte de água potável para os membros do seu agregado familiar? | | | | | |  |
| --- | --- | --- | --- | --- | --- | --- |
| 1  Água canalizada para casa | 2  Torneira/ Fontenário público | 3  Poço desprotegido | 4  Poço protegido | | MWATER |  |
| 5  Fonte de água desprotegido | 6  Fonte de água protegido | 7  Furo | 8  Rio / Riacho | |  |  |
| 9  Lagoa/lago | 10  Recolha de águas pluviais | 11  Compra de água / camião | 88  Outro [ESPECIFIQUE ABAIXO] | |  |  |
| *Se ‘Outro’ especifique aqui_____________________________________________*___________________________ | | | | MWATERO | | |

# Parte 3: Conhecimento sobre o programa dos APEs e visitas domiciliares

| A sua casa foi visitada por um APE no último mês? | | | |
| --- | --- | --- | --- |
| Sim | 1 |  | VHTMO |
| Não | 0 | **🡪3.4** |  |
| Eu não sei quem é o APE na minha comunidade | 99 |  |  |

| O quê que o APE fez durante a visita? [NÃO SUGERIR = Faca círculo nas opções que o dono da casa menciona. Se o entrevistado diz que não se lembra o que o fez o APE, escrever na linha de 'outro'] | | | | |
| --- | --- | --- | --- | --- |
| 3.2.1 Pergunta sobre vacinações dos meus filhos | 1. MENCIONADO | 0. NÃO MENCIONADO |  | VHTVAC |
| Manteve registo de saúde da minha família | 1. MENCIONADO | 0. NÃO MENCIONADO |  | KEEPREC |
| Deu conselhos sobre hábitos de saúde | 1. MENCIONADO | 0. NÃO MENCIONADO |  | VHTADV |
| Perguntou ou observou o estado de saúde, as práticas de saúde, e / ou higiene dos membros do agregado familiar | 1. MENCIONADO | 0. NÃO MENCIONADO |  | VHTOB |
| Tratou um membro da família que estava doente | 1. MENCIONADO | 0. NÃO MENCIONADO |  | VHTREAT |
| Outro [Especifique] ____________________________________________________________________ | | | | VHTOTO |

| E na visita você notou se o APE tinha ou usou qualquer um dos seguintes: [**Leia as todas as opções**] | | | | |
| --- | --- | --- | --- | --- |
| Uma lâmpada de energia solar? **[Mostra a imagem se possível** | 1. SIM | 0. NÃO |  | VHTLAMP |
| Um telemóvel do APE, por exemplo, para verificar a existência de sintomas ou ajudar a contar as respirações de uma criança doente? | 1. SIM | 0. NÃO |  | VHTMOB |

## **DIGA:**

Em algumas comunidades, os APEs receberam telefones e carregadores solares para ajudá-los no seu trabalho. Às vezes, os membros da comunidade como você, podem pagar ao APE para carregar os seus telefones usando o carregador solar do APE.

| Você sabe que o APE desta comunidade tem um carregador solar que você pode pagar para usar para carregar seu telefone? | | | |
| --- | --- | --- | --- |
| Sim, eles têm um carregador solar | 1 |  | VHTKNWCH |
| Não, eles estão sem um carregador solar | 0 | **🡪**S4 |  |
| Não tenho certeza se eles fazem ou não | 99 |  |  |

| No último mês, quantas vezes você ou membros de sua família usou o carregador solar do APE para carregar seus telefones? **[00-nenhuma, 77-** **Não aplicável, pois entrevistado ou a família não tem telefone]** |  |  | VHTCHRG |
| --- | --- | --- | --- |

# Parte 4: Observação

| **As seguintes perguntas, podem ser concluídas na sua maioria por você, observando as instalações de uso doméstico e os materiais de construção.** |
| --- |

| **[Observe ou peça:]** Que tipo de instalações sanitárias os agregados familiares fizeram para o uso doméstico? | | | | |
| --- | --- | --- | --- | --- |
| 1 | Autoclismo (tanque pia) | 4 | Latrina (cova) | STOILET |
| 2 | Latrina VIP | 5 | Banheiro público / Comum |  |
| 3 | Latrina melhorada | 6 | Nenhuma instalação (mato / campo) |  |
| 88 | Outro [ESPECIFIQUE ABAIXO |  |  |  |
| *Se ‘Outro’ Especifique_____________________________________________*_______________________________ | | | | STOILETO |

| **[Observe:]** Qual é o PRINCIPAL material que o telhado da casa é feito [Se mais de um material, a **maioria** do material usado no telhado] | | | | |
| --- | --- | --- | --- | --- |
| 1 | **NATURAL:** capim palha | 6 | **TERMINADO:** amianto (lusalite) | SEROOF |
| 2 | **NATURAL:** Lama / argila | 7 | **TERMINADO:** telhas |  |
| 3 | **NATURAL:** Papiro / folha de bananeira | 8 | **TERMINADO: Estanho (lata)** |  |
| 4 | **TERMINADO:** tabuas de madeira | 9 | **TERMINADO:** cimento |  |
| 5 | **TERMINADO:** Chapas / zinco / alumínio Ferro | 88 | Outro [ESPECIFIQUE ABAIXO] |  |
| *Se ‘Outro’ especifique____________________________________________*_______________________________ | | | | SROOFO |

| **[Observe:]** Qual é o PRINCIPAL material utilizado nas paredes da sua casa? [Se mais de um material, a **maioria** do material usado nas paredes] | | | | |  |
| --- | --- | --- | --- | --- | --- |
| 1 | **NATURAL:** Caniço | 6 | **TERMINADO:** Cimento não rebocado | SWALLS |  |
| 2 | **NATURAL:** Palha | 7 | **TERMINADO:** Cimento rebocado |  |  |
| 3 | **RUDIMENTAR:** Lama | 8 | **TERMINADO:** Pedras |  |  |
| 4 | **RUDIMENTAR:** Tijolos (não queimado) | 9 | **TERMINADO:** Madeira |  |  |
| 5 | **TERMINADO:** Tijolos (queimado) | 88 | Outro [ESPECIFIQUE ABAIXO] |  |  |
| *Se ‘Outro’ especifique ____________________________________________*_______________________________ | | | | SWALLSO |  |
|  | | | |  |  |
| **[Observe:]** Qual é o PRINCIPAL material utilizado no chão da sua casa? [Se mais de um material, a **maioria** do material usado no chão] | | | | | |
| 1 | **NATURAL:** Areia / cascalho | 4 | **TERMINADO:** Madeira / tábuas | SFLOORS | |
| 2 | **NATURAL:** Apenas Terra | 5 | **TERMINADO:** Cimento |  |  |
| 3 | **NATURAL:** Terra misturado com estrume de vaca | 88 | Outro [ESPECIFIQUE ABAIXO] |  |  |
| *Se ‘Outro’ especifique ___________________________________________*_______________________________ | | | | SFLOORSO | |

**Usar o seu GPS para identificar as coordenadas do agregado familiar**

| Coordenadas do Agregado: EASTING | |  |  |  |  |  |  |  | “E | ECORDH |
| --- | --- | --- | --- | --- | --- | --- | --- | --- | --- | --- |
| Coordenadas do Agregado: NORTHING |  |  |  |  |  |  |  |  | “N | NCORDH |

# Parte 5: Crianças menores de 5 anos de idade

# *Reconfirme que o entrevistado é o responsável principal das crianças neste agregado familiar.*

# DIGA:

Agora vou fazer-lhe algumas perguntas sobre as crianças menores de cinco anos de idade que estão sob seus cuidados neste agregado familiar e dormiram aqui na noite passada.

| Quantas crianças com idade inferior a cinco anos, que dormiram aqui na noite passada, fazem parte deste agregado familiar? **[**Certifique-se **que o número de crianças não se limita a apenas a crianças do agregado familiar)]** |  |  | NUMCHILD |
| --- | --- | --- | --- |

**DIGA:**

**Agora irei fazer algumas perguntas sobre cada uma das crianças menores de cinco anos de idade**

**[-** Pega **o primeiro “Formulários ID da Criança” que não esta preenchido , e coloque o código do agregado familiar no topo deste formulário.**

**-** **Verifique se preencheu o número total dos Formulários ID da Criança, e os códigos das crianças escritos em cada um desses formulários, e que esse numero total corresponde com o número de crianças inscritas no 5.1 acima] ______________________________________________________________________________________________________________**

**FIM DO FORMULARIO DO AGREGADO FAMILIAR**

**VERIFIQUE O SEU FORMULÁRIO E AGRADEÇA AO ENTREVISTADO**

| **Manejo integrado de casos na comunidade de doenças da infância, em Inhambane**  INSCALE ENDLINE **QUESTIONÁRIO ID DA CRIANÇA** |  | [FORM ID COPIED FROM HOUSEHOLD FORM] | FORMNO |
| --- | --- | --- | --- |

# Características demográficas

PREENCHA O FORMULÁRIO PARA TODA CRIANÇA MENOR DE 5 ANOS QUE VIVE NESTE AGREGADO

**[Preencha os dados abaixo 1.1 and 1.2 – Copie o código do agregado familiar do formulário do Agregado Familiar que acabou de preencher.**

| Código do AF |  |  |  |  | **E** |  |  |  | · |  |  |  | HHID |
| --- | --- | --- | --- | --- | --- | --- | --- | --- | --- | --- | --- | --- | --- |

| Código da Criança **[Atribua a cada criança um número único entre 01-99]** |  |  | CNO |
| --- | --- | --- | --- |

| Como se chama a criança? |  | CNAME |
| --- | --- | --- |

| O/A [NOME] tem menos de 1 mês? | | | |
| --- | --- | --- | --- |
| Sim | 1 | 🡪**1.6** | CLESS5 |
| Não | 0 |  |  |

| Qual é a idade do [NOME]? **[Escreva a idade em anos e meses]** | | | |
| --- | --- | --- | --- |
| ANOS **[=00 Se menor de 1 ano]** |  |  | ANOS |
| MESES |  |  | MESES |

| Qual é a data de nascimento do [NOME]? **[DD/MM/20YY]** |  |  | / |  |  | 20 |  |  | CDOB |
| --- | --- | --- | --- | --- | --- | --- | --- | --- | --- |

| **[Todos os questionários devem ter a data completa de nascimento da criança (Não existe a opção ‘Desconhecida). [Se o responsável pela criança não tiver certeza, explore com profundidade para obter melhor estimativa]** |
| --- |

| O [NOME] é menino ou menina? | | | | |
| --- | --- | --- | --- | --- |
| 1  menino | 2  menina |  |  | CSEX |

| Qual é a relação de parentesco com [NOME] | | | | | | | | | |
| --- | --- | --- | --- | --- | --- | --- | --- | --- | --- |
| 1  Mãe | 2  Pai | 3  Irmão/Irmã | | 4  Tia/Tio | | 5  Avó/Avô | | CRELATE | |
| 88  Outro [ESPECIFIQUE ABAIXO] |  | |  | |  | | |  |  |
| *Se ‘Outro’ especifique aqui ________________________________________________*___________________________ | | | | | | | CRELATEO | |  |

| **Diga:**  Agora gostaria de saber se [NOME] esteve doente no último mês, incluindo hoje? |
| --- |

| No último mês, incluindo o dia de hoje [NOME] adoeceu? **[LEIA EM VOZ ALTA TODAS AS OPÇÕES]** **[SE A CRIANÇA TIVER ADOECIDO MAIS DE UMA VEZ NO ÚLTIMO MÊS, APENAS PESQUISE SOBRE A ÚLTIMA DOENÇA (A MAIS RECENTE) ]** | | | |
| --- | --- | --- | --- |
| SIM, nas últimas **2 semanas** | 1 |  | CESICK |
| SIM, há **mais de 2 semanas, mas até 1 mês atrás** | 2 |  |  |
| NÃO, a criança não adoeceu no último mês | 0 | **🡪FIM** |  |

| **-Se a criança tiver adoecido em qualquer momento do ultimo mês, PREENCHA O QUESTIONÁRIO DA CRIANÇA DOENTE, Copie os detalhes de identificação do**  **topo deste questionário para o questionário da criança doente.**  FIM: **-Se a criança não adoeceu no ultimo mês, TERMINE A ENTREVISTA PARA ESTA CRIANÇA E PASSE PARA A PRÓXIMA CRIANÇA.**  **Se não houver mais crianças nesta casa, termine a entrevista para este AGREGADO FAMILIAR**. |
| --- |

| **Manejo integrado de casos na comunidade de doenças da infância, em Inhambane**  INSCALE ENDLINE  **QUESTIONÁRIO DA CRIANÇA DOENTE** |  | [FORM ID COPIED FROM HOUSEHOLD FORM] | FORMNO |
| --- | --- | --- | --- |

# Informação Básica da Criança

PREENCHA O QUESTIONÁRIO PARA TODA A CRIANÇA QUE ESTEVE DOENTE NO ÚLTIMO MÊS **[Preencha os dados abaixo 1.1-1.3** **– consulte o questionário do agregado familiar e da criança que acabou de preencher para copiar o código do agregado familiar (AF), o código da criança e o nome**

| Código do AF **[Copie da Listagem]** |  |  |  |  | **E** |  |  |  | · |  |  |  | HHID |
| --- | --- | --- | --- | --- | --- | --- | --- | --- | --- | --- | --- | --- | --- |

| Código da Criança **[**copie este número de 1.2 (Código Criança) na primeira página do questionário da Criança] |  |  | CNO |
| --- | --- | --- | --- |

| **[Copie o nome da criança a partir do questionário da criança]** |  | CNAME |
| --- | --- | --- |

**DIGA:**

Agora gostaria de lhe fazer algumas perguntas sobre a última doença que o [NOME] teve **no último mês.**

# Doença

| Na última vez que [NOME] esteve doente, era uma doença com tosse? | | | |
| --- | --- | --- | --- |
| Sim | 1 |  | COUGH |
| Não | 0 | 🡪 2.4 |  |

| **Quando [NOME] teve a doença com tosse, tinha dificuldades em respirar ou respirava mais rápido que o habitual, com respirações curtas e rápidas?** | | | |
| --- | --- | --- | --- |
| Sim | 1 |  | BREATHE |
| Não | 0 | 🡪 2.4 |  |

| Quando [NOME] tinha dificuldade em respirar ou respirava rapidamente, era um problema com o peito ou com o nariz escorrendo ou entupido? **NÃO** | | | | |
| --- | --- | --- | --- | --- |
| 1  Problema com o Peito | 2  Nariz entupido ou escorrendo | 3  Ambos | 4  Não saberia dizer | LBREATHE |
| 0  Nenhum |  |  |  |  |

| Quando [NOME] estava doente, teve febre? | | | |
| --- | --- | --- | --- |
| Sim | 1 |  | CFEVER |
| Não | 0 |  |  |

| Da última vez que [NOME] esteve doente, teve diarreia? | | | |
| --- | --- | --- | --- |
| Sim | 1 |  | CDIARR |
| Não | 0 | 🡪 2.8 |  |

| No dia em que [NOME] esteve mais doente, quantas vezes defecou nesse dia e na noite seguinte? |  |  | CSTOOL |
| --- | --- | --- | --- |

| A diarreia era: [Explore **com profundidade]** | | | |
| --- | --- | --- | --- |
| Aguada | 1 |  | DIARRT |
| Tinha muco | 2 |  |  |
| Sangue | 3 |  |  |

| **Da última vez que [NOME] esteve doente,** teve os seguintes sintomas? **[Leia a lista e seleccione no sim ou não. Explique os sintomas se necessário]** | | | | | |
| --- | --- | --- | --- | --- | --- |
|  | Convulsões | 1. SIM | 0. NÃO |  | CONVUL |
|  | Inconsciência | 1. SIM | 0. NÃO |  | UNCONS |
|  | Letargia/Sono anormal | 1. SIM | 0. NÃO |  | SLEEPY |
|  | Vomitar tudo | 1. SIM | 0. NÃO |  | VOMT |
|  | Não beber/amamentação | 1. SIM | 0. NÃO |  | NODRINK |
|  | Perda de apetite | 1. SIM | 0. NÃO |  | NOEAT |
|  | Tiragem ***[Explique ‘todo peito para dentro]*** | 1. SIM | 0. NÃO |  | INDRAW |
|  | Dor de peito | 1. SIM | 0. NÃO |  | CHPAIN |
|  | Tosse com sangue | 1. SIM | 0. NÃO |  | CGBLOOD |
|  | Respiração ruidosa | 1. SIM | 0. NÃO |  | GRUNT |
|  | Sibilos | 1. SIM | 0. NÃO |  | WHEEZE |
|  | Falta de ar | 1. SIM | 0. NÃO |  | SHORTB |
|  | Narinas abrem por causa da respiração difícil | 1. SIM | 0. NÃO |  | FLARE |
|  | Tremores | 1. SIM | 0. NÃO |  | CHILLS |
|  | Inquietação | 1. SIM | 0. NÃO |  | RESTLS |
|  | Irritabilidade | 1. SIM | 0. NÃO |  | IRRITA |

# Procura por cuidados de saúde

**DIGA:**

**Agora, gostaria de lhe fazer algumas perguntas sobre a procura de atendimento fora de casa para a última doença que [NOME] teve. Se procurou por cuidados, eu também gostaria de saber onde ou de quem procurou atendimento, o custo e o tempo que levou.**

| Você procurou aconselhamento ou tratamento for a de casa para a última doença? | | | |
| --- | --- | --- | --- |
| Sim | 1 | 🡪3.3 | RXTHOME |
| Não | 0 |  |  |

| **Porquê não procurou aconselhamento ou tratamento fora de casa**? [**Seleccione na razão principal]** | | | | | | |
| --- | --- | --- | --- | --- | --- | --- |
| 1  Unidade Sanitária/Provedor de Saúde muito distante | 2  Falta de tempo | 3  Não quis ir à unidade sanitária | 4  Podia controlar a doença em casa. | **🡪S4** | NOERXT | |
| 5  Podia tratar a doença com os medicamentos que tinha em casa | 6  O esposo/esposa não deixou | 7  **Sogra**/minha mãe não deixou | 8  A doença não era grave/A doença passou por si |  |  |  |
| 88  Outro [ESPECIFIQUE ABAIXO] |  | | | |  | |
| *Se ‘Outro’ especifique ________________________________________________*__________ | | | | **🡪S4** | NOERXTO |  |

| Onde procurou aconselhamento ou tratamento primeiro? **[SE na UNIDADE SANITÁRIA: Pergunte pelo nome da US e verifique o nível da US na lista de US fornecida]** | | | | |
| --- | --- | --- | --- | --- |
| 1 | **SECTOR PÚBLICO:** APE | 8 | **SECTOR PRIVADO:** Farmácia | RXTPLACEU |
| 2 | **SECTOR PÚBLICO:** Posto de Saúde (PS) | 9 | **SECTOR PRIVADO:** Posto de saúde privado/pequena clinica |  |
| 3 | **SECTOR PÚBLICO:** US de Nível I (CS I) | 10 | **SECTOR PRIVADO:** Hospital privado |  |
| 4 | **SECTOR PÚBLICO:** US de Nível II (CS II) | 11 | **SECTOR PRIVADO:** Serviços móveis/ serviços de extensão |  |
| 5 | **SECTOR PÚBLICO:** US de Nível III (CS III) | 12 | **OUTRO:** Loja Geral |  |
| 6 | Hospital | 13 | **OUTRO:** Médico tradicional |  |
| 7 | **SECTOR PÚBLICO:** Serviços móveis/ serviços de extensão | 88 | Outro [ESPECIFIQUE ABAIXO] |  |
| *Se ‘Outro’ Especifique________________________________________________*______________________________ | | | | RXTPLACO |

| Quantos dias depois de notar que a criança estava doente procurou por ajuda fora de casa? | **DAYS** |  |  | HDAYS |
| --- | --- | --- | --- | --- |

| Como chegou ao primeiro lugar onde solicitou tratamento ou aconselhamento? **[Seleccione no transporte principal]** | | | | | |
| --- | --- | --- | --- | --- | --- |
| 1  A pé | 2  Bicicleta | 3  Txopela/Taxi de motorizada | 4  Táxi (Transporte público/carro) | GOTPLACE | |
| 5  Machimbombo | 6  Carro alugado | 7  Camião/carrinha | 8  Barco |  |  |
| 88  Outro [ESPECIFIQUE ABAIXO] |  | | |  | |
| *Se ‘Outro’ especifique ________________________________________________*__________ | | | | GOTPLACEO |  |

| Quanto tempo levou de casa para chegar ao primeiro sítio onde solicitou tratamento ou aconselhamento? **[**Preencher ambas as horas e minutos **ex: 45 mins = 00hrs, 45mins]** | | | |
| --- | --- | --- | --- |
| hrs |  |  | DURHRS |
| mins |  |  | MINS |

| Em relação à visita ao primeiro lugar onde procurou aconselhamento ou tratamento: Gastou dinheiro em qualquer um do seguinte: **[Exemplo 50 MT – 000050]**  **[Preencher 000000 se não tiver gasto dinheiro]**  **[Preencher 999999 se o valor é desconhecido]** | | | | | | | | | |
| --- | --- | --- | --- | --- | --- | --- | --- | --- | --- |
| Transporte | **Mts** |  |  |  |  |  |  |  | TRANS |
| Taxas de registo de pacientes | **Mts** |  |  |  |  |  |  |  | REFGEES |
| Custo de materiais (como luvas, Intravenoso, seringas, etc) | **Mts** |  |  |  |  |  |  |  | SYRINGES |
| Medicamentos | **Mts** |  |  |  |  |  |  |  | MEDICINE |
| Despesas de internamento | **Mts** |  |  |  |  |  |  |  | OVERNFEES |
| Presentes monetários / não monetários, contribuições | **Mts** |  |  |  |  |  |  |  | GIFTS |
| Custos de sustento (alimentos, bebidas) | **Mts** |  |  |  |  |  |  |  | SCOSTS |
| Outras despesas financeiras [ESPECIFIQUE ABAIXO] | **Mts** |  |  |  |  |  |  |  | OEXPENSE |
| *Se ‘Outro’ especifique ________________________________________________*__________ | | | | | | | | | OCOSTS |

| Quanto tempo gastou no primeiro local que procurou aconselhamento ou tratamento Por favor, inclua ambos tempos de espera e da consulta. **[**Preencher ambos horas e minutos **ex:. 45 mins = 00hrs, 45mins]** | | | |
| --- | --- | --- | --- |
| hrs |  |  | FACHRS |
| mins |  |  | FACMIN |

| Quantos ADULTOS, incluindo a si, acompanharam [NOME] para procurar aconselhamento/tratamento? **[Apenas você=01]** |  |  | NUMACC |
| --- | --- | --- | --- |

| Quando levou [NOME] ao primeiro local, foi lhe indicado outro sitio para aconselhamento/tratamento adicional? | | | |
| --- | --- | --- | --- |
| Sim | 1 |  | REFER1 |
| Não | 0 | **🡪 3.12** |  |

| **[Se SIM:]** Foi ao Segundo lugar que te foi indicado? | | | |
| --- | --- | --- | --- |
| Yes Sim | 1 | **🡪3.14** | GOREF1 |
| Não - procurei por aconselhamento ou tratamento em outro local diferente do que me foi indicado | 2 | **🡪3.13** |  |
| Não - procurei nenhum aconselhamento ou tratamento fora de casa | 0 | **🡪3.29** |  |

| **[Se a resposta de3.10 é NÃO:]** Mesmo que não tenha sido indicado, procurou aconselhamento ou tratamento em qualquer outro sítio depois de ir para o primeiro local? | | | |
| --- | --- | --- | --- |
| Sim | 1 |  | SEEK2 |
| Não | 0 | **🡪 3.29** |  |

| Porque razão procurou atendimento em outro lugar? **[Apenas seleccione na razão principal]** | | | |
| --- | --- | --- | --- |
| Falta de medicamentos no primeiro sítio | 1 |  | REAS2CS |
| Estava insatisfeito com o aconselhamento/ tratamento dado no primeiro sítio | 2 |  |  |
| Outro [ESPECIFIQUE ABAIXO | 88 |  |  |
| *Se ‘Outro’ especifique ________________________________________________*________________________________ | | | REAS2CS**O** |

| Qual foi o Segundo sítio onde procurou aconselhamento ou tratamento? **[SE NA UNIDADE SANITÁRIA: Pergunte pelo nome da US e verifique o nível da US na lista de US fornecida]** | | | | |
| --- | --- | --- | --- | --- |
| 1 | **SECTOR PÚBLICO:** APE | 8 | **SECTOR PRIVADO:** Farmácia | RXTPLAC2U |
| 2 | **SECTOR PÚBLICO:** Posto de Saúde (PS) | 9 | **SECTOR PRIVADO:** Posto de saúde privado/pequena clinica |  |
| 3 | **SECTOR PÚBLICO:** US de Nível I (CS I) | 10 | **SECTOR PRIVADO:** Hospital privado |  |
| 4 | **SECTOR PÚBLICO:** US de Nível II (CS II) | 11 | **SECTOR PRIVADO:** Serviços móveis/ serviços de extensão |  |
| 5 | **SECTOR PÚBLICO:** US de Nível III (CS III) | 12 | **OUTRO:** Loja Geral |  |
| 6 | Hospital | 13 | **OUTRO:** Médico tradicional |  |
| 7 | **SECTOR PÚBLICO:** Serviços móveis/ serviços de extensão | 88 | Outro [ESPECIFIQUE ABAIXO] |  |
| *Se‘Outro’ Especifique________________________________________________*______________________________ | | | | RXTPLACO2 |

| Como chegou ao segundo sitio onde solicitou tratamento ou aconselhamento? **[Seleccione no transporte principal]** | | | | | |
| --- | --- | --- | --- | --- | --- |
| 1  A pé | 2  Biclicleta | 3  Txopela/Taxi de motorizada | 4  Taxi (Transporte público/carro) | GOTPLAC2 | |
| 5  Machimbombo | 6  Carro alugado | 7  Camião/carrinha | 8  Barco |  |  |
| 88  Outro [ESPECIFIQUE ABAIXO] |  | | |  | |
| *Se ‘Outro’ especifique ________________________________________________*__________ | | | | GOTPLACO2 |  |

| Quanto tempo levou de casa para chegar ao segundo sítio onde solicitou tratamento ou aconselhamento? **[**Preencher ambos horas e minutos **Ex: 45 mins = 00hrs, 45mins]** | | | |
| --- | --- | --- | --- |
| hrs |  |  | DURHRS2 |
| mins |  |  | MINS2 |

| Em relação à visita ao segundo local onde procurou aconselhamento ou tratamento: Gastou dinheiro em qualquer um do seguinte: **[Exemplo 50 MT – 000050]**  **[Preencher 000000 se não tiver gasto dinheiro]**  **[Preencher 999999 se o valor é desconhecido]** | | | | | | | | | |
| --- | --- | --- | --- | --- | --- | --- | --- | --- | --- |
| Transporte | **Mts** |  |  |  |  |  |  |  | TRANS2 |
| Taxas de registo de pacientes | **Mts** |  |  |  |  |  |  |  | REFGEES2 |
| Custo de materiais (como luvas, IV, seringas, etc) | **Mts** |  |  |  |  |  |  |  | SYRINGES2 |
| Medicamentos | **Mts** |  |  |  |  |  |  |  | MEDICINE2 |
| Despesas de internamento | **Mts** |  |  |  |  |  |  |  | OVERNFEES2 |
| Presentes Monetários/Não monetários, contribuições | **Mts** |  |  |  |  |  |  |  | GIFTS2 |
| Custos de sustento (alimentos, bebidas) | **Mts** |  |  |  |  |  |  |  | SCOSTS2 |
| Outras despesas financeiras [ESPECIFIQUE ABAIXO] | **Mts** |  |  |  |  |  |  |  | OEXPENSE2 |
| *SE ‘Outras’ Especifique ________________________________________________*__________ | | | | | | | | | OCOSTS2 |

| Quanto tempo gastou no segundo local que procurou aconselhamento ou tratamento? Por favor, inclua ambos tempos de espera e da consulta. **[**Preencher ambos horas e minutos **Ex:. 45 mins = 00hrs, 45mins]** | | | |
| --- | --- | --- | --- |
| hrs |  |  | FACHRS2 |
| mins |  |  | FACMIN2 |

| Quantos ADULTOS, incluindo a si, acompanharam [NOME] para procurar aconselhamento/tratamento no segundo local? **[Apenas você=01]** |  |  | NUMACC2 |
| --- | --- | --- | --- |

| Quando levou [NOME] ao segundo local, foi lhe indicado outro sitio para aconselhamento/tratamento adicional? | | | |
| --- | --- | --- | --- |
| Sim | 1 |  | REFER2 |
| Não | 0 | **🡪 3.22** |  |

| **[Se SIM:]** Foi ao Terceiro lugar que te foi indicado? | | | |
| --- | --- | --- | --- |
| SIM | 1 | **🡪3.24** | GOREF2 |
| NÃO, procurei por aconselhamento ou tratamento em outro local diferente do que me foi indicado | 2 | **🡪3.23** |  |
| NÃO procurei nenhum aconselhamento ou tratamento fora de casa | 0 | **🡪3.29** |  |

| **[Se a resposta de 3.19 é NÃO:]** Mesmo que não tenha sido indicado, procurou aconselhamento ou tratamento em qualquer outro sítio depois de ir para o segundo sitio | | | |
| --- | --- | --- | --- |
| SIM | 1 |  | SEEK3 |
| NÃO | 0 | **🡪 3.29** |  |

| Porque razão procurou atendimento em um terceiro local? **[Apenas seleccione na razão principal]** | | | |
| --- | --- | --- | --- |
| Falta de medicamentos no segundo sitio | 1 |  | REAS3CS |
| Estava insatisfeito com o aconselhamento/ tratamento dado no segundo sitio | 2 |  |  |
| Outro [ESPECIFIQUE ABAIXO] | 88 |  |  |
| *Se ‘Outra’ especifique________________________________________________*__________ | | | REAS3CS**O** |

| Qual foi o terceiro sítio onde procurou aconselhamento ou tratamento? **[SE NA UNIDADE SANITÁRIA: Pergunte pelo nome da US e verifique o nível da US na lista de US fornecida]** | | | | |
| --- | --- | --- | --- | --- |
| 1 | **SECTOR PÚBLICO:** APE | 8 | **SECTOR PRIVADO:** Farmácia | RXTPLAC3U |
| 2 | **SECTOR PÚBLICO: Posto de Saúde** (PS) | 9 | **SECTOR PRIVADO:** Posto de Saúde Privado/Pequena Clinica |  |
| 3 | **SECTOR PÚBLICO: US de Nível I (CS I)** (CS I) | 10 | **SECTOR PRIVADO:** Hospital privado |  |
| 4 | **SECTOR PÚBLICO: US de Nível II (CS II)** | 11 | **SECTOR PRIVADO: Serviços móveis/ serviços de extensão** |  |
| 5 | **SECTOR PÚBLICO: US de Nível II (CS III)** | 12 | **OUTRO: Loja Geral** |  |
| 6 | Hospital | 13 | **OUTRO: Médico tradicional** |  |
| 7 | **SECTOR PÚBLICO: Serviços móveis/ serviços de extensão** | 88 | Outro [ESPECIFIQUE ABAIXO] |  |
| *Se ‘Outro’ specifyEspecifique________________________________________________*______________________________ | | | | RXTPLACO3 |

| Como chegou ao terceiro sitio onde solicitou tratamento ou aconselhamento? **[Seleccione no transporte principal]** | | | | | |
| --- | --- | --- | --- | --- | --- |
| 1  A pé | 2  Bicicleta | 3  Txopela/Taxi de motorizada | 4  Taxi (Transporte público/carro) | GOTPLAC3 | |
| Carro alugado | Carro alugado | 7  Camião/carrinha | 8  Barco |  |  |
| 88  Outro [ESPECIFIQUE ABAIXO] |  | | |  | |
| *Se ‘Outro’ especifique ________________________________________________*__________ | | | | GOTPLACO3 |  |

| Quanto tempo levou de casa para chegar ao terceiro sítio onde solicitou tratamento ou aconselhamento? **[**Preencher ambos horas e minutos **Ex: 45 mins = 00hrs, 45mins]** | | | |
| --- | --- | --- | --- |
| hrs |  |  | DURHRS3 |
| mins |  |  | MINS3 |

| Em relação à visita ao terceiro local onde procurou aconselhamento ou tratamento: Gastou dinheiro em qualquer um do seguinte: **[Exemplo 50 MT – 000050]**  **[Prencher 000000 se não tiver gasto dinheiro]**  **[Prencher 999999 se o valor é desconhecido]** | | | | | | | | | |
| --- | --- | --- | --- | --- | --- | --- | --- | --- | --- |
| Transporte | **Mts** |  |  |  |  |  |  |  | TRANS3 |
| Taxas de registo de pacientes | **Mts** |  |  |  |  |  |  |  | REFGEES3 |
| Custo de materiais (como luvas, IV, seringas, etc) | **Mts** |  |  |  |  |  |  |  | SYRINGES3 |
| Medicamentos | **Mts** |  |  |  |  |  |  |  | MEDICINE3 |
| Despesas de internamento | **Mts** |  |  |  |  |  |  |  | OVERNFEES3 |
| Presentes Monetários/Não monetários, contribuições | **Mts** |  |  |  |  |  |  |  | GIFTS3 |
| Custos de sustento (alimentos, bebidas) | **Mts** |  |  |  |  |  |  |  | SCOSTS3 |
| Outras despesas financeiras [ESPECIFIQUE ABAIXO] | **Mts** |  |  |  |  |  |  |  | OEXPENSE3 |
| *Se ‘Outras’ Especifique ________________________________________________*__________ | | | | | | | | | OCOSTS3 |

| Quanto tempo gastou no terceiro sítio onde procurou aconselhamento ou tratamento? Por favor, inclua ambos tempos de espera e da consulta. **[**Preencher ambos horas e minutos **Ex:. 45 mins = 00hrs, 45mins]** | | | |
| --- | --- | --- | --- |
| hrs |  |  | FACHRS3 |
| mins |  |  | FACMIN3 |

| Quantos ADULTOS, incluindo a si, acompanharam [NOME] para procurar aconselhamento/tratamento no terceiro local? **[Apenas você=01]** |  |  | NUMACC3 |
| --- | --- | --- | --- |

| **[O/A entrevistado/a procurou um APE para cuidar da criança?** **-Conferir as perguntas 3.3 (Primeiro local), 3.14 (Segundo local) ou 3.24 (Terceiro local). Se tiver procurado um APE para aconselhamento ou tratamento em alguma das perguntas, pule para 3.32]** **Se não tiver procurado um APE em qualquer das perguntas acima, então pergunte:** |
| --- |

| Porquê não procurou pelo APE? [**Seleccione apenas na razão principal]** | | | | | |
| --- | --- | --- | --- | --- | --- |
| 1  Não conheço o APE | 2  O APE não tem medicamentos | 3  APE muito caro | 4  APE muito longe | VISITVHT | |
| 5  APE não estava disponível | 6  APE não é bom/não confio | 7  Estava muito ocupado/a | 88  Outro especifique [ESPECIFIQUE ABAIXO] |  |  |
| *Se ‘Outro’ especifique ________________________________________________*__________ | | | | VISITO |  |

## **DIGA:**

Gostaria de lhe fazer algumas perguntas sobre o tempo que teve que gastar por dia cuidando da doença de [NOME]

| Primeiro me diga, [NOME] ainda padece da última doença ou já está recuperado? | | | |
| --- | --- | --- | --- |
| Continua doente | 1 |  | STILLSICK |
| Recuperou | 0 |  |  |

| Durante a última doença, durante quantos dias [NOME] esteve doente? **[Se continua doente, escreva o número de dias que a criança esteve doente até agora]** | **DAYS** |  |  | DAYSILL |
| --- | --- | --- | --- | --- |

| Durante os dias em que [NOME] esteve doente, **quanto tempo, em media, por dia gastou a cuidar do [NOME] em casa**? | **HRS** |  |  | HRSSILL |
| --- | --- | --- | --- | --- |
|  | **MINS** |  |  | MINSILL |

| Durante a última doença, quanto tempo por dia, em média, você teve que reduzir o tempo que gasta em suas outras atividades diárias para cuidar de [NOME] em casa? **[não deixe nada em branco:** escrever hrs = 00 minutos = 00 se não ocorreu mudanças no tempo gasto em atividades diárias**]** **-Explore com profundidade e explique que este valor expressa a redução de tempo de trabalho em:-**  **Exemplo: se o respondente normalmente trabalha durante 10 horas / dia na sua machamba, mas por causa de cuidar da criança durante a doença, só trabalha duas horas / dia na machamba, ele reduziu o seu tempo em 8 horas: escrever hrs = 08 , min = 00 para QN 3.36.2 -Se não costuma fazer um determinado tipo de trabalho ou NÃO houve mudança no tempo gasto em um tipo de trabalho, escrever hrs = 00 e 00 minutos** = | | | | | | |
| --- | --- | --- | --- | --- | --- | --- |
|  | Trabalho assalariado |  |  | hrs |  | SALRHRS |
|  |  |  |  | mins |  | SALRMIN |
|  |  |  |  |  |  |  |
|  | Trabalho na agricultura ou pesca |  |  | hrs |  | AGRRHRS |
|  |  |  |  | mins |  | AGRRMIN |
|  |  |  |  |  |  |  |
|  | Trabalho operário ocasional |  |  | hrs |  | LABRHRS |
|  |  |  |  | mins |  | LABRMIN |
|  |  |  |  |  |  |  |
|  | Trabalho por conta própria (incluindo motorista, comerciante, etc) |  |  | hrs |  | SELFRHRS |
|  |  |  |  | mins |  | SELFRMIN |
|  |  |  |  |  |  |  |
|  | Trabalho doméstico (Família) |  |  | hrs |  | DOMRHRS |
|  |  |  |  | mins |  | DOMRMIN |
|  |  |  |  |  |  |  |
|  | Outro trabalho [ESPECIFIQUE ABAIXO] |  |  | hrs |  | OTHRHRS |
|  |  |  |  | mins |  | OTHRMIN |
| Se ‘outro’ [especifique] ____________________________________________________ | |  | | | | REDOTH |

| Durante os dias em que [Nome] esteve doente, alguém além de você gastou tempo a cuidar de [Nome] em casa | | | |
| --- | --- | --- | --- |
| **NÃO** | 0 | **🡪3.39** | OTHCARE |
| SIM, Pai [ou mãe] da criança | 1 | **🡪3.38** |  |
| SIM, Irmã/o da criança | 2 |  |  |
| SIM, Avô da criança | 3 |  |  |
| SIM, outro | 88 |  |  |
| *Se ‘Outro’ Especifique ________________________________________________*__________ | | | OTHCAREO |

| Durante os dias em que [Nome] esteve doente **quanto tempo** em média por dia a pessoa gastou a cuidar de [NOME] em casa? | **HRS** |  |  | OHRSSILL |
| --- | --- | --- | --- | --- |
|  | **MINS** |  |  | OMINSILL |

| Em algum momento durante a doença, alguém fez a contagem respiratória da criança? **[MOSTRE O CONTADOR RESPIRATÓRIO, no entanto deixe claro que outros contadores podem ser usados]** | | | |
| --- | --- | --- | --- |
| SIM | 1 |  | CBREATHS |
| NÃO | 0 | **🡪3.40** |  |
| NÃO SEI | 99 |  |  |

| **[SE SIM]** Quem fez a contagem respiratória da criança **[Possível mais do que uma resposta - Seleccione no SIM/NÃO para cada opção (NÃO DEIXAR EM BRANCO)** | | | | | |
| --- | --- | --- | --- | --- | --- |
|  | APE | 1. SIM | 0. NÃO |  | VCOUNT |
|  | Pessoal do Posto/Centro | 1. SIM | 0. NÃO |  | HCCOUNT |
|  | Pessoal do Hospital | 1. SIM | 0. NÃO |  | HCOUNT |
|  | Pessoal da clinica/hospital privado | 1. SIM | 0. NÃO |  | PCOUNT |
|  | Pessoal da farmácia | 1. SIM | 0. NÃO |  | SCOUNT |
| Outro [Especifique] _________________________________________________________________________________ | | | | | WCOUNTO |

| Em algum momento durante a doença, [NOME] foi tirado sangue do dedo ou do calcanhar para testes? | | | |
| --- | --- | --- | --- |
| SIM | 1 |  | BLOOD |
| NÃO | 0 | **🡪S4** |  |
| Não SEI | 99 |  |  |

| **[SE SIM]** Por quem? **[Possível mais do que uma resposta - Seleccione no SIM/NÃO para cada opção]** | | | | | |
| --- | --- | --- | --- | --- | --- |
|  | APE | 1. SIM | 0. NÃO |  | VBLOOD |
|  | Pessoal do Posto/Centro | 1. SIM | 0. NÃO |  | HCBLOOD |
|  | Pessoal do Hospital | 1. SIM | 0. NÃO |  | HBLOOD |
|  | Pessoal da clinica/hospital privado | 1. SIM | 0. NÃO |  | PBLOOD |
|  | Pessoal da farmácia/ loja de medicamentos | 1. SIM | 0. NÃO |  | SBLOOD |
| Outro [Especifique] ________________________________________________________________________________ | | | | | WBLOODO |

| O teste de TDR era como este? [MOSTRAR TDR] | | | |
| --- | --- | --- | --- |
| SIM | 1 |  | RDTTEST |
| NÃO | 0 |  |  |
| Não sei | 99 |  |  |

| O exame de sangue deu positivo para a Malária? | | | |
| --- | --- | --- | --- |
| SIM | 1 |  | TESTPOS |
| NÃO | 0 |  |  |
| Não sei | 99 |  |  |

# Tratamento

| **DIGA:**  **Agora vou fazer-lhe algumas perguntas sobre qualquer tratamento que [NOME] recebeu durante esta última doença.** |
| --- |

| Em algum momento durante este último episódio de doença, [NOME] tomou algum medicamento para a doença? | | | |
| --- | --- | --- | --- |
| SIM | 1 |  | TOOKDRUG |
| NÃO | 0 | **🡪S5** |  |
| Não sei | 99 |  |  |

| **Se o respondente disse SIM, queremos descobrir exatamente quais os medicamentos que foram tomados.**  **Guie o respondente através das três opções nesta ordem:**   1. **Peça para ver o que restou de todo o tratamento tomado durante a última doença (por exemplo, cartela, pacote vazio)**   **DEPOIS:**   1. **Peça para ver a ficha de saúde da criança, ou receita médica, para verificar se algum outro tratamento foi dado. DEPOIS:**   **Mostre ao respondente os cartazes de medicamentos um por um para verificar a informação acima, OU para saber que tratamento foi dado se nenhuma receita médica ou medicamento restante existir.**   1. **NÃO APROFUNDE – Deixe o respondente mostrar ou identificar os medicamentos conforme se lembrar da doença** |
| --- |

| **[Seleccione no SIM para os medicamentos que o respondente identificou como tendo sido usados mesmo que tenham mostrado os medicamentos, receitas médicas ou cartelas de medicamentos usados). Senão Seleccione no Não (Não aprofunde).**  **Incluímos os códigos correspondentes a partir das cartelas de medicamentos ao lado de cada opção como ajuda para não se esquecer de usar as cartelas de medicamentos para confirmar todos os tratamentos.]**  **MOSTRE O CARTÃO 1:** |
| --- |

| **SRO** – D2 no Cartão | | | |
| --- | --- | --- | --- |
| SIM | 1 |  | D2ORS |
| NÃO | 0 | **🡪4.4** |  |

| **[SE SIM]** Onde conseguiu o SRO? | | | | | | | | | |
| --- | --- | --- | --- | --- | --- | --- | --- | --- | --- |
| 1  APE | 2  Posto/Centro de Saúde | 3  Hospital Público | | 4  Clinica/hospital privado | | 5  Farmácia | | EGOTORS | |
| 88  Outro [ESPECIFIQUE ABAIXO] |  | |  | |  | | |  |  |
| *Se ‘Outro’ especifique aqui ________________________________________________*___________________________ | | | | | | | EGOTORSO | |  |

| **ZINCO** – D3 no Cartão | | | |
| --- | --- | --- | --- |
| SIM | 1 |  | D3ZINC |
| NÃO | 0 | **🡪4.6** |  |

| **[SE SIM]** Onde conseguiu o **ZINCO**? | | | | | | | | | |
| --- | --- | --- | --- | --- | --- | --- | --- | --- | --- |
| 1  APE | 2  Posto/Centro de Saúde | 3  Hospital Público | | 4  Clinica/hospital privado | | 5  Farmácia | | EGOTZINC | |
| 88  Outro [ESPECIFIQUE ABAIXO] |  | |  | |  | | |  |  |
| *Se ‘Outro’ especifique aqui ________________________________________________*___________________________ | | | | | | | EGOTZNCO | |  |

| **[Se o respondente mencionar espontaneamente que usou SRO caseiro, Seleccione no SIM abaixo. Mais uma vez, não aprofunde]** |
| --- |

| **SRO CASEIRO** | | | |
| --- | --- | --- | --- |
| SIM | 1 |  | HMORS |
| NÃO | 0 |  |  |

| **METRONIDAZOLE** – D1 no cartão | | | |
| --- | --- | --- | --- |
| SIM | 1 |  | METRON |
| NÃO | 0 | **🡪4.9** |  |

| **[SE SIM]** Onde conseguiu o **METRONIDAZOLE**? | | | | | | | | |  |
| --- | --- | --- | --- | --- | --- | --- | --- | --- | --- |
| 1  APE | 2  Posto/Centro de Saúde | 3  Hospital Público | | 4  Clinica/hospital privado | | 5  Farmácia | | GOTMETR |  |
| 88  Outro [ESPECIFIQUE ABAIXO] |  | |  | |  | | |  |  |
| *Se ‘Outro’ especifique aqui ________________________________________________*___________________________ | | | | | | | GOTMETRO | | |

| **LEVAMISOL** – no cartão | | | |
| --- | --- | --- | --- |
| SIM | 1 |  | LEVA |
| NÃO | 0 | **🡪4.11** |  |

| **[IF SIM]** Onde conseguiu o **LEVAMISOL** ? | | | | | | | | |  |
| --- | --- | --- | --- | --- | --- | --- | --- | --- | --- |
| 1  APE | 2  Posto/Centro de Saúde | 3  Hospital Público | | 4  Clinica/hospital privado | | 5  Farmácia/Loja de medicamentos | | GOTLEVA |  |
| 88  Outro [ESPECIFIQUE ABAIXO] |  | |  | |  | | |  |  |
| *Se ‘Outro’ especifique aqui ________________________________________________*___________________________ | | | | | | | GOTLEVAO | | |

| **ALBENDAZOLE** – D5 no cartão | | | |
| --- | --- | --- | --- |
| SIM | 1 |  | ALBEN |
| NÃO | 0 | **🡪4.13** |  |

| **[SE SIM]** Onde conseguiu o **ALBENDAZOLE**? | | | | | | | | |  |
| --- | --- | --- | --- | --- | --- | --- | --- | --- | --- |
| 1  APE | 2  Posto/Centro de Saúde | 3  Hospital Público | | 4  Clinica/hospital privado | | 5  Farmácia | | GOTALBEN |  |
| 88  Outro [ESPECIFIQUE ABAIXO] |  | |  | |  | | |  |  |
| *Se ‘Outro’ especifique aqui ________________________________________________*___________________________ | | | | | | | GOTALBEO | | |

| **MEBENDAZOLE** – D6 no cartão | | | |
| --- | --- | --- | --- |
| SIM | 1 |  | MEBEN |
| NÃO | 0 | **🡪4.15** |  |

| **[SE SIM]** Onde conseguiu o **MEBENDAZOLE**? | | | | | | | | |  |
| --- | --- | --- | --- | --- | --- | --- | --- | --- | --- |
| 1  APE | 2  Posto/Centro de Saúde | 3  Hospital Público | | 4  Clinica/hospital privado | | 5  Farmácia | | GOTMEBE |  |
| 88  Outro [ESPECIFIQUE ABAIXO] |  | |  | |  | | |  |  |
| *Se ‘Outro’ especifique aqui ________________________________________________*___________________________ | | | | | | | GOTMEBO | | |

| **MOSTRE O CARTÃO 2:** | | | | |
| --- | --- | --- | --- | --- |
| **AMOXICILINA** – P1 ou P2 no cartão | | | |  |
| SIM | 1 |  | AMOXY |  |
| NÃO | 0 | **🡪4.17** |  |  |

| **[SE SIM]** Onde conseguiu a AMOXICILINA? | | | | | | | | |  |
| --- | --- | --- | --- | --- | --- | --- | --- | --- | --- |
| 1  APE | 2  Posto/Centro de Saúde | 3  Hospital Público | | 4  Clinica/hospital privado | | 5  Farmácia | | EGOTAMOX |  |
| 88  Outro [ESPECIFIQUE ABAIXO] |  | |  | |  | | |  |  |
| *Se ‘Outro’ especifique aqui ________________________________________________*___________________________ | | | | | | | EGOTAMXO | | |

| **AMPICILINA** – P3 no cartão | | | |
| --- | --- | --- | --- |
| SIM | 1 |  | AMPICIL |
| NÃO | 0 | **🡪4.19** |  |

| **[SE SIM]** Onde conseguiu a **AMPICILINA**? | | | | | | | | |  |
| --- | --- | --- | --- | --- | --- | --- | --- | --- | --- |
| 1  APE | 2  Posto/Centro de Saúde | 3  Hospital Público | | 4  Clinica/hospital privado | | 5  Farmácia | | GOTAMP |  |
| 88  Outro [ESPECIFIQUE ABAIXO] |  | |  | |  | | |  |  |
| *Se ‘Outro’ especifique aqui ________________________________________________*___________________________ | | | | | | | GOTAMPO | | |

| **COTRIMOXAZOLE** – P4 no cartão | | | |
| --- | --- | --- | --- |
| SIM | 1 |  | SEPTRIN |
| NÃO | 0 | **🡪4.21** |  |

| **[SE SIM]** Onde conseguiu o **COTRIMOXAZOLE**  ? | | | | | | | | |  |
| --- | --- | --- | --- | --- | --- | --- | --- | --- | --- |
| 1  APE | 2  Posto/Centro de Saúde | 3  Hospital Público | | 4  Clinica/hospital privado | | 5  Farmácia | | GOTSEPT |  |
| 88  Outro [ESPECIFIQUE ABAIXO] |  | |  | |  | | |  |  |
| *Se ‘Outro’ especifique aqui ________________________________________________*___________________________ | | | | | | | GOTSEPTO | | |

| CLORANFENICOL – P5 no cartão | | | |
| --- | --- | --- | --- |
| SIM | 1 |  | CHLORAM |
| NÃO | 0 | **🡪4.23** |  |

| **[SE SIM]** Onde conseguiu o CLORANFENICOL? | | | | | | | | |  |
| --- | --- | --- | --- | --- | --- | --- | --- | --- | --- |
| 1  APE | 2  Posto/Centro de Saúde | 3  Hospital Público | | 4  Clinica/hospital privado | | 5  Farmácia | | GOTCHLOR |  |
| 88  Outro [ESPECIFIQUE ABAIXO] |  | |  | |  | | |  |  |
| *Se ‘Outro’ especifique aqui ________________________________________________*___________________________ | | | | | | | GOTCHLO | | |

| ERITROMICINA – P6 no cartão | | | |
| --- | --- | --- | --- |
| SIM | 1 |  | ERYTHRO |
| NÃO | 0 | **🡪4.25** |  |

| **[SE SIM]** Onde conseguiu a ERITROMICINA? | | | | | | | | |  |
| --- | --- | --- | --- | --- | --- | --- | --- | --- | --- |
| 1  APE | 2  Posto/Centro de Saúde | 3  Hospital Público | | 4  Clinica/hospital privado | | 5  Farmácia | | GOTERYTH |  |
| 88  Outro [ESPECIFIQUE ABAIXO] |  | |  | |  | | |  |  |
| *Se ‘Outro’ especifique aqui ________________________________________________*___________________________ | | | | | | | GOTERYTO | | |

| **AZITROMICINA** – P7 no cartão | | | |
| --- | --- | --- | --- |
| SIM | 1 |  | AZYTHRO |
| NÃO | 0 | **🡪4.27** |  |

| **[SE SIM]** Onde conseguiu a **AZITROMICINA** ? | | | | | | | | |  |
| --- | --- | --- | --- | --- | --- | --- | --- | --- | --- |
| 1  APE | 2  Posto/Centro de Saúde | 3  Hospital Público | | 4  Clinica/hospital privado | | 5  Farmácia | | GOTAZY |  |
| 88  Outro [ESPECIFIQUE ABAIXO] |  | |  | |  | | |  |  |
| *Se ‘Outro’ especifique aqui ________________________________________________*___________________________ | | | | | | | GOTAZYO | | |

| **PEN V**– P8 no cartão | | | |
| --- | --- | --- | --- |
| SIM | 1 |  | PENV |
| NÃO | 0 | **🡪4.29** |  |

| **[SE SIM]** Onde conseguiu a **PEN V**? | | | | | | | | |  |
| --- | --- | --- | --- | --- | --- | --- | --- | --- | --- |
| 1  APE | 2  Posto/Centro de Saúde | 3  Hospital Público | | 4  Clinica/hospital privado | | 5  Farmácia | | GOTPENV |  |
| 88  Outro [ESPECIFIQUE ABAIXO] |  | |  | |  | | |  |  |
| *Se ‘Outro’ especifique aqui ________________________________________________*___________________________ | | | | | | | GOTPENO | | |

| CEFALEXINA – P9 no cartão | | | |
| --- | --- | --- | --- |
| SIM | 1 |  | CEPHA |
| NÃO | 0 | **🡪4.31** |  |

| **[IF SIM]** Onde conseguiu a CEFALEXINA ? | | | | | | | | |  |
| --- | --- | --- | --- | --- | --- | --- | --- | --- | --- |
| 1  APE | 2  Posto/Centro de Saúde | 3  Hospital Público | | 4  Clinica/hospital privado | | 5  Farmácia | | GOTCEPHA |  |
| 88  Outro [ESPECIFIQUE ABAIXO] |  | |  | |  | | |  |  |
| *Se ‘Outro’ especifique aqui ________________________________________________*___________________________ | | | | | | | GOTCEPHO | | |

| **MOSTRE O CARTÃO 3:** | | | | |
| --- | --- | --- | --- | --- |
| **COARTEM** ou ARTEMETER-LUMEFANTRINA – M1 no cartão | | | |  |
| SIM | 1 |  | M1COART |  |
| NÃO | 0 | **🡪4.33** |  |  |

| **[SE SIM]** Onde conseguiu o **COARTEM** ou ARTEMETER-LUMEFANTRINA ? | | | | | | | | |  |
| --- | --- | --- | --- | --- | --- | --- | --- | --- | --- |
| 1  APE | 2  Posto/Centro de Saúde | 3  Hospital Público | | 4  Clinica/hospital privado | | 5  Farmácia | | EGOTCOAR |  |
| 88  Outro [ESPECIFIQUE ABAIXO] |  | |  | |  | | |  |  |
| *Se ‘Outro’ especifique aqui ________________________________________________*___________________________ | | | | | | | EGOTCOAO | | |

| Dihydroartemisinin+ piperaquine.– M2 no cartão | | | |
| --- | --- | --- | --- |
| SIM | 1 |  | M2DUOC |
| NÃO | 0 | **🡪4.35** |  |

| **[SE SIM]** Onde conseguiu o Dihydroartemisinin+ piperaquine? | | | | | | | | |  |
| --- | --- | --- | --- | --- | --- | --- | --- | --- | --- |
| 1  APE | 2  Posto/Centro de Saúde | 3  Hospital Público | | 4  Clinica/hospital privado | | 5  Farmácia | | M2DUOCG |  |
| 88  Outro [ESPECIFIQUE ABAIXO] |  | |  | |  | | |  |  |
| *Se ‘Outro’ especifique aqui ________________________________________________*___________________________ | | | | | | | M2DUOCGO | | |

| **ARTESUNATO RECTAL**– M3 no cartão | | | |
| --- | --- | --- | --- |
| SIM | 1 |  | M3RECT |
| NÃO | 0 | **🡪4.37** |  |

| **SE SIM]** **]** Onde conseguiu o **ARTESUNATO RECTAL** ? | | | | | | | | |  |
| --- | --- | --- | --- | --- | --- | --- | --- | --- | --- |
| 1  APE | 2  Posto/Centro de Saúde | 3  Hospital Público | | 4  Clinica/hospital privado | | 5  Farmácia | | M3RECTG |  |
| 88  Outro [ESPECIFIQUE ABAIXO] |  | |  | |  | | |  |  |
| *Se ‘Outro’ especifique aqui ________________________________________________*___________________________ | | | | | | | M3RECTGO | | |

| **ARTEMETER** – M4 no cartão | | | |
| --- | --- | --- | --- |
| SIM | 1 |  | M4ARTM |
| NÃO | 0 | **🡪4.39** |  |

| **[SE SIM]** Onde conseguiu o **ARTEMETHER**? | | | | | | | | |  |
| --- | --- | --- | --- | --- | --- | --- | --- | --- | --- |
| 1  APE | 2  Posto/Centro de Saúde | 3  Hospital Público | | 4  Clinica/hospital privado | | 5  Farmácia | | M4ARTMG |  |
| 88  Outro [ESPECIFIQUE ABAIXO] |  | |  | |  | | |  |  |
| *Se ‘Outro’ especifique aqui ________________________________________________*___________________________ | | | | | | | M4ARTMGO | | |

| **FANSIDAR OU SP** – M5 no cartão | | | |
| --- | --- | --- | --- |
| SIM | 1 |  | M5FANS |
| NÃO | 0 | **🡪4.41** |  |

| **[SE SIM]** **]** Onde conseguiu o **FANSIDAR OU SP** ? | | | | | | | | |  |
| --- | --- | --- | --- | --- | --- | --- | --- | --- | --- |
| 1  APE | 2  Posto/Centro de Saúde | 3  Hospital Público | | 4  Clinica/hospital privado | | 5  Farmácia | | M5FANSG |  |
| 88  Outro [ESPECIFIQUE ABAIXO] |  | |  | |  | | |  |  |
| *Se ‘Outro’ especifique aqui ________________________________________________*___________________________ | | | | | | | M5FANSGO | | |

| **AMODIAQUINA** – M6 no cartão | | | |
| --- | --- | --- | --- |
| SIM | 1 |  | M6AMOD |
| NÃO | 0 | **🡪4.43** |  |

| **[IF SIM]** Onde conseguiu o **AMODIAQUINA**? | | | | | | | | |  |
| --- | --- | --- | --- | --- | --- | --- | --- | --- | --- |
| 1  APE | 2  Posto/Centro de Saúde | 3  Hospital Público | | 4  Clinica/hospital privado | | 5  Farmácia | | M6AMODG |  |
| 88  Outro [ESPECIFIQUE ABAIXO] |  | |  | |  | | |  |  |
| *Se ‘Outro’ especifique aqui ________________________________________________*___________________________ | | | | | | | M6AMODGO | | |

| **AMODIAQUINA-ARTESUNATO (ou artesunato-amodiaquina)** – M7 no cartão | | | |
| --- | --- | --- | --- |
| SIM | 1 |  | M7AMTE |
| NÃO | 0 | **🡪4.45** |  |

| **[SE SIM]** Onde conseguiu o a **AMODIAQUINA-ARTESUNATO**? | | | | | | | | |  |
| --- | --- | --- | --- | --- | --- | --- | --- | --- | --- |
| 1  APE | 2  Posto/Centro de Saúde | 3  Hospital Público | | 4  Clinica/hospital privado | | 5  Farmácia | | M7AMTEG |  |
| 88  Outro [ESPECIFIQUE ABAIXO] |  | |  | |  | | |  |  |
| *Se ‘Outro’ especifique aqui ________________________________________________*___________________________ | | | | | | | M7AMTEGO | | |

| **QUININO** – M8 no cartão | | | |
| --- | --- | --- | --- |
| SIM | 1 |  | M8QUIN |
| NÃO | 0 | **🡪4.47** |  |

| **[SE SIM]** Onde conseguiu o **QUININO**? | | | | | | | | |  |
| --- | --- | --- | --- | --- | --- | --- | --- | --- | --- |
| 1  APE | 2  Posto/Centro de Saúde | 3  Hospital Público | | 4  Clinica/hospital privado | | 5  Farmácia | | M8QUING |  |
| 88  Outro [ESPECIFIQUE ABAIXO] |  | |  | |  | | |  |  |
| *Se ‘Outro’ especifique aqui ________________________________________________*___________________________ | | | | | | | M8QUINGO | | |

| **CLOROQUINA** – M9 no cartão | | | |
| --- | --- | --- | --- |
| SIM | 1 |  | M9CHLO |
| NÃO | 0 | **🡪4.49** |  |

| **[SE SIM]** Onde conseguiu a **CLOROQUINA**? | | | | | | | | |  |
| --- | --- | --- | --- | --- | --- | --- | --- | --- | --- |
| 1  APE | 2  Posto/Centro de Saúde | 3  Hospital Público | | 4  Clinica/hospital privado | | 5  Farmácia | | M9CHLOG |  |
| 88  Outro [ESPECIFIQUE ABAIXO] |  | |  | |  | | |  |  |
| *Se ‘Outro’ especifique aqui ________________________________________________*___________________________ | | | | | | | M9CHLOGO | | |

| **ARTESUNATO**– M10 no cartão | | | |
| --- | --- | --- | --- |
| SIM | 1 |  | M10ART |
| NÃO | 0 | **🡪4.51** |  |

| **[SE SIM]** Onde conseguiu o **ARTESUNATO** ? | | | | | | | | | | | | |  |
| --- | --- | --- | --- | --- | --- | --- | --- | --- | --- | --- | --- | --- | --- |
| 1  APE | 2  Posto/Centro de Saúde | 3  Hospital Público | | 4  Clinica/hospital privado | | 5  Farmácia | | | | | M10ARTG | |  |
| 88  Outro [ESPECIFIQUE ABAIXO] |  | |  | |  | | | | | |  |  |  |
| *Se ‘Outro’ especifique aqui ________________________________________________*___________________________ | | | | | | | | | | M10ARTGO | | | |
| **ARTESUNATO-FANSIDAR** – não no cartão | | | | | | | | | | | |  |  |
| SIM | | | | | | | 1 |  | ART | | |  |  |
| NÃO | | | | | | | 0 | **🡪4.53** |  |  |  |  |  |

| **[[SE SIM]** Onde conseguiu o **ARTESUNATO-FANSIDAR**? | | | | | | | | |  |
| --- | --- | --- | --- | --- | --- | --- | --- | --- | --- |
| 1  APE | 2  Posto/Centro de Saúde | 3  Hospital Público | | 4  Clinica/hospital privado | | 5  Farmácia/ | | GOTART |  |
| 88  Outro [ESPECIFIQUE ABAIXO] |  | |  | |  | | |  |  |
| *Se ‘Outro’ especifique aqui ________________________________________________*___________________________ | | | | | | | GOARTO | | |

-----------------------------------------------------------------------------------------------------------------------------------------------------------

| **TRATAMENTO NA UNIDADE SANITÁRIA****[Verifique se a criança foi levada para uma unidade sanitária pública ou privada para esta doença (veja as perguntas anteriores 3.3, 3.14, e 3.24).****Se em nenhum momento a criança foi levada para a unidade sanitária, PULE PARA A PERGUNTA 4.55.** **Se foi a unidade sanitária, pergunte:]**Em algum momento durante a doença de [NOME]’S ficou de baixa na unidade sanitária? | | | |
| --- | --- | --- | --- |
| SIM | 1 |  | ADMIT |
| NÃO | 0 | **🡪4.55** |  |

| **[SE SIM:]** Quando [NOME] ficou de baixa, recebeu soro, ou talvez foi administrado medicamento na veia? | | | |
| --- | --- | --- | --- |
| SIM | 1 |  | PARENTE |
| NÃO | 0 |  |  |
| Não sei | 99 |  |  |

| **Está ultima vez que a/o [NOME] esteve doente, quantos dias depois de notar pela primeira vez que ela/ele está doente, começou o tratamento? [ex. Tratamento recebido do APE / unidade sanitária / farmácia]** | **DAYS** |  |  | TDAYS |
| --- | --- | --- | --- | --- |

# Encaminhamentos ao APE

**[Esta seção é para ser preenchida apenas para os entrevistados que mencionaram que procuraram aconselhamento ou tratamento de um/a APE nas perguntas 3.3 (primeiro sitio),** 3.14 **(segundo sitio) ou** 3.24 **(terceiro sitio). Verifique estas três perguntas agora Se a opção de APE não foi selecionada em nenhuma delas, coloque uma linha dupla por toda secção 5 e termine a entrevista.**

**Se a opção APE foi selecionada em alguma pergunta sobre a procura por cuidados de saúde, DIGA:**

**Agora, gostaria de pedir-lhe um pouco mais de detalhes sobre o serviço que recebeu quando visitou o APE a procura de aconselhamento ou tratamento.**

**Poderá notar que algumas das perguntas que estou prestes a fazer são semelhantes às perguntas que fiz anteriormente, mas aqui eu estou interessado no serviço que você recebeu do APE SOMENTE:**

| Disse que levou [Nome] ao APE. Durante a visita, o/a APE transferiu o/a [Nome] para uma unidade sanitária? | | | |
| --- | --- | --- | --- |
| SIM | 1 |  | REFVHT |
| NÃO | 0 | 🡪 **END** |  |

| Qual foi a principal razão que o APE alegou para a transferência? | | | |
| --- | --- | --- | --- |
| A criança estava muito doente e precisava de tratamento na unidade sanitária | 1 |  | REFWHY |
| O APE podia tratar a criança mas tinha acabado os medicamentos | 2 |  |  |
| O APE não disse a razão | 3 |  |  |
| O APE disse a razão mas não me lembro | 4 |  |  |
| Outra [ESPECIFIQUE ABAIXO] | 88 |  |  |
| *SE ‘outra’ especifique________________________________________________*_______________________ | |  | REFWHYO |

| **Será que o/a APE deu-lhe uma guia de transferência como esta**? **[Mostrar uma guia de transferência do APE se tiver alguma]** | | | |
| --- | --- | --- | --- |
| SIM | 1 |  | REFSLIP |
| NÃO | 0 |  |  |

| O APE deu uma primeira dose de medicamento à criança na visita, OU deu tratamento para a criança tomar antes de chegar à unidade sanitária, OU nenhum tratamento foi dado pelo APE | | | |
| --- | --- | --- | --- |
| O APE deu uma primeira dose de medicamento à criança na visita, | 1 |  | REFMED |
| O APE deu tratamento para a criança tomar antes de chegar à unidade sanitária | 2 |  |  |
| Nenhum tratamento foi dado pelo APE | 0 |  |  |

| O APE ligou para a Unidade Sanitária a informar que você ia lá? | | | |
| --- | --- | --- | --- |
| SIM | 1 |  | REFCALL |
| NÃO | 0 |  |  |
| O APE ajudou-te a encontrar transporte para a Unidade Sanitária? | | | |
| SIM | 1 |  | REFTRAN |
| NÃO | 0 |  |  |
| Não aplicável – Não precisei de transporte | 99 |  |  |

| Levou a criança para a Unidade Sanitária? | | | |
| --- | --- | --- | --- |
| SIM | 1 | 🡪5.9 | REFTAKE |
| NÃO | 0 |  |  |

| Se não, Porquê não? [**NÃO FAÇA SUGESTÕES]** | | | | |
| --- | --- | --- | --- | --- |
| Falta de dinheiro | 1. Mencionado | 0. Não mencionado |  | RNOCASH2 |
| Falta de transporte | 1. Mencionado | 0. Não mencionado |  | RNOTRAN2 |
| Esposo/Esposa/membro da família não deixou | 1. Mencionado | 0. Não mencionado |  | RFORBID2 |
| Fez tratamento caseiro/tratamento com ervas/foi ao médico tradicional | 1. Mencionado | 0. Não mencionado |  | RHTERAT2 |
| Pensei que a criança estivesse bem / criança iria ficar melhor si | 1. Mencionado | 0. Não mencionado |  | RNOSEVE2 |
| *Se o/a entrevistado/a der uma outra razão, especifique ________________________________________________*_____________________________________ | | |  | RNOTHERO2 |

| **SE A CRIANÇA NÃO FOI LEVADA PARA A UNIDADE SANITÁRIA COLOQUE UMA LINHA DUPLA PELO RESTO DESTA SECÇÃO, TERMINE A ENTREVISTA E AGRADEÇA AO ENTREVISTADO.** |
| --- |

| Quanto tempo após o APE transferir o/a [NOME] conseguiu leva-lo/a unidade sanitária? | | | |
| --- | --- | --- | --- |
| Em 1 hora | 1 | 🡪**5.11** | REFWHEN |
| Depois de 1 hora, mas entre 1 e 3 horas | 2 |  |  |
| Depois de 3 horas, mas no mesmo dia | 3 |  |  |
| No dia seguinte | 4 |  |  |
| Dois ou mais dias depois | 5 |  |  |

| Se não no mesmo dia, porquê? [**NÃO FAÇA SUGESTÕES]** | | | | |
| --- | --- | --- | --- | --- |
| Falta de dinheiro | 1. Mencionado | 0. NÃO Mencionado |  | RNOCASH |
| Falta de transporte | 1. Mencionado | 0. NÃO Mencionado |  | RNOTRAN |
| Esposo/Esposa/membro da família não deixou | 1. Mencionado | 0. NÃO Mencionado |  | RFORBID |
| Fez tratamento caseiro/tratamento com ervas/foi ao médico tradicional | 1. Mencionado | 0. NÃO Mencionado |  | RHTERAT |
| Pensei que a criança estivesse bem / criança iria ficar melhor | 1. Mencionado | 0. NÃO Mencionado |  | RNOSEVE |
| *Se o/a entrevistado/a der uma outra razão, especifique ________________________________________________*_______________________ | | |  | RNOTHERO |

| Quando chegou a unidade sanitária (primeira), quão rapidamente foi atendido pelo profissional de saúde? | | | | |
| --- | --- | --- | --- | --- |
| 1  Em menos de 30 mins | 2  Mais do que 30 minutos, mas menos de 1 hora | 3  Mais do que 1hr, mas menos de 3 horas | 4  Mais de 3 horas depois | REFWAIT |

**FIM DO QUESTIONÁRIO DA CRIANÇA.**

**Verifique o seu questionário, em seguida agradeça ao entrevistado pelo seu tempo.**
