# Supplementary material for: Improving outcomes for children with malaria, diarrhoea and pneumonia in Mozambique: A cluster randomised controlled trial of the inSCALE technology innovation
Source: PLOS Digit Health. 2023 Jun 12;2(6):e0000235. doi: 10.1371/journal.pdig.0000235 (PMC10260254; doi:10.1371/journal.pdig.0000235)
Supplement: S3 Text — (DOCX) [file pdig.0000235.s003.docx]

**S3 Text – Restricted Randomisation Procedure and codes – inSCALE Mozambique**

**Improving community health worker treatment for malaria, diarrhoea and pneumonia in Mozambique through the inSCALE mHealth innovation: A cluster randomised controlled trial**

Seyi Soremekun, Karin Källander, Raghu Lingam, Ana-Cristina Castel Branco, Neha Batura, Daniel Strachan, Abel Muiambo, Nelson Salomao, Juliao Condoane, Fenias Benhane, Frida Kasteng, Anna Vassall, Zelee Hill, Guus ten Asbroek, Sylvia Meek, James Tibenderana; and Betty Kirkwood

**1. Rationale for restricted randomisation**

Restricted randomisation aims to balance intervention arms on key covariates related to the primary outcome. An alternative is simple (non-restricted), randomisation which poses an increased risk of chance imbalance between arms for key variables particularly in trials with a relatively small number of clusters (Moulton 2004). The primary study outcome was appropriate treatment of major childhood illness (diarrhoea, malaria and pneumonia). Restricted randomisation was based on indicators available at baseline thought to vary proportionally to appropriate treatment: percentages of households with sick children seeking care at a public health facility, percentage seeking care to an CHW, CHW motivation (composite) score and cost of care seeking at baseline.

**2. Data sources for indicators used in the restricted randomisation and procedure**

Data on restriction variables was collected for all 12 districts (clusters) from a sample of households and from all CHWs during a baseline survey in November-December 2012.

There are 924 possible unique ways (12! ÷ (6!*6!)) to sort 12 districts into a control group (group 1) and intervention group (group 2); the scheme to be picked at random would be from a sub-sample of these 924 where there was a good balance between the arms for:

1. CHW motivation score – no more than a **0.5 difference** between arms
2. Cost (log10) of care seeking – no more than a **0.1 difference** between arms
3. % care seeking to an CHW – no more than an **3.5% difference** between arms
4. % care seeking to a public facility – no more than a **5% difference** between arms

**Iterative random sorting:** In an iterative process, the clusters were sorted randomly into two groups and repeated- up to 100,000 times; 84 unique allocation schemes which met all balance criteria were saved.

**3. FINAL RANDOMISATION SCHEME**

**84/924 schemes fit all the above criteria.**

A scheme was picked at random from the final 84 schemes (seed=05042013, the date of the randomisation) as the final scheme for the Mozambique study (Table 1):

| **Control** | **Technology** |
| --- | --- |
| Funhalouro | Homoine |
| Govuro | Inharrime |
| Jangamo | Inhassouro |
| Panda | Mabote |
| Vilankulo | Massinga |
| Zavala | Morrumbene |

**Table S9: Final randomisation scheme for the inSCALE intervention in Mozambique 2013**

**Randomisation Procedure Code (Stata v 13)**

**********

*INSCALE Randomisation procedure Mozambique 2013: Restricted on 4 variables:

*facility care seeking rate, APE (CHW) care seeking rate, log cost of care seeking for households, and CHW motivation score

*March 2013, updated May 2013 and annotated Dec 2021

*SSoremekun

*NOTES*

*Using Cluster_m database (all cluster level summary vars used in randomisation)

*This do file creates a file containing all allocation schemes meeting criteria; using different balance criteria limits: allowed_tm1/...tm12

*final agreed set of balance criteria saved in file: allowed_tm10

**********

clear all

cd D:\inscale\mozambique

************************

*Part I - randomisation and restrictions

file open fh using allowed_tm10.txt, write replace

file write fh "allocation" _tab "ds1" _tab "ds2" _tab "ds3" _tab "ds4" _tab "ds5" _tab "ds6" _tab "ds7" _tab "ds8" ///

_tab "ds9" _tab "ds10" _tab "ds11" _tab "ds12"

forvalues i=1/100000 {

use cluster_m, clear

rename district ds

set seed `i'

generate random = runiform()

sort random

generate group = group(2)

tabstat facill apeill clusmotf cluslogcost if group==1, stat(mean) save

matrix stats=r(StatTotal)

tabstat facill apeill clusmotf cluslogcost if group==2, stat(mean) save

matrix stats2=r(StatTotal)

if stats[1, 1]-stats2[1, 1]>=-5 & stats[1, 1]-stats2[1, 1]<=5 & ///

stats[1, 2]-stats2[1, 2]>=-2.5 & stats[1, 2]-stats2[1, 2]<=2.5 & ///

stats[1, 3]-stats2[1, 3]>=-0.5 & stats[1, 3]-stats2[1, 3]<=0.5 & ///

stats[1, 4]-stats2[1, 4]>=-0.2 & stats[1, 4]-stats2[1, 4]<=0.2 {

*randomisation scheme long--> wide and into text file:

sort group ds

keep ds

gen number=_n

gen all=`i'

reshape wide ds, i(all) j(number)

file write fh _n (all) _tab (ds1) _tab (ds2) _tab (ds3) _tab (ds4) _tab (ds5) _tab (ds6) _tab (ds7) _tab (ds8) ///

_tab (ds9) _tab (ds10) _tab (ds11) _tab (ds12)

}

else {

continue

}

}

file close fh

insheet using allowed_tm10.txt,clear

// identical schemes sorted to be together + duplicates dropped

sort ds1-ds12

compress

gen dup=1 if ds1==ds1[_n+1] & ds2==ds2[_n+1] & ds3==ds3[_n+1] & ds4==ds4[_n+1] & ds5==ds5[_n+1] & ds6==ds6[_n+1] ///

& ds7==ds7[_n+1] & ds8==ds8[_n+1] & ds9==ds9[_n+1] & ds10==ds10[_n+1] & ds11==ds11[_n+1] & ds12==ds12[_n+1]

drop if dup==1

browse

save allowed_tm10, replace

!"C:\Users\seyi\AppData\Local\Google\Chrome\Application\chrome.exe" "http://www.youtube.com/watch?v=-_kwXNVCaxY"

stop

*For reference: e.g. of other notification sound files on machine:

!"C:\Program Files\Windows Media Player\wmplayer" "C:\Windows\Media\Raga\Windows Exclamation.wav"

!"C:\Program Files\Windows Media Player\wmplayer" C:\Windows\Media\tada.wav

**********************

*PART II: Check for bias in schemes meeting balance criteria

clear all

cd D:\inscale\mozambique

use allowed_tm10

file open bs using bias_m10.txt, write replace

file write bs "distr_1" _tab "distr_2" _tab "freq" _tab "arm"

local listall Funhalouro Govuro Homoine Inharrime Jangamo Massinga Inhassouro Mabote Morrumbene Panda Vilankulo Zavala

foreach dista in `listall' {

gen `dista'1=.

foreach var of varlist ds1-ds6 {

replace `dista'1=1 if `var'=="`dista'"

} // end of ds1-6 for 1st 'pair foreach'

gen `dista'2=.

foreach var of varlist ds7-ds12 {

replace `dista'2=1 if `var'=="`dista'"

} // end of ds7-ds12 for 1st 'pair foreach'

foreach distb in `listall' {

if "`distb'"=="`dista'" {

continue

}

else {

gen `distb'1=.

foreach var of varlist ds1-ds6 {

replace `distb'1=1 if `var'=="`distb'"

} // end of ds1-6 for 2nd 'pair foreach'

gen `distb'2=.

foreach var of varlist ds7-ds12 {

replace `distb'2=1 if `var'=="`distb'"

} // end of ds7-12 for 2nd 'pair foreach'

count if `dista'1==1 & `distb'1==1

local a=r(N)

count if `dista'2==1 & `distb'2==1

local b=r(N)

count

local all=r(N)

local t=`a'+`b'

if `t'>`all'*0.65 | `t'<`all'*0.35 {

disp "flagged: frequency of `dista' and `distb' is `t'/`all'"

file write bs _n ("`dista'") _tab ("`distb'") _tab (`t'/`all') _tab "both arms"

drop `distb'*

} // end of embedded if for write to file flagged pairs

else {

drop `distb'*

} // end of embedded else for write to file flagged pairs

} // end of first else after distb 'pair foreach'

} // end of distb 'pair foreach'

drop `dista'*

} // end of overall/dista 'pair foreach' - each pair will be assessed twice as both combinations (x+y and y+x)

file close bs

insheet using bias_m10.txt,clear

// Each problem pair is saved twice e.g. as zavala/funhalouro and funhalouro/zavala

// in order to identify duplicates: reshape, sort district, reshape back, drop

gen id=_n

reshape long distr_, i(id) j(newvar)

sort id distr_

by id: gen nv=_n

drop newvar

reshape wide distr_, i(id) j(nv) // now pairs are same way round,, can drop dups

duplicates drop distr_1 distr_2, force

sort freq

drop id

br //

save biasfinal10_mon, replace

!"C:\Program Files\Windows Media Player\wmplayer" C:\Windows\Media\tada.wav

*****************************

*Part III pick final scheme and save

use allowed_tm10, clear // tm10 was the criteria set picked, after 100000 runs 05-04-2013, low bias

set seed 05042013 // = date when final allocation picked randomly from allowed set number 9

generate random = runiform()

sort random

keep if _n==1

list allocation // show the scheme number that was picked

drop dup random allocation // have to drop all numeric variables added onto the end for sxpose

sxpose, clear // had to download the string version of "xpose" which does the same as excel's transpose (wide-->long but NOT stata way)

generate group = group(2) // back to the same two groups as before re-shaped wide in first foreach above

rename group arm

rename _var1 district // for merge with cluster_m.dta/motherchild_m etc

sort district

label data

label data "Final scheme 6102 picked after 100000 runs using criteria set #10 SS 042013"

save inscale_tm10_6102,replace

**Analysis codes (Stata v 13) – Primary and Secondary Outcomes**

**Appropriate treatment by condition and coverage of blood tests for malaria, adjusted for baseline variables**

(fever = suspected malaria; malaria = confirmed malaria; diarrhoea = diarrhoea = pneum = suspected pneumonia; testblood =blood test for malaria; whch_apptreat = whole child appropriate treatment)

xtlogit fever i.inscale bfeverdis bclupub bcluape clusmotf clus1logcost, re i(dist) or

xtlogit malaria i.inscale bmalardis bclupub bcluape clusmotf clus1logcost, re i(dist) or

xtlogit diarrhoea i.inscale bdiarrdis bclupub bcluape clusmotf clus1logcost, re i(dist) or

xtlogit pneum i.inscale bpneudis bclupub bcluape clusmotf clus1logcost, re i(dist) or

xtlogit testblood i.inscale bclupub bcluape clusmotf clus1logcost, re i(dist) or

xtlogit whch_apptreat i.inscale bclusapp clusmotf clus1logcost if provf==1, re i(dist) or

**Rates of care seeking by health provider type**

(vhtf =CHW first port of call; vhtany= CHW at any point pubany = public facility at any point; sector_priva = private sector provider at any point; nocare = no care sought)

xtlogit vhtf i.inscale bcluape_a bclupub bcluape clusmotf clus1logcost, re i(dist) or

xtlogit vhtany i.inscale bcluape_a bclupub bcluape clusmotf clus1logcost, re i(dist) or

xtlogit pubany i.inscale bclupub_a bclupub bcluape clusmotf clus1logcost, re i(dist) or

xtlogit sector_priva i.inscale bclupriv_a bclupub bcluape clusmotf clus1logcost, re i(dist) or // combine because numbers are few

xtlogit nocare i.inscale bclunocare bclupub bcluape clusmotf clus1logcost, re i(dist) or

**Appropriate treatment by condition**

(malapprop = suspected malaria; mal2pprop = confirmed malaria; diapprop = diarrhoea = pnapprop = suspected pneumonia with any eligible antibiotic; pnapprop_a = suspected pneumonia with amoxicillin)

xtlogit malapprop i.inscale bmalap bclupub bcluape clusmotf clus1logcost, re i(dist) or // in wide database only (and below to rows too)

xtlogit mal2pprop i.inscale bmal2ap bclupub bcluape clusmotf clus1logcost, re i(dist) or

xtlogit diapprop i.inscale bdiarzap bclupub bcluape clusmotf clus1logcost , re i(dist) or

xtlogit pnapprop i.inscale bpneuap bclupub bcluape clusmotf clus1logcost, re i(dist) or

xtlogit pnapprop_a i.inscale bpneuap_a bclupub bcluape clusmotf clus1logcost, re i(dist) or

**CHW outcomes**

(APEidentity = social identity score; APEMot = motivation score; perform = clinical knowledge score prop_leave = attrition; anydrug = stockouts of any drugs for common childhood illesse)

xtreg perform i.inscale bclusperf bclupub bcluape clusmotf clus1logcost, i(dist) re // final - in paper

xtreg APEidentity i.inscale bclupub bcluape clusmotf clus1logcost, i(dist) re // final - in paper

xtreg APEMot i.inscale bclupub bcluape clusmotf clus1logcost, i(dist) re // final - in paper

xtreg prop_leave i.inscale bclupub bcluape clusmotf clus1logcost, be i(dist) // - in paper

xtlogit anydrug i.inscale bclupub bcluape clusmotf clus1logcost, re i(dist) or // in paper
